# Comparative dementia risk with GLP1 receptor agonists, SGLT2 inhibitors, or DPP4 inhibitors: a population-based cohort study

**DOI:** 10.1186/s13195-025-01929-x

**Published:** 2025-12-20

**Authors:** Che-Yuan Wu, Wajd Alkabbani, Baiju R. Shah, Moira K. Kapral, Jodi D. Edwards, Colleen J. Maxwell, Walter Swardfager

**Affiliations:** 1 Department of Pharmacology and Toxicology, University of Toronto, 1 King’s College Circle, Toronto, ON M5S 1A8 Canada; 2 Dr. Sandra Black Centre for Brain Resilience and Recovery, Hurvitz Brain Sciences Program, Sunnybrook Research Institute, Toronto, ON Canada; 3 School of Pharmacy, University of Waterloo, Kitchener, ON Canada; 4 Division of Endocrinology, Department of Medicine, Sunnybrook Health Sciences Centre, Toronto, ON Canada; 5 Department of Medicine, University of Toronto, Toronto, ON Canada; 6 ICES, Toronto, ON Canada; 7 Division of General Internal Medicine, Department of Medicine, University of Toronto, Toronto, ON Canada; 8 Toronto General Hospital Research Institute, University Health Network, Toronto, ON Canada; 9 School of Epidemiology and Public Health, University of Ottawa, Ottawa, ON Canada; 10 ICES uOttawa, Ottawa, ON Canada; 11 School of Public Health Sciences, University of Waterloo, Waterloo, ON Canada; 12 KITE University Health Network Toronto Rehabilitation Institute, Toronto, ON Canada

**Keywords:** Sodium, Glucose cotransporter, 2 (SGLT2) inhibitor, Glucagon, Like peptide, 1 (GLP1) receptor agonist, Dipeptidyl peptidase, 4 (DPP4) inhibitors, Diabetes, Dementia

## Abstract

**Background:**

We aim to investigate comparative dementia risk associated with glucagon-like peptide-1 receptor agonists (GLP1-RAs), sodium-glucose cotransporter-2 inhibitors (SGLT2i), and dipeptidyl peptidase-4 inhibitors (DPP4i) in adults aged ≥ 60 years with type 2 diabetes.

**Methods:**

This target trial emulation cohort study used primary care electronic health records from the UK Clinical Practice Research Datalink. Initiators of GLP1-RAs, SGLT2i, or DPP4i aged ≥ 60 years with type 2 diabetes and without cognitive impairment were compared pairwise in three separate analyses. After propensity scores overlap weighting, 13,965 pairs of GLP1-RA versus DPP4i initiators (cohort entry: 2006–2022; mean age: 66.9 years), 25,533 pairs of SGLT2i versus DPP4i initiators (cohort entry: 2013–2022; mean age: 69.0 years), and 14,214 pairs of GLP1-RA versus SGLT2i initiators (cohort entry: 2013–2022; mean age: 67.9 years) were analyzed. The primary outcome was incident all-cause dementia. The primary analysis was an intention-to-treat analysis. A secondary as-treated analysis for continuous use was performed.

**Results:**

In the intention-to-treat analysis, dementia risk was not different between GLP1-RA and DPP4i initiators (hazard ratio [HR] 0.95, 95% confidence interval [CI] 0.87–1.04; rates: 6.29 versus 6.64 per 1000 person-years; mean follow-up: 6.54 years); however, continuous GLP1-RA versus DPP4i use was associated with a 21% lower risk (HR 0.79, 95% CI 0.64–0.97; rates: 3.64 versus 4.82; mean follow-up: 2.71 years). SGLT2i versus DPP4i initiation was associated with a 14% lower dementia risk in the intention-to-treat analysis (HR 0.86, 95% CI 0.79–0.94; rates: 4.83 versus 5.60; mean follow-up: 4.91 years), and the as-treated analysis showed greater risk reduction (HR 0.70, 95% CI 0.60–0.82; rates: 3.82 versus 5.46; mean follow-up: 2.43 years). Dementia risk was comparable between GLP1-RA versus SGLT2i in both intention-to-treat (HR 0.98, 95% CI 0.87–1.11; rates: 4.85 versus 4.95; mean follow-up: 5.09 years) and as-treated (HR 1.07, 95% CI 0.85–1.36; rates: 3.71 versus 3.56; mean follow-up: 2.40 years) analyses.

**Conclusions:**

In people with type 2 diabetes aged ≥ 60 years, SGLT2i are associated with reduced dementia risk, but dementia risk reduction associated with the GLP1-RAs studied is less certain.

**Supplementary Information:**

The online version contains supplementary material available at 10.1186/s13195-025-01929-x.

## Introduction

Diabetes elevates the risk of dementia, including Alzheimer’s disease (AD) and vascular dementia (VD) [1]. Dementia risk with diabetes might be attributed to the development of vascular risk factors, oxidative stress, cerebral insulin resistance, inflammation, and endothelial dysfunction [2, 3]. In people with diabetes, hypoglycemia may increase dementia risk [4, 5]. Although glucose-lowering medications may reduce dementia risk through glycemic control, these medications may confer differential benefits to the brain due to activities unique to their different mechanisms of action.

A growing number of clinical studies investigate dementia risk between users of different glucose-lowering medications. Guidelines for older adults with diabetes mention that sodium-glucose cotransporter-2 (SGLT2) inhibitors and glucagon-like peptide-1 (GLP1) receptor agonists might have favourable effects on cognition [6] based on a network meta-analysis [7]. However, in that network meta-analysis [7], there was high methodological inconsistency between included studies, which brings into question the validity of indirect comparisons across drug classes. To strengthen that evidence, rigorous studies comparing directly pairs of glucose-lowering therapies with similar indication are needed.

Common second-line therapies for type 2 diabetes include dipeptidyl peptidase-4 (DPP4) inhibitors, SGLT2 inhibitors, and GLP1 receptor agonists [8, 9]. Linagliptin, a DPP4 inhibitor, was shown to have no effect on cognition in the CARMELINA and CAROLINA trials [10, 11]. Active-comparator new-user cohort studies with an appropriate design have consistently demonstrated dementia risk reduction with SGLT2 inhibitor users with diabetes across different regions [12–20]. Although exploratory or post-hoc analyses of randomized trials [21, 22] suggest GLP1 receptor agonists versus placebo may protect against cognitive decline, complementary real-world evidence studies with rigorous methodology and sufficient events have been limited to three studies, [16, 23, 24] and the results are mixed. Also, the comparative effectiveness of GLP1 receptor agonists and SGLT2 inhibitors has been a growing interest, and several cohort studies with robust methodology have concluded comparable dementia risk between initiators of GLP1 receptor agonists and SGLT2 inhibitors [16, 25, 26].

The present study aims to investigate comparative dementia risk with initiators of GLP1 receptor agonists, SGLT2 inhibitors, or DPP4 inhibitors in people with type 2 diabetes aged ≥ 60 years, using real-world observational data from primary care practice records in the UK. We further provide a secondary analysis to examine these associations with continuous use, and perform exploratory analyses of specific dementia aetiologies as outcomes. To our knowledge, no rigorous cohort study has pairwise compared dementia risk of DPP4 inhibitors, SGLT2 inhibitors, and GLP1 receptor agonists, using the same source of data.

## Methods

### Data source

The conduct of the present study was approved by the Clinical Practice Research Datalink (CPRD) Research Data Governance (#23_002545), the Research Ethics Board of Sunnybrook Health Sciences Centre (PIN: 6013), and the Research Ethics Board of University of Toronto (#46463).

The present study used the UK CPRD GOLD and Aurum databases, which routinely collect linkable but de-identified longitudinal data on demographics, medical records (coded using the Read codes or SNOMED-CT classification), clinical measures, and prescriptions from primary care practices [27, 28]. The data were recorded with dates of events; as such, the cohort entry could be constructed based on prescription dates, a lookback window could be used to define pre-exposure eligibility and covariates, and the date of outcomes could be ascertained.

The UK CPRD data were linked to the Hospital Episode Statistics Admitted Patient Care (HES APC) and the Office for National Statistics (ONS) databases, but the linkage was available only in a subset of individuals. The HES APC contains dates of hospital admissions, associated inpatient diagnoses (coded using the International Statistical Classification of Diseases version 10 [ICD-10]), and procedures (coded using the UK Office of Population, Census and Surveys classification [OPCS]) [29]. The ONS contains dates of registered death and associated data [30].

### Study design & population

Using an active-comparator new-user cohort design [31, 32], we emulated three hypothetical active-controlled randomized trials comparing SGLT2 inhibitors, injectable GLP1 receptor agonists, and DPP4 inhibitors pairwise. The three glucose-lowering medications are common second-line therapies recommended by the guidelines [8, 9]. Supplementary Table 1 compares the hypothetical target trials and the emulated cohorts. An active-comparator new-user cohort design [31, 32] compares initiators of different active treatments by emulating an active-controlled randomized trial. An active-comparator new-user cohort design is less susceptible to healthy user bias and confounding by indication, because an active treatment with similar indication rather than non-users is the comparator [31, 32]. Target trial emulation intends to formulate a clear causal question, avoid biases that critically threaten internal validity (e.g. immortal time bias), and provide adequately robust observational evidence when randomized trials are not feasible or not yet available [33].

Individuals aged ≥ 60 years and prescribed injectable GLP1 receptor agonists (except weight loss formulations of liraglutide or semaglutide), SGLT2 inhibitors, or DPP4 inhibitors entered the cohort study. When GLP1 receptor agonists were compared to DPP4 inhibitors, the cohort entry started from January 1 st, 2007, to December 31 st, 2022. For the other two comparisons, the cohort entry started from January 1 st, 2013, to December 31 st, 2022. To avoid immortal time bias and selection bias [33, 34], the cohort entry date of each comparison was the first eligible prescription of either study drug within an individual.

To identify new users based on a 1-year washout period, only those with 1-year continuous registration before cohort entry were included. Individuals prescribed either treatment in the past year before cohort entry were excluded. Individuals prescribed multiple molecules within the same class or both treatments at cohort entry were excluded. When GLP1 receptor agonists were studied, individuals prescribed weight loss formations or oral semaglutide at baseline or in the past year before cohort entry were also excluded.

Only individuals with HbA1c ≥ 6.5% (or ≥ 48 mmol/mol) or a type 2 diabetes diagnosis at any time until cohort entry were included. Individuals with type 1 diabetes, thyroid cancer, multiple endocrine neoplasia, or end stage kidney disease at any time until cohort entry or individuals with dialysis or the latest eGFR < 20 mL/min/1.73m2 in the past year were excluded, because they were unlikely to receive SGLT2 inhibitors or GLP1 receptor agonists.

Individuals with dementia diagnoses, records of cognitive deficits (including unspecified or mild cognitive impairment, aphasia, apraxia, and agnosia), or cholinesterase inhibitor or memantine prescriptions at any time until cohort entry were excluded. Because a 1-year lag time was used to address disease latency and mitigate reverse causality resulting from delayed diagnosis or treatment effects [35, 36], those with records of dementia, cognitive deficits, cholinesterase inhibitors, or memantine within 1 year after cohort entry, or those with follow-up less than 1 year were excluded.

**Fig. 1:**
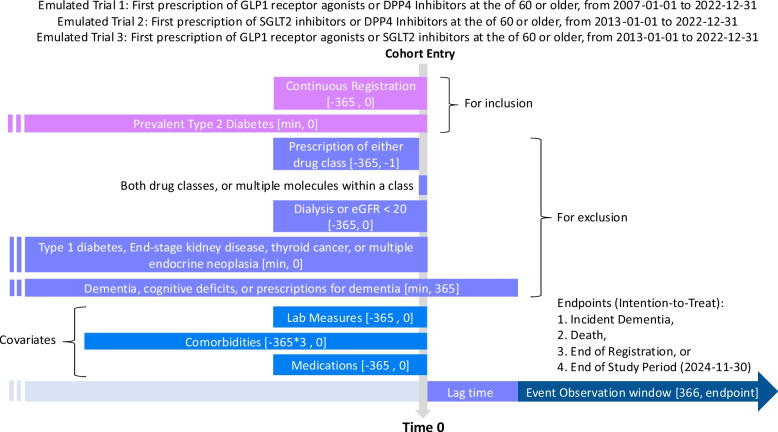
Graphical summary of study design

Both primary care data and HES APC were used to ascertain medical history. HbA1c, eGFR, and creatinine were extracted from primary care data. The Read codes, SNOMED-CT classification, and ICD codes for cognitive deficits and dementia are summarized in Supplementary Tables 2–6. Fig. 1 graphically summarizes the study design.

### Exposure definition

Dementia is a chronic outcome with long disease latency. To minimize the potential for informative censoring, the primary analysis used an intention-to-treat exposure definition.

Although an intention-to-treat analysis is less susceptible to informative censoring than an as-treated analysis, it is more prone to bias due to exposure misclassification. Thus, secondary as-treated analyses for continuous use were performed. Treatment discontinuation was defined as no subsequent prescription during the estimated or recorded days of supplies plus a 30-day grace period. The date of crossover was the date of prescription of the other study drug during follow-up.

### Outcome definition

The endpoint of the intention-to-treat analysis was incident dementia (or dementia subtypes in exploratory analyses), death (based on ONS or estimated dates from CPRD), end of CPRD registration, or end of study period (November 30th, 2024), whichever occurred first. In the as-treated analysis, the endpoint was the outcome of interest, 1 year after crossover or discontinuation, death, end of CPRD registration, or end of study period, whichever occurred first. The purpose of the 1-year extension from crossover or discontinuation was to reduce informative censoring caused by early symptoms or delayed diagnosis of dementia.

Incident all-cause dementia was defined as the date of first Read or SNOMED-CT codes for dementia (Supplementary Tables 6–7). Medical codes of dementia in CPRD were shown to have an 83% positive predictive value (PPV) [37].

Exploratory outcomes were AD and VD. The incident date was the date of the last record contributing to the definition of AD or VD described below.

AD was defined as: (1) an AD diagnosis with a prescription of cholinesterase inhibitors or memantine in any sequence, (2) an unspecified dementia code followed by two prescriptions of cholinesterase inhibitors or memantine, (3) two AD diagnoses on separate dates, (4) an AD diagnosis after a post-baseline record of cognitive assessment, referral to a related specialist (i.e., neurologist, geriatrician or psycho-geriatrician), or neuroimaging assessment (i.e., magnetic resonance imaging, computed tomography, or single photon emission computed tomography), OR (5) an AD diagnosis and a record of cognitive deficits in any sequence. This algorithm showed a 79% PPV for AD in a prior validation analysis [38].

VD was also defined as: (1) two VD diagnoses on separate dates, (2) a VD diagnosis or an unspecified dementia code within 2 years after a stroke diagnosis in primary care records or the primary position of a hospitalization, (3) a VD diagnosis after a post-baseline record of cognitive assessment, referral to a related specialist, or a neuroimaging assessment, OR (4) a VD diagnosis and a record of cognitive deficits in any sequence. This algorithm showed a 73% PPV for VD in a prior validation analysis [39].

### Statistical analysis

Analyses were performed using R 4.4.1 [40]. Propensity scores were estimated using a logistic regression conditional on 105 pre-exposure covariates listed in Supplementary Table 7, including demographics, laboratory measures, healthcare utilization, comorbidities, procedures, and concurrent medications among eligible individuals followed for at least 1 year and without dementia during the first year. Covariates were adjusted using the overlap weights, a propensity score weighting approach that generates no extreme weights and yields exact covariate balance for an average treatment effect in clinical equipoise [41].

Weighted cause-specific hazard ratios (HRs) with death as a competing risk and the 95% confidence intervals (CIs) were estimated from Cox models. Cause-specific hazard models are more appropriate for etiological questions [42]. Weighted Kaplan–Meier curves were used to visualize cumulative incidences and visualize whether the hazards might differ over time. Weighted incidence rate differences (IRDs) and the 95% CIs per 1,000 person-years were estimated, using a generalized linear model with an identity link function and Gaussian distribution [43]. Because a 1-year lag time was used, the at-risk time started at 1 year after cohort entry. Robust variance estimators were applied to account for weighting in regression models.

### Subgroup analysis

We performed subgroup analyses by age ≤ 70 years versus > 70 years, by males versus females, by insulin use, by atherosclerotic cardiovascular disease (i.e., myocardial infraction, angina, coronary artery disease, coronary revascularization, peripheral artery disease, aortic atherosclerotic disease, or cerebrovascular disease), by renal insufficiency (i.e., chronic kidney disease or the latest eGFR < 60 mL/min/1.73m^2^), and BMI ≥ 30 versus < 30 kg/m^2^. Age is a strong dementia risk factor. Females versus males are more likely to be diagnosed with dementia [44]. Insulin use was examined as a proxy for more advanced diabetes. Both SGLT2 inhibitors and GLP1 receptor agonists are recommended for those with atherosclerotic cardiovascular disease and chronic kidney disease, and GLP1 receptor agonists are recommended for people with obesity [8, 9]. These clinically relevant factors informed subgroup analyses.

Individual SGLT2 inhibitor molecules or GLP1 receptor agonist molecules were studied in separate cohorts. In each molecule-specific analysis, the cohort entry was restricted to the time that both treatments were on the market.

Propensity scores were re-estimated, and weights were re-calculated within subgroups and for molecule-specific analyses. Differences in IRDs or HRs between subgroups or across individual molecules were assessed by the Wald test for homogeneity with a 10% significance level.

### Sensitivity analysis

First, we restricted the follow-up time to a maximum of 5 years from cohort entry to mitigate exposure misclassifications under an intention-to-treat analysis, which can dilute the relative hazards when the follow-up time increases. Second, a 2-year lag time was used to more conservatively address reverse causality. Third, to examine robustness to a longer washout period, one sensitivity analysis excluded individuals with prior use of study drugs in the past 2 years, and the other analysis excluded individuals with prior use at any time. Fourth, Fine-Gray models with death as a competing risk were used to explore the ratio of cumulative incidence function estimates. Fifth, multiple imputation (20 iterations of 20 imputations) was applied to propensity score estimation, to explore the robustness to missing values in smoking, laboratory measures, body mass index (BMI), and number of hospitalizations. The Rubin’s rule was used to pool estimates from each iteration. Sixth, to examine robustness to a more specific outcome definition, incident dementia was defined as: a dementia record plus (1) another dementia record, (2) a record of cognitive deficits in any sequence, (3) a prescription of cholinesterase inhibitors or memantine in any sequence, OR (4) a post-baseline record of a cognitive test, referral to a related specialist, or neuroimaging assessment before a dementia record. Seventh, to examine the robustness to a more sensitive outcome definition, the date of first dementia diagnosis or initiation of cholinesterase inhibitors or memantine was defined as the incident date. Eighth, for statistically significant estimates, we performed a quantitative bias analysis using the rule-out approach to quantify the magnitude of an unmeasured confounder needed to explain away the observed association [45].

## Results

### Population characteristics

The first emulated trial included 22,042 GLP1 receptor agonist and 170,517 DPP4 inhibitor initiators, the second included 52,274 SGLT2 inhibitor and 129,471 DPP4 inhibitor initiators, and the third included 23,400 GLP1 receptor agonist and 92,280 SGLT2 inhibitor initiators (Supplementary Fig. 1). The propensity score distributions are depicted in Supplementary Figs. 2–4. The breakdown of molecules within each study drug class is summarized in Supplementary Table 8. All covariates had exact balance after weighting, and Supplementary Tables 9–11 summarize full baseline characteristics before and after weighting. Highlighted baseline characteristics after weighting are summarized in Table 1.

**Table 1:** Selected baseline characteristics of initiators of SGLT2 inhibitors versus DPP4 inhibitors, GLP1 receptor agonists versus DPP4 Inhibitors, or GLP1 Receptor Agonists versus SGLT2 Inhibitors After Propensity Score Weighting

Characteristics	GLP1 (*N* = 13,695)	DPP4 (*N* = 13,695)	SMD	SGLT2 (*N* = 25,533)	DPP4 (*N* = 25,533)	SMD	GLP1 (*N* = 14,214)	SGLT2 (*N* = 14,214)	SMD
Age in years, mean (SD)	66.9 (5.6)	66.9 (5.6)	0.000	69.0 (6.9)	69.0 (6.9)	0.000	67.9 (6.2)	67.9 (6.2)	0.000
Male	7615 (55.6%)	7615 (55.6%)	0.000	15,385 (60.3%)	15,385 (60.3%)	0.000	8106 (57.0%)	8106 (57.0%)	0.000
Smoking									
Ever	11,130 (81.3%)	11,130 (81.3%)	0.000	20,402 (79.9%)	20,402 (79.9%)	0.000	11,681 (82.2%)	11,681 (82.2%)	0.000
Never	2537 (18.5%)	2537 (18.5%)	0.000	5105 (20.0%)	5105 (20.0%)	0.000	2512 (17.7%)	2512 (17.7%)	0.000
Missing	27 (0.2%)	27 (0.2%)	0.000	25 (0.1%)	25 (0.1%)	0.000	20 (0.1%)	20 (0.1%)	0.000
Diabetes duration in years, mean (SD)	10.3 (6.2)	10.3 (6.2)	0.000	9.6 (6.4)	9.6 (6.4)	0.000	11.4 (6.5)	11.4 (6.5)	0.000
Body Mass Index, kg/m^2^									
≤ 24.9	185 (1.4%)	185 (1.4%)	0.000	2156 (8.4%)	2156 (8.4%)	0.000	250 (1.8%)	250 (1.8%)	0.000
25.0–29.9	1618 (11.8%)	1618 (11.8%)	0.000	6837 (26.8%)	6837 (26.8%)	0.000	1960 (13.8%)	1960 (13.8%)	0.000
≥ 30.0	10,919 (79.7%)	10,919 (79.7%)	0.000	13,961 (54.7%)	13,961 (54.7%)	0.000	10,854 (76.4%)	10,854 (76.4%)	0.000
Missing	971 (7.1%)	971 (7.1%)	0.000	2579 (10.1%)	2579 (10.1%)	0.000	1150 (8.1%)	1150 (8.1%)	0.000
HbA1c, %									
≤ 7.0	1230 (9.0%)	1230 (9.0%)	0.000	2749 (10.8%)	2749 (10.8%)	0.000	956 (6.7%)	956 (6.7%)	0.000
7.1–8.0	2932 (21.4%)	2932 (21.4%)	0.000	7112 (27.9%)	7112 (27.9%)	0.000	2582 (18.2%)	2582 (18.2%)	0.000
> 8.0	9196 (67.1%)	9196 (67.1%)	0.000	15,095 (59.1%)	15,095 (59.1%)	0.000	10,418 (73.3%)	10,418 (73.3%)	0.000
Missing	337 (2.5%)	337 (2.5%)	0.000	578 (2.3%)	578 (2.3%)	0.000	257 (1.8%)	257 (1.8%)	0.000
eGFR, mL/min/1.73m^2^									
≥ 90	5200 (38.0%)	5200 (38.0%)	0.000	10,384 (40.7%)	10,384 (40.7%)	0.000	5451 (38.4%)	5451 (38.4%)	0.000
60–90	5768 (42.1%)	5768 (42.1%)	0.000	11,463 (44.9%)	11,463 (44.9%)	0.000	6104 (42.9%)	6104 (42.9%)	0.000
45–60	1429 (10.4%)	1429 (10.4%)	0.000	1929 (7.6%)	1929 (7.6%)	0.000	1458 (10.3%)	1458 (10.3%)	0.000
30–45	586 (4.3%)	586 (4.3%)	0.000	990 (3.9%)	990 (3.9%)	0.000	726 (5.1%)	726 (5.1%)	0.000
< 30	91 (0.7%)	91 (0.7%)	0.000	241 (0.9%)	241 (0.9%)	0.000	158 (1.1%)	158 (1.1%)	0.000
Missing	621 (4.5%)	621 (4.5%)	0.000	526 (2.1%)	526 (2.1%)	0.000	316 (2.2%)	316 (2.2%)	0.000
Comorbidities									
Cerebrovascular disease	389 (2.8%)	389 (2.8%)	0.000	892 (3.5%)	892 (3.5%)	0.000	459 (3.2%)	459 (3.2%)	0.000
Peripheral artery or aortic atherosclerotic disease	492 (3.6%)	492 (3.6%)	0.000	801 (3.1%)	801 (3.1%)	0.000	522 (3.7%)	522 (3.7%)	0.000
Atrial fibrillation	1014 (7.4%)	1014 (7.4%)	0.000	2298 (9.0%)	2298 (9.0%)	0.000	1210 (8.5%)	1210 (8.5%)	0.000
Myocardial infarction	329 (2.4%)	329 (2.4%)	0.000	797 (3.1%)	797 (3.1%)	0.000	383 (2.7%)	383 (2.7%)	0.000
Angina	982 (7.2%)	982 (7.2%)	0.000	1603 (6.3%)	1603 (6.3%)	0.000	967 (6.8%)	967 (6.8%)	0.000
Heart failure	720 (5.3%)	720 (5.3%)	0.000	1968 (7.7%)	1968 (7.7%)	0.000	946 (6.7%)	946 (6.7%)	0.000
Coronary artery disease	1999 (14.6%)	1999 (14.6%)	0.000	3704 (14.5%)	3704 (14.5%)	0.000	2130 (15.0%)	2130 (15.0%)	0.000
Hypertension	7953 (58.1%)	7953 (58.1%)	0.000	14,365 (56.3%)	14,365 (56.3%)	0.000	8486 (59.7%)	8486 (59.7%)	0.000
Chronic kidney disease	2133 (15.6%)	2133 (15.6%)	0.000	3197 (12.5%)	3197 (12.5%)	0.000	2275 (16.0%)	2275 (16.0%)	0.000
Revascularization	1309 (9.6%)	1309 (9.6%)	0.000	2731 (10.7%)	2731 (10.7%)	0.000	1504 (10.6%)	1504 (10.6%)	0.000
Medications									
Metformin	12,330 (90.0%)	12,330 (90.0%)	0.000	22,718 (89.0%)	22,718 (89.0%)	0.000	12,499 (87.9%)	12,499 (87.9%)	0.000
Thiazolidinediones	2460 (18.0%)	2460 (18.0%)	0.000	1444 (5.7%)	1444 (5.7%)	0.000	1088 (7.7%)	1088 (7.7%)	0.000
Sulfonylureas	6865 (50.1%)	6865 (50.1%)	0.000	8700 (34.1%)	8700 (34.1%)	0.000	6931 (48.8%)	6931 (48.8%)	0.000
GLP1 receptor agonists	–	–	–	1026 (4.0%)	1026 (4.0%)	0.000	–	–	–
SGLT2 inhibitors	2306 (16.8%)	2306 (16.8%)	0.000	–	–	–	–	–	–
DPP4 inhibitors	–	–	–	–	–	–	7099 (49.9%)	7099 (49.9%)	0.000
Insulin	2388 (17.4%)	2388 (17.4%)	0.000	2150 (8.4%)	2150 (8.4%)	0.000	2701 (19.0%)	2701 (19.0%)	0.000
Statins	11,612 (84.8%)	11,612 (84.8%)	0.000	20,947 (82.0%)	20,947 (82.0%)	0.000	12,065 (84.9%)	12,065 (84.9%)	0.000
Angiotensin converting enzyme inhibitors	7282 (53.2%)	7282 (53.2%)	0.000	12,137 (47.5%)	12,137 (47.5%)	0.000	7244 (51.0%)	7244 (51.0%)	0.000
Angiotensin receptor blockers	3335 (24.3%)	3335 (24.3%)	0.000	5545 (21.7%)	5545 (21.7%)	0.000	3466 (24.4%)	3466 (24.4%)	0.000
Beta-blockers	4164 (30.4%)	4164 (30.4%)	0.000	7805 (30.6%)	7805 (30.6%)	0.000	4574 (32.2%)	4574 (32.2%)	0.000
Calcium channel blockers	5330 (38.9%)	5330 (38.9%)	0.000	9565 (37.5%)	9565 (37.5%)	0.000	5615 (39.5%)	5615 (39.5%)	0.000
Loop diuretics	2459 (18.0%)	2459 (18.0%)	0.000	3478 (13.6%)	3478 (13.6%)	0.000	2261 (15.9%)	2261 (15.9%)	0.000
Thiazide diuretics	3058 (22.3%)	3058 (22.3%)	0.000	4413 (17.3%)	4413 (17.3%)	0.000	2827 (19.9%)	2827 (19.9%)	0.000
Proton pump inhibitors	5977 (43.6%)	5977 (43.6%)	0.000	11,181 (43.8%)	11,181 (43.8%)	0.000	6755 (47.5%)	6755 (47.5%)	0.000

N was the sum of overlap weights. Full characteristics before and after weighting are presented in the supplementary material

*Abbreviations GLP1* Glucagon-like peptide-1, *SGLT2* Sodium-glucose cotransporter 2, *DPP4* Dipeptidyl peptidase-4, *SMD* Standardized mean difference, *SD* Standard deviation

### Dementia risk

The crude hazard ratios (HRs), incidence rates, and incidence rate differences (IRDs) are reported in Supplementary Table 12. The observed study endpoints are summarized in Supplementary Table 13. When DPP4 inhibitors were a comparator, 74.8% GLP1 receptor agonist initiators discontinued the treatment without other endpoints over a median follow-up of 1.31 years on treatment; however, only 56.7% SGLT2 inhibitor initiators discontinued the treatment without other endpoints over a median follow-up of 1.28 years on treatment (Supplementary Table 12 and 13).

**Fig. 2:**
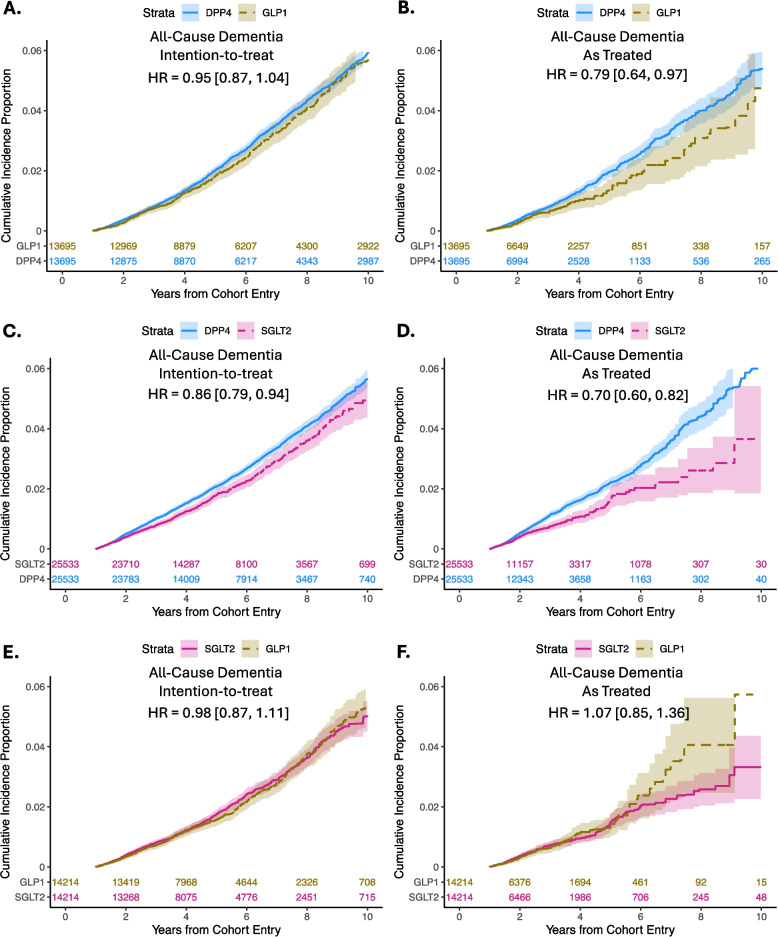
Weighted Kaplan–Meier Curves of Incident All-Cause Dementia with GLP1 Receptor Agonists versus DPP4 Inhibitors, SGLT2 Inhibitors versus DPP4 Inhibitors, or GLP1 Receptor Agonists versus SGLT2 Inhibitors

The weighted HRs, incidence rates, and IRDs for all-cause dementia, AD, and VD are summarized in Table 2. In the intention-to-treat analysis, GLP1 receptor agonist versus DPP4 inhibitor initiation was not associated with lower incident all-cause dementia (HR [95% CI]: 0.95 [0.87–1.04]; Fig. 2A); however, the as-treated analysis found a 21% lower risk with continuous use (HR [95% CI]: 0.79 [0.64–0.97]; Fig. 2B).

**Table 2:** Dementia Risks Associated with GLP1 receptor agonists versus DPP4 Inhibitors, SGLT2 Inhibitors versus DPP4 Inhibitors, and GLP1 Receptor Agonists versus SGLT2 Inhibitors After Propensity Score Weighting

Outcome	Exposure	N ^a^	Median Follow-Up Time [IQR], years ^b^	Total Person-Years At Risk	Events	Rates ^c^	IRD [95% CI] ^c^	HR [95% CI]
GLP1 receptor agonists versus DPP4 Inhibitors (Intention-to-Treat)								
All-Cause Dementia	GLP1	13,695	5.47 [3.24, 9.24]	75,595.01	476	6.29	−0.35 [−0.91, 0.21]	0.95 [0.87, 1.04]
	DPP4	13,695	5.52 [3.27, 9.31]	76,190.49	506	6.64	0 [Reference]	1 [Reference]
Alzheimer’s disease	GLP1	13,695	5.53 [3.27, 9.34]	76,401.96	213	2.79	−0.05 [−0.42, 0.32]	0.99 [0.87, 1.13]
	DPP4	13,695	5.59 [3.29, 9.42]	77,091.02	219	2.84	0 [Reference]	1 [Reference]
Vascular Dementia	GLP1	13,695	5.53 [3.27, 9.38]	76,566.00	161	2.10	−0.11 [−0.43, 0.21]	0.96 [0.82, 1.11]
	DPP4	13,695	5.60 [3.30, 9.45]	77,239.54	171	2.21	0 [Reference]	1 [Reference]
GLP1 receptor agonists versus DPP4 Inhibitors (As Treated)								
All-Cause Dementia	GLP1	13,695	1.95 [1.31, 3.17]	22,226.67	81	3.64	−1.18 [−1.98, −0.39]	0.79 [0.64, 0.97]
	DPP4	13,695	2.03 [1.37, 3.37]	24,846.40	120	4.82	0 [Reference]	1 [Reference]
Alzheimer’s disease	GLP1	13,695	1.95 [1.31, 3.18]	22,288.93	30	1.34	−0.39 [−0.86, 0.08]	0.84 [0.60, 1.19]
	DPP4	13,695	2.04 [1.37, 3.38]	24,990.41	43	1.73	0 [Reference]	1 [Reference]
Vascular Dementia	GLP1	13,695	1.95 [1.31, 3.18]	22,306.64	20	0.92	−0.45 [−0.86, −0.05]	0.71 [0.47, 1.07]
	DPP4	13,695	2.04 [1.37, 3.38]	25,005.07	34	1.37	0 [Reference]	1 [Reference]
SGLT2 inhibitors versus DPP4 inhibitors (Intention-to-Treat)								
All-Cause Dementia	SGLT2	25,533	4.52 [2.86, 6.64]	100,036.37	483	4.83	−0.77 [−1.21, −0.34]	0.86 [0.79, 0.94]
	DPP4	25,533	4.40 [2.85, 6.61]	99,569.97	558	5.60	0 [Reference]	1 [Reference]
Alzheimer’s disease	SGLT2	25,533	4.57 [2.88, 6.67]	100,600.94	205	2.04	−0.52 [−0.81, −0.24]	0.80 [0.70, 0.91]
	DPP4	25,533	4.44 [2.86, 6.64]	100,155.98	256	2.56	0 [Reference]	1 [Reference]
Vascular Dementia	SGLT2	25,533	4.57 [2.88, 6.68]	100,679.16	191	1.89	−0.01 [−0.28, 0.26]	0.99 [0.86, 1.14]
	DPP4	25,533	4.45 [2.86, 6.65]	100,316.59	191	1.90	0 [Reference]	1 [Reference]
SGLT2 inhibitors versus DPP4 inhibitors (As Treated)								
All-Cause Dementia	SGLT2	25,533	1.77 [1.27, 2.86]	34,888.12	133	3.82	−1.64 [−2.32, −0.97]	0.70 [0.60, 0.82]
	DPP4	25,533	1.95 [1.32, 3.02]	37,889.25	207	5.46	0 [Reference]	1 [Reference]
Alzheimer’s disease	SGLT2	25,533	1.78 [1.27, 2.87]	35,006.27	41	1.18	−1.04 [−1.42, −0.65]	0.54 [0.41, 0.71]
	DPP4	25,533	1.95 [1.32, 3.03]	38,035.51	84	2.22	0 [Reference]	1 [Reference]
Vascular Dementia	SGLT2	25,533	1.78 [1.27, 2.87]	35,006.74	41	1.17	−0.65 [−1.03, −0.28]	0.64 [0.48, 0.86]
	DPP4	25,533	1.95 [1.32, 3.03]	38,067.88	69	1.82	0 [Reference]	1 [Reference]
GLP1 receptor agonists versus SGLT2 Inhibitors (Intention-to-Treat)								
All-Cause Dementia	GLP1	14,214	4.56 [2.95, 6.83]	57,949.81	281	4.85	−0.10 [−0.67, 0.47]	0.98 [0.87, 1.11]
	SGLT2	14,214	4.70 [2.95, 6.96]	58,424.46	289	4.95	0 [Reference]	1 [Reference]
Alzheimer’s disease	GLP1	14,214	4.62 [2.97, 6.85]	58,281.60	126	2.16	−0.07 [−0.45, 0.31]	0.97 [0.82, 1.16]
	SGLT2	14,214	4.72 [2.97, 6.99]	58,786.54	131	2.23	0 [Reference]	1 [Reference]
Vascular Dementia	GLP1	14,214	4.63 [2.97, 6.86]	58,349.48	96	1.65	−0.10 [−0.43, 0.23]	0.95 [0.78, 1.16]
	SGLT2	14,214	4.73 [2.97, 7.00]	58,864.87	103	1.75	0 [Reference]	1 [Reference]
GLP1 receptor agonists versus SGLT2 Inhibitors (As Treated)								
All-Cause Dementia	GLP1	14,214	1.83 [1.29, 2.86]	19,073.72	71	3.71	0.15 [−0.70, 1.01]	1.07 [0.85, 1.36]
	SGLT2	14,214	1.84 [1.29, 2.98]	20,676.12	74	3.56	0 [Reference]	1 [Reference]
Alzheimer’s disease	GLP1	14,214	1.83 [1.29, 2.87]	19,122.15	25	1.31	0.09 [−0.42, 0.60]	1.13 [0.76, 1.69]
	SGLT2	14,214	1.85 [1.29, 2.98]	20,743.31	25	1.22	0 [Reference]	1 [Reference]
Vascular Dementia	GLP1	14,214	1.83 [1.29, 2.86]	19,120.94	25	1.30	0.28 [−0.19, 0.76]	1.31 [0.88, 1.97]
	SGLT2	14,214	1.85 [1.29, 2.98]	20,745.54	21	1.01	0 [Reference]	1 [Reference]

*Abbreviations GLP1* Glucagon-like peptide-1, *SGLT2* Sodium-glucose cotransporter 2, *DPP4* Dipeptidyl peptidase-4, *IQR* Interquartile range, *IRD* Incidence rate difference, *HR* Hazard ratio, *CI* Confidence interval

^a^Sum of overlap weights

^b^Reported follow-up time started from cohort entry

^c^Per 1,000 person-years

In the intention-to-treat analysis, SGLT2 inhibitor versus DPP4 inhibitor initiation was associated with a 14% lower risk of all-cause dementia (HR [95% CI]: 0.86 [0.79–0.94]; Fig. 2C). In the as-treated analysis, continuous use of SGLT2 inhibitors was associated with a 30% lower risk of all-cause dementia (HR [95% CI]: 0.70 [0.60–0.82]; Fig. 2D).

Incident all-cause dementia was comparable between those on GLP1 receptor agonists versus SGLT2 inhibitors in the intention-to-treat analysis (HR [95% CI]: 0.98 [0.87–1.11]; Fig. 2E) and in the as-treated analysis (HR [95% CI]: 1.07 [0.85–1.36]; Fig. 2F).

Continuous use of SGLT2 inhibitor versus DPP4 inhibitor initiation was associated with a lower risk of AD (HR [95% CI]: 0.54 [0.41–0.71]) and VD (HR [95% CI]: 0.64 [0.48–0.85]). Continuous use of GLP1 receptor agonists versus DPP4 inhibitors did not show statistically significant estimates for AD (HR [95% CI]: 0.84 [0.60–1.19]) and VD (HR [95% CI]: 0.71 [0.47–1.07]). The risks of AD (HR [95% CI]: 1.13 [0.76–1.69]) and VD (HR [95% CI]: 1.31 [0.88–1.97]) were not significantly higher with continuous use of GLP1 receptor agonists versus SGLT2 inhibitors.

### Subgroup analysis

**Fig. 3:**
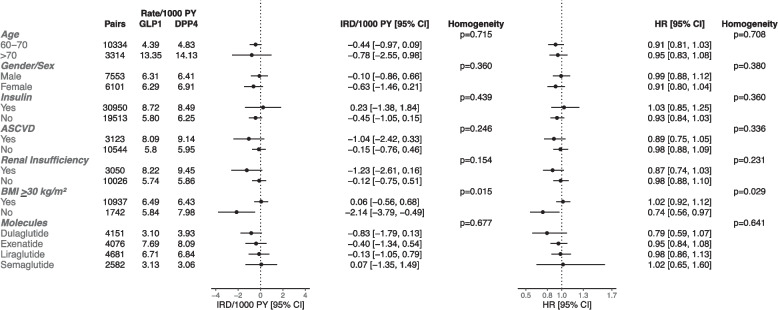
The risk of all-cause dementia with GLP1 Receptor Agonists versus DPP4 Inhibitors in subgroups (Intention-to-Treat). HR = hazard ratio; IRD = incidence rate difference; PY = person-years; CI = confidence interval; ASCVD = atherosclerotic cardiovascular disease; BMI = body mass index

**Fig. 4:**
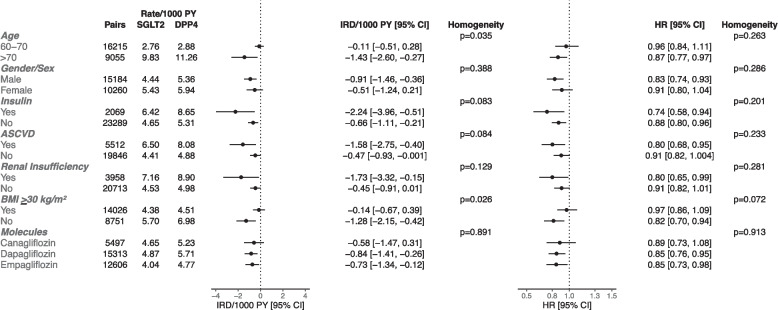
The risk of all-cause dementia with SGLT2 Inhibitors versus DPP4 Inhibitors in subgroups (Intention-to-Treat). HR = hazard ratio; IRD = incidence rate difference; PY = person-years; CI = confidence interval; ASCVD = atherosclerotic cardiovascular disease; BMI = body mass index

**Fig. 5:**
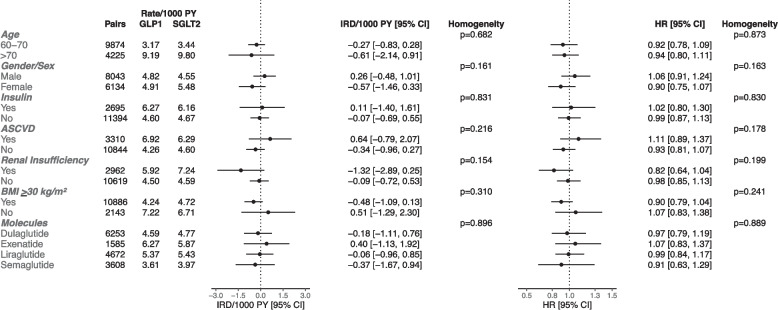
The risk of all-cause dementia with GLP1 Receptor Agonists versus SGLT2 Inhibitors in subgroups (Intention-to-Treat). HR = hazard ratio; IRD = incidence rate difference; PY = person-years; CI = confidence interval; ASCVD = atherosclerotic cardiovascular disease; BMI = body mass index

The molecule-specific and subgroup analyses are summarized in Figs. 3, 4, 5. GLP1 receptor agonist versus DPP4 inhibitor initiation was associated with a lower risk of all-cause dementia among people with BMI < 30 kg/m^2^ (HR [95% CI]: 0.74 [0.56–0.97]), whereas there was no association among those with BMI ≥ 30 kg/m^2^ (HR [95% CI]: 1.02 [0.92–1.12], and the estimates were significantly different (*p* = 0.029). The associations of GLP1 receptor agonist versus DPP4 inhibitor initiation with all-cause dementia risk were not statistically significant in those with atherosclerotic cardiovascular disease (HR [95% CI]: 0.89 [0.75–1.05]), and in those with renal insufficiency (HR [95% CI]: 0.87 [0.74–1.03]). The relative risk estimates for all-cause dementia with GLP1 receptor agonists versus DPP4 inhibitors did not significantly differ (*p* = 0.708) between those aged 60–70 (HR [95% CI]: 0.91 [0.81–1.03]) and > 70 years (HR [95% CI]: 0.95 [0.83–1.08]). With DPP4 inhibitor as a referent comparator, the estimates for different GLP1 receptor agonists numerically differed, but heterogeneity was not statistically significant (*p* = 0.641).

Reduction in all-cause dementia risk with SGLT2 inhibitors versus DPP4 inhibitors was specific to those aged > 70 years (HR [95% CI]: 0.87 [0.77–0.97]). A lower all-cause dementia risk associated with SGLT2 inhibitors was found among those with atherosclerotic cardiovascular disease (HR [95% CI]: 0.80 [0.68–0.95]) and renal insufficiency (HR [95% CI]: 0.80 [0.65–0.99]). Initiation of an SGLT2 inhibitor versus a DPP4 inhibitor was associated with lower dementia risk among people with BMI < 30 kg/m^2^ (HR [95% CI]: 0.82 [0.70–0.94]) but not among those with BMI ≥ 30 kg/m^2^ (HR [95% CI]: 0.97 [0.86–1.09]; heterogeneity: *p* = 0.072). The estimates were consistent across different SGLT2 inhibitors (*p* = 0.913) when DPP4 inhibitors were a referent comparator.

In the cohort of GLP1 receptor agonists versus SGLT2 inhibitors, the HR for all-cause dementia was 0.90 (95% CI: 0.75–1.07) among females, and the HR was 1.06 (95% CI: 0.91–1.24) among males; nonetheless, heterogeneity was not statistically significant (*p* = 0.163). GLP1 receptor agonist versus SGLT2 inhibitor initiation showed an HR of 0.90 (95% CI: 0.79–1.04) among those with BMI ≥ 30 kg/m^2^ and an HR of 1.07 (95% CI: 0.83–1.38) among those with BMI < 30 kg/m^2^, but the heterogeneity was not statistically significant (*p* = 0.241).

### Sensitivity analysis

The estimates from sensitivity analyses are summarized in Supplementary Figs. 5–7. The results across sensitivity analyses were consistent with those of the primary analysis.

The quantitative bias analysis suggested that moderate unmeasured confounding could potentially explain away an HR of 0.86 observed with SGLT2 inhibitors versus DPP4 inhibitors, whereas stronger unmeasured confounding is needed to explain away larger HRs observed in as-treated analyses (Supplementary Fig. 8 and 9).

## Discussion

In people aged ≥ 60 years with type 2 diabetes, SGLT2 inhibitor versus DPP4 inhibitor initiation was associated with lower risk of all-cause dementia, and the observed risk reduction was greater with continuous use. The intention-to-treat analysis showed no dementia risk difference between GLP1 receptor agonist and DPP4 inhibitor initiation; however, continuous use of GLP1 receptor agonists versus DPP4 inhibitors was associated with dementia risk reduction. Dementia risk was not significantly different between SGLT2 inhibitors and GLP1 receptor agonists in both intention-to-treat and as-treated analyses. Exploratory analyses suggested that SGLT2 inhibitors might reduce risks of both AD and VD, whereas associations between GLP1 receptor agonists and AD or VD were imprecise and inconclusive.

Concerns about important biases, such as selection bias, serious confounding by indication, detection bias, or reverse causality, limit the interpretation of some previous observational studies quantifying dementia risk associated with GLP1 receptor agonists (Supplementary Table 14 for further details). The present study emulated a target trial to avoid immortal time and selection bias, chose a second-line glucose-lowering therapy as an active comparator to reduce confounding by indication, used an outcome definition based only on primary care records to minimize detection bias due to differential hospitalizations, and implemented a lag time to mitigate bias due to reverse causality. This study formulated clear causal questions relevant to clinical decision-making by specifying a target trial emulation framework for pairwise comparisons of three common second-line therapies. Another notable strength is the ability to adjust for BMI, an important confounder that can introduce bias away from the null in this population [8, 9, 46]. Late-life obesity has been associated with a lower dementia risk, whereas mid-life obesity has been associated with a higher dementia risk [46].

Observational studies of people with diabetes [47, 48] that addressed immortal time bias [34] found no association between dementia risk and initiation of metformin, a first-line therapy for type 2 diabetes. A pilot trial suggested metformin might reduce cognitive decline in people without diabetes, and a large-scale dementia prevention trial with metformin has been proposed in people without diabetes [49–51]. The effect of metformin on cognition is currently inconclusive. In addition, according to observational and experimental data, DPP4 inhibitors [10, 11], sulfonylureas [11, 52], and insulin [53], are unlikely to slow cognitive decline beyond the benefits of glycemic control in diabetes without dementia. The GRADE trial suggested no difference in cognitive decline across insulin glargine, the DPP4 inhibitor sitagliptin, the sulfonylurea glimepiride, and the GLP1 receptor agonist liraglutide, but the mean age was 57.1 years, and SGLT2 inhibitors were not examined [54]. With DPP4 inhibitors as a comparator previously shown to have neutral effects on cognition [10, 11], the present study demonstrates that SGLT2 inhibitors may reduce dementia risk in adults with type 2 diabetes aged ≥ 60 years, but the association between the study GLP1 receptor agonists and dementia risk reduction is less certain. Further, the present study suggests no meaningful difference in dementia risk between SGLT2 inhibitors and GLP1 receptor agonists in this population.

The present study replicates the previously observed dementia risk reduction with SGLT2 inhibitor versus DPP4 inhibitor initiation in people with diabetes [12–15], but limited studies with adequate events have explored the association between continuous use of SGLT2 inhibitors and dementia aetiologies [13]. Continuous SGLT2 inhibitor versus DPP4 inhibitor use was also associated with lower risks of both AD and VD, strengthening previous evidence [13]. The findings inform opportunities for randomized trials to study SGLT2 inhibitors as potential treatments to mitigate dementia risk in an older population. Furthermore, SGLT2 inhibitors are recommended for those with atherosclerotic cardiovascular disease and chronic kidney disease due to cardiorenal protective properties [8, 9]. Neuroprotection may be an additional benefit among initiators of SGLT2 inhibitors with atherosclerotic cardiovascular disease and renal insufficiency, in which the absolute rates of dementia were higher. It is also notable that SGLT2 inhibitor versus DPP4 inhibitor initiation was associated with a lower dementia risk among those with BMI < 30 kg/m^2^, a subgroup containing some who are at a higher dementia risk in later life.

In contrast, the present study found no association in the intention-to-treat analysis but found dementia risk reduction with GLP1 receptor agonists in as-treated analyses. One possible explanation might be insufficient GLP1 receptor agonist exposure in many initiators. Based on a sensitivity analysis with a maximum 5-year follow-up time, exposure misclassification introduced by long follow-up times might explain only partly this null association. Discontinuation might be a factor limiting neuroprotective benefits of GLP1 receptor agonists. It should also be noted that the association seen in the as-treated analysis could be explained by time-varying confounding caused by informative censoring.

Intention-to-treat analyses from rigorous cohort studies with sufficient dementia events showed mixed findings when GLP1 receptor agonists were compared with DPP4 inhibitors [23, 24]. Particularly, one study concluded significantly different relative risk estimates between people aged ≥ 75 versus 66–75 years (1.22 versus 0.64) [24], and the other study concluded comparable relative risk estimates between people aged ≥ 75 versus 65–75 years (0.83 versus 0.76) [23]. In our study, the estimates did not significantly differ between age subgroups. Moreover, GLP1 receptor agonist versus DPP4 inhibitor initiation showed greater relative reduction in dementia risk among those with < 30 kg/m^2^ than among those with ≥ 30 kg/m^2^. The effect of GLP1 receptor agonists on dementia risk might be highly heterogeneous in routine clinical practice, implying the need to identify individuals who are more likely to benefit from their neuroprotective effects. Further, the estimates across GLP1 receptor agonist molecules in our study were numerically but not statistically heterogeneous, and initiators of semaglutide, a newer GLP1 receptor agonist, were followed for a relatively short time in this study. A rigorously designed study reported an imprecise estimate favouring semaglutide [55], but events in that study were scarce, highlighting a need for further investigation as data continue to accumulate. Dementia risk associated with semaglutide, as well as the newer tirzepatide, remains to be established in rigorous studies with large samples and long follow-up.

Prior reports concluded small or no dementia risk difference between initiation of GLP1 receptor agonists versus SGLT2 inhibitors [16, 25, 26], consistent with our study. Kaplan–Meier curves representing our as-treated analysis of GLP1 receptor agonists versus SGLT2 inhibitors implied that long-term continuous SGLT2 inhibitor use might be associated with greater benefit. In agreement, the Kaplan–Meier curves from two prior intention-to-treat analyses also showed less benefit of GLP1 receptor agonists versus SGLT2 inhibitors after several years of follow-up [16, 25]. These observations indicate the importance of additional investigations of long-term dementia risk.

Among initiators of GLP1 receptor agonists versus SGLT2 inhibitors, the direction of effect modification by age in two prior studies [16, 25] was conflicting, and the direction of effect modification by cardiovascular disease and sex in our study disputes prior evidence [16, 25]. Future studies whenever feasible might continue to explore heterogeneity of treatment effects of GLP1 receptor agonists or SGLT2 inhibitors, especially in clinically relevant subgroups, across BMI strata, and across age strata, which may suggest opportunities to optimize personalized dementia risk mitigation based on risk profiles.

### Limitations

First, residual confounding is possible in observational studies due to unmeasured confounders such as frailty, neuropsychiatric measures (e.g. Mini-Mental State Examination), socioeconomic status, education, and family history of dementia. These unmeasured confounders could account for the observed benefit to some extent, and the results should be cautiously interpreted. Randomized controlled trials are needed to confirm the observational evidence. Second, time-varying confounding due to informative censoring could be present in as-treated analyses, while an intention-to-treat analysis is not susceptible to treatment-related informative censoring. Third, dementia diagnoses are usually made in a secondary care setting, so dementia incidence in primary care could have been delayed or missed. The relative and absolute measures could have been underestimated by non-differential outcome misclassification. Fourth, the analyses with dementia subtypes should be considered exploratory and interpreted with caution, because the diagnoses were not necessarily biomarker-confirmed, and the referent standard in the validation studies [38, 39] was not biomarker-confirmed diagnoses. Fifth, BMI, as well as eGFR, was ascertained based on the latest available measure during the 1-year lookback window; therefore, the measures in some people might be less accurate measures due to missing data at the time of drug initiation. Inaccuracy in subgroup classification might dilute the observed magnitude of effect modification. Sixth, because the exposure was based on prescription records, it is unknown whether the prescriptions were dispensed, which might contribute to observed effect estimates smaller here than those in studies that used dispensing data.

## Conclusion

In people aged ≥ 60 years with type 2 diabetes, SGLT2 inhibitor initiation was associated with reduced dementia risk. Dementia risk reduction associated with injectable GLP1 receptor agonists was observed only with continuous use. No significant difference in dementia risk was observed between SGLT2 inhibitors versus GLP1 receptor agonists. However, as unmeasured confounding was possible, and the as-treated analyses were prone to time-varying confounding due to informative censoring, the estimates should be cautiously interpreted.

## Supplementary Information

Supplementary Material 1.

## Data Availability

This study is based in part on data from the Clinical Practice Research Datalink obtained under licence from the UK Medicines and Healthcare products Regulatory Agency. The data is provided by patients and collected by the NHS as part of their care and support. The interpretation and conclusions contained in this study are those of the author/s alone. The access to the primary care and linked datasets requires approval but can be requested via applications to the Clinical Practice Research Datalink Research Data (https://www.cprd.com). Statistical codes are available at: (https://gitea.com/joeywu/CPRD_2545).

## Abbreviations

SGLT2: Sodium-glucose cotransporter 2
GLP1: Glucagon-like peptide-1
DPP4: Dipeptidyl peptidase-4
CARMELINA: The CArdiovascular and Renal Microvascular outcomE study with LINAgliptin
CAROLINA: CARdiovascular Outcome study of LINAgliptin versus glimepiride in patients with type 2 diabetes
BMI: Body mass index
eGFR: Estimated glomerular filtration rate
HbA1c: Hemoglobin A1c
VD: Vascular dementia
AD: Alzheimer’s disease
HR: Hazard ratio
IRD: Incidence rate difference
CI: Confidence interval
PPV: Positive predictive value
CPRD: Clinical Practice Research Datalink
ONS: Office for National Statistics
HES APC: Hospital Episode Statistics Admitted Patient Care
OPCS: The UK Office of Population, Census and Surveys classification
ICD-10: International Statistical Classification of Diseases version 10
