# Supplementary material for: Comparative dementia risk with GLP1 receptor agonists, SGLT2 inhibitors, or DPP4 inhibitors: a population-based cohort study
Source: Alzheimers Res Ther. 2025 Dec 20;17:269. doi: 10.1186/s13195-025-01929-x (PMC12751920; doi:10.1186/s13195-025-01929-x)
Supplement: Supplementary file 1 — Supplementary Material 1. [file 13195_2025_1929_MOESM1_ESM.pdf]

Supplementary Material

Supplementary Table 1. Target Trial Emulation Framework. 3

Supplementary Table 2. SNOMED-CT Classification Codes for Cognitive Deficits in CPRD Aurum. 5

Supplementary Table 3. READ Codes for Cognitive Deficits in CPRD GOLD. 8

Supplementary Table 4. SNOMED-CT Classification Codes for Dementia in CPRD Aurum. 11

Supplementary Table 5. READ Codes for Dementia in CPRD GOLD. 17

Supplementary Table 6. ICD Codes Used to Exclude Dementia and Cognitive Deficits. 19

Supplementary Table 7. Covariate Ascertainment. 20

Supplementary Table 8. Breakdown of Molecules. 24

Supplementary Table 9. Full Baseline Characteristics of Initiators of GLP1 receptor agonists versus DPP4 Inhibitors. 25

Supplementary Table 10. Full Baseline Characteristics of Initiators of SGLT2 Inhibitors versus DPP4 Inhibitors. 29

Supplementary Table 11. Full Baseline Characteristics of Initiators of GLP1 receptor agonists versus SGLT2 Inhibitors. 33

Supplementary Table 12. Dementia Risks Associated with SGLT2 Inhibitors versus DPP4 Inhibitors, GLP1 receptor agonists versus DPP4 Inhibitors, and GLP1 Receptor Agonists versus SGLT2 Inhibitors Before Propensity Score Weighting. 37

Supplementary Table 13. Summary of Endpoints. 39

Supplementary Table 14. Summary of Cohort Studies of Dementia Risk with GLP1 Receptor Agonists. 41

Supplementary Figure 1. Cohort Entry Flowchart. 44

Supplementary Figure 2. Propensity Score Distribution of GLP1 Receptor Agonists versus DPP4 Inhibitors. 45

Supplementary Figure 3. Propensity Score Distribution of SGLT2 Inhibitors versus DPP4 Inhibitors. 46

Supplementary Figure 4. Propensity Score Distribution of GLP1 Receptor Agonists versus SGLT2 Inhibitors. ___ ***47***

Supplementary Figure 5. Sensitivity Analyses of GLP1 Receptor Agonists versus DPP4 Inhibitors. ___ ***48***

Supplementary Figure 6. Sensitivity Analyses of SGLT2 Inhibitors versus DPP4 Inhibitors. ___ ***49***

Supplementary Figure 7. Sensitivity Analyses of GLP1 Receptor Agonists versus SGLT2 Inhibitors. ___ ***50***

Supplementary Figure 8. Quantitative Bias Analysis for GLP1 Receptor Agonists versus DPP4 Inhibitors. ___ ***51***

Supplementary Figure 9. Quantitative Bias Analysis for SGLT2 Inhibitors versus DPP4 Inhibitors. ___ ***52***

Supplementary References ___ ***53***

Supplementary Table 1. Target Trial Emulation Framework.

	Hypothetical Target Trial	Trial Emulation Cohort
**Eligibility**	Inclusion:<br>1. Age ≥60 years<br>2. A diagnosis of type 2 diabetes<br><br>Exclusion:<br>1. Prevalent cognitive impairment<br>2. Type 1 diabetes<br>3. History of thyroid cancer or multiple endocrine neoplasia<br>4. A history end-stage kidney disease, dialysis in the past year, or latest eGFR <20 mL/min/1.73m² in the past year<br>5. Prescription of the study drug classes in the past year	Same as the target trial
**Treatment Strategies**	***First trial***:<br>1. GLP1 receptor agonists (injectable)<br>2. DPP4 inhibitors<br><br>***Second trial:***<br>1. SGLT2 inhibitors<br>2. DPP4 inhibitors<br><br>***Third trial***<br>1. GLP1 receptor agonists (injectable)<br>2. SGLT2 inhibitors	Same as the target trial
**Treatment Assignment**	Open-label, two-arm, parallel, randomized trials.	Propensity score weighting created an observational analogue of randomization.
**Outcome**	***Primary Outcome:*** All-cause dementia<br>***Secondary Outcomes:*** Alzheimer's disease, Vascular dementia	Same as the target trial. However, unlike the target trials, the outcomes were defined based on primary care records
**Follow-up**	Follow-up started from randomization. Follow-up ended at death, loss to follow-up, or incident dementia.	Follow-up started from treatment initiation. Follow-up ended at death, end of registration, or the event of interest.
**Causal Contrast**	Intention-to-treat analysis.	***Primary:*** Modified intention-to-treat analysis.<br><br>The causal contrast here was modified intention-to-treat, rather than an intention-to-treat effect, because a 1-year lag time was used to mitigate reverse causality (i.e., only eligible individuals with follow-up of at least 1 year and without dementia during the first year of follow-up from treatment initiation were analyzed).<br><br>***Secondary:*** Observational analogue of as-treated analysis.

		In the as-treated analysis, follow-up ended at the endpoints described in the follow-up section or 1 year after treatment discontinuation or crossover. The 1-year extended observation window attempted to mitigate informative censoring due to delayed diagnosis.
**Statistical Analysis**	A Cox proportional hazard model was used to compare time to incident dementia between treatment groups among eligible individuals randomized into the treatment arms.	***Primary:*** A Cox proportional hazard model with the matching weights was used to compare time to incident dementia between groups among eligible individuals. The at-risk time started from the end of the 1-year lag time to mitigate reverse causality. The at-risk time ended at the occurrence of endpoints described in the follow-up section.<br><br>***Secondary:*** A Cox proportional hazard model with the matching weights was used to compare time to incident dementia between groups among eligible individuals. The at-risk time started from the end of the 1-year lag time to mitigate reverse causality. The at-risk time ended at the occurrence of endpoints described in the follow-up section or 1 year after treatment discontinuation or crossover.

Abbreviations: GLP1 = glucagon-like peptide-1; SGLT2 = sodium-glucose cotransporter 2; DPP4 = dipeptidyl peptidase-4; eGFR = estimated glomerular filtration rate

Supplementary Table 2. SNOMED-CT Classification Codes for Cognitive Deficits in CPRD Aurum.

Medcodeid	Term	Originalreadcode
295525018	Mild memory disturbance	E2A10
295526017	Organic memory impairment	E2A11
497290018	Forgetful	28G
1222481019	Memory deficit	R00z0-1
1491795010	Cognitive impairment	28E3
1491798012	Cognitive decline	28E
2159230012	GDS level 2 - very mild cognitive decline	3AE1
2159231011	GDS level 3 - mild cognitive decline	3AE2
2534215016	Referral to memory clinic	8HTY
2548507014	Disturbance of memory for order of events	1S21
3510998011	Cognitive deficit in communication skills	^ESCT1170619
3511000016	Cognitive deficit in visuospatial function	^ESCT1170620
3636346015	Cognitive impairment due to toxicity	^ESCT1168969
215831000000117	Mild cognitive disorder	Eu057
293711000006110	Neurocognitive disorder	R140z
404241000006118	Impaired cognition	Ryu51
557671000000111	Seen in memory clinic	9Nk1
1947931000006119	Cognitive communication disorder	Eu058
1971711000006115	Mental & behav dis due to use opioids: resid & late-onset psychot dis, other persisting cognitive impairment	EMISICD10|F1174
1971721000006111	Mental & behav dis due to seds/hypntcs: resid & late-onset psychot dis, other persisting cognitive impairment	EMISICD10|F1374
1972441000006115	Mental & behav dis due to cannabinoids: resid & late-onset psychot dis, other persisting cognitive impairment	EMISICD10|F1274
1973181000006110	Mental and behav dis due to vol solvents: resid & late-onset psychotic dis, other persisting cognitive impairment	EMISICD10|F1874
1973741000006117	Mental and behav dis due to hallucinogens: resid & late-onset psychot dis, other persisting cognitive impairment	EMISICD10|F1674
1973971000006110	Mental & behav dis due to use cocaine: resid & late-onset psychot dis, other persisting cognitive impairment	EMISICD10|F1474
1974291000006118	Mental and behav dis mlti drg use/oth psych subs: resid/late psychot dis, other persisting cognitive impairment	EMISICD10|F1974
1974961000006117	Mental & behav dis due to tobacco: resid & late-onset psychot dis, other persisting cognitive impairment	EMISICD10|F1774
1976071000006119	Mental & behav dis due to use alcohol: resid & late-onset psychot dis, other persisting cognitive impairment	EMISICD10|F1074
1976161000006113	Mental and behav dis due to other stimulants inc caffeine: resid/late-onset psycht dis, other persisting cognitive impairment	EMISICD10|F1574
2288201000000116	Mild cognitive impairment	28E0
2288241000000118	Moderate cognitive impairment	28E1
2288281000000114	Severe cognitive impairment	28E2
3179841000006119	Loss of memory for recent events	^ESCTLO317984
3279341000006111	Memory loss	^ESCTME327934
3279351000006113	LOM - Loss of memory	^ESCTLO327935
3279381000006117	Loss of memory	^ESCTLO327938
4178371000006110	Minimal cognitive impairment	^ESCTMI417837
4935531000006113	Distortion of memory	^ESCTDI493553
4935541000006115	Minor memory lapses	^ESCTMI493554
4935551000006118	Memory lapses	^ESCTME493555
5023491000006114	Age-associated memory impairment	^ESCTAG502349

5246181000006117	Short term memory loss	^ESCTSH524618
5246191000006119	Forgets what was going to do	^ESCTFO524619
5246201000006116	Forgets what was going to say	^ESCTFO524620
5246211000006118	Forgets recent activities	^ESCTFO524621
5246221000006114	Forgets what has just done	^ESCTFO524622
5246231000006112	Forgets what has just said	^ESCTFO524623
5246261000006115	Forgets what has just heard	^ESCTFO524626
6462081000006114	Cognitive disturbance	^ESCTCO646208
6462091000006112	Cognitive dysfunction	^ESCTCO646209
6462111000006115	Cognitive deficit	^ESCTCO646211
6462141000006116	Memory dysfunction	^ESCTME646214
6462171000006112	Memory problem	^ESCTME646217
6462191000006113	Bad memory	^ESCTBA646219
6462201000006111	Disturbance of memory	^ESCTDI646220
6749711000006110	Global Deterioration Scale (GDS) level 4 - moderate cognitive decline	^ESCTGL674971
6749741000006114	Global Deterioration Scale (GDS) level 5 - moderately severe cognitive decline	^ESCTGL674974
6749771000006118	Global Deterioration Scale (GDS) level 6 - severe cognitive decline	^ESCTGL674977
6768901000006114	Memory loss care assessment	^ESCTME676890
7274731000006114	Cognitive disorder	^ESCTCO727473
8044311000006115	Memory disorder co-occurrent and due to organic brain damage	^ESCTME804431
8044721000006110	Cognitive changes due to organic disorder	^ESCTCO804472
12009301000006116	Hypotonia, speech impairment, severe cognitive delay syndrome	^ESCT1200930
13935251000006116	Amnestic mild cognitive disorder	^ESCT1393525
13935261000006119	aMCI - amnestic mild cognitive impairment	^ESCT1393526
14068581000006119	Memory: present month not known	^ESCT1406858
14068591000006116	Memory: important event not known	^ESCT1406859
30991010	Prosopagnosia	F481M
43172010	Visual agnosia	F481H
298188010	Oculomotor apraxia	F4835
317290013	Aphasia	R043
401826019	Word deafness	E2F30-1
432821000006113	[X]Word deafness	Eu802-4
317312019	Agnosia	R0465
317314018	Apraxia	R0467
1209242010	Neurapraxia	SJ7x0
2474732018	Verbal apraxia	R0468
2534255019	Oral apraxia	R0469
266831000006117	Oculomotor apraxia - Cogan type	F4835-1
2635451000006112	Agnosia for smell	^ESCTAG263545
2664171000006117	Amnestic aphasia	^ESCTAM266417
2733561000000110	Non-fluent aphasia	^ESCT1171968
2866811000006118	Global aphasia	^ESCTGL286681
2866841000006119	Expressive-receptive aphasia	^ESCTEX286684
2995971000006117	Gait apraxia	^ESCTGA299597
3203201000006113	Auditory agnosia	^ESCTAU320320
3373431000006115	Motor apraxia	^ESCTMO337343
3579701000006118	Sensory apraxia	^ESCTSE357970
3706641000006111	Apraxia of speech	^ESCTAP370664
3863831000006112	Topographical agnosia	^ESCTTO386383
4997631000006111	Mixed aphasia	^ESCTMI499763
4997701000006110	Expressive aphasia	^ESCTEX499770

4998301000006113	Classic apraxia	^ESCTCL499830
4998311000006111	Ideomotor apraxia	^ESCTID499831
5005681000006115	Progressive aphasia	^ESCTPR500568
5005711000006119	Progressive aphasia in Alzheimer's disease	^ESCTPR500571
5528421000006116	Sensory agnosia	^ESCTSE552842
5550711000006115	Frenchay aphasia screening test	^ESCTFR555071
7002431000006114	Apraxia of eyelid	^ESCTAP700243
7746341000006118	Logopenic progressive aphasia	^ESCTLO774634
7840661000006110	Primary progressive aphasia	^ESCTPR784066
8259471000006113	Comprehensive aphasia test	^ESCTCO825947
8259551000006119	Comprehensive aphasia test score	^ESCTCO825955
8458671000006119	Requires information in aphasia-accessible format	^ESCTRE845867
8458861000006110	Requires aphasia-friendly communication	^ESCTRE845886
12028311000006118	Optic ataxia, gaze apraxia, simultanagnosia syndrome	^ESCT1202831
146910015	Dysphasia training	8E24
317303016	Dysphasia	R0451
4997601000006115	Anterior dysphasia	^ESCTAN499760
4997621000006113	Mixed dysphasia	^ESCTMI499762
4997641000006118	Receptive dysphasia	^ESCTRE499764
4997691000006110	Expressive dysphasia	^ESCTEX499769
5005691000006117	Non-Alzheimer's progressive dysphasia	^ESCTNO500569
6160211000006114	Semantic dysphasia	^ESCTSE616021
7650931000006117	Acquired dysphasia	^ESCTAC765093
8188261000006116	Referral to dysphasia support service	^ESCTRE818826

Supplementary Table 3. READ Codes for Cognitive Deficits in CPRD GOLD.

MedCode	Readcode	Readterm
26434	Z7CEH13	Bad memory
50418	Z7CEH12	Memory deficit
39915	Z7CEH11	Memory dysfunction
12583	Z7CEH15	Poor memory
12057	Z7CEH14	Memory problem
6387	E2A1000	Mild memory disturbance
105538	Z7CE414	Memory disturbance
7674	28E..00	Cognitive decline
60263	3AE2.00	GDS level 3 - mild cognitive decline
9786	Z7CEC11	Loss of memory for recent events
60600	Z7CFG00	Forgets what was going to say
64892	Z7CEN11	Invents experiences to compensate for loss of memory
40821	1B1a.00	Poor auditory sequential memory
103453	1B1A100	Short-term memory loss
27788	1B1A000	Temporary loss of memory
22802	8HTY.00	Referral to memory clinic
47581	Z7CFO11	Long-term memory loss
10571	Z7CF811	Short-term memory loss
51724	Z7CEL00	Mild memory disturbance
53978	Z7CFO00	Poor long-term memory
47994	Z7CFM00	Forgets what has just heard
65696	Z7CEA13	Impairment of primary memory
32367	Z7CEA11	Impairment of working memory
7742	28G..00	Forgetful
53507	Z7CEK00	Minor memory lapses
110307	1S23.00	Memory impairment
11410	Z7CF800	Poor short-term memory
70895	Z7CFK00	Forgets what has just read
11936	Eu05700	[X]Mild cognitive disorder
39507	1B1Y.00	Poor visual sequential memory
1993	1B1A.00	Memory loss - amnesia
24952	Z7CE500	Forgetful
19073	Z7CEJ00	Memory lapses
10123	Z7CE611	Memory loss
68230	Z7CE612	Memory gone
19297	Z7CE615	Loss of memory
12805	Z7CE614	Memory loss - amnesia
12277	Z7CE616	LOM - Loss of memory
112378	Ryu5.00	[X]Symptoms/signs inv cognit, percept, emotion state & behav
67802	Z7CEC12	No memory for recent events
52939	Ryu5100	[X]Oth & unspec symptom/sign involv cognit funct/awareness
41366	Z7CFH00	Forgets recent activities
107402	28E2.00	Severe cognitive impairment
107282	28E0.00	Mild cognitive impairment
37191	Z7CEB12	Poor memory for remote events
51379	Z7CE400	Memory disturbance (& amnesia (& symptom))
47882	Z7CEG00	Transient memory loss
93319	Z7CFJ00	Forgets what has just said
2908	1B1A.13	Memory disturbance
5777	1B1A.12	Memory loss symptom
40091	Z7CFI00	Forgets what has just done
110729	Eu05800	[X]Cognitive communication disorder
103479	Z7CFL00	Forgets what has just seen
67998	Z7CEF00	Temporary loss of memory

108266	28E3.00	Cognitive impairment
107482	28E1.00	Moderate cognitive impairment
102880	Z7CE415	Loss of memory
103375	Z7CE413	Memory loss - amnesia
67838	Z7CE412	Memory loss symptom
6061	E2A1100	Organic memory impairment
59515	Z7CFF00	Forgets what was going to do
51739	Z7CEM00	Distortion of memory
10514	Z7CEH00	Memory impairment
94164	9Nk1.00	Seen in memory clinic
39537	Z7CE300	Recovery of memory
93486	ZS78B11	Conduction aphasia
11598	ZS78.11	Aphasia
113830	Eu80214	[X]Word deafness
64865	E2F3011	Word deafness
70852	Z7CM511	Word deafness
70527	Z7CM500	Auditory agnosia
96797	Z7CMC00	Topographical agnosia
91135	ZS78I11	Non-fluent aphasia
28872	ZS78G11	Expressive aphasia
61575	Z7CN300	Finger agnosia
47000	Z7CMD00	Agnosia for smell
112366	ZS78211	Isolation aphasia
96876	ZR11.00	Aachen aphasia test
68774	ZS78911	Mixed aphasia
113917	ZS78A11	Semantic aphasia
101320	ZS78D11	Jargon aphasia
51158	ZS78D13	Wernicke's aphasia
104523	Z7CM.00	Sensory agnosia
92389	Z7CM300	Agnosia for pain
92306	Z7CMB00	Visuospatial agnosia
37771	ZS78511	Anomic aphasia
67588	ZS78K11	Efferent motor aphasia
36667	Z7E5100	Apraxia
56334	Z7CM400	Agnosia for temperature
93178	ZS78411	Transcortical motor aphasia
95891	Z7CM900	Visual agnosia for objects
57542	ZS78C11	Receptive aphasia
35016	F481H00	Visual agnosia
20214	R046700	[D]Apraxia
46332	Z7CM800	Prosopagnosia
98539	ZS78300	Subcortical aphasia
47008	ZS51.00	Apraxia of speech
101745	R046800	[D]Verbal apraxia
63425	ZS78811	Global aphasia
107256	F481M00	Prosopagnosia
105929	ZS78611	Transcortical sensory aphasia
98834	ZS52.11	Oral apraxia
110267	Z7CM700	Visual agnosia
67135	ZS78E11	Fluent aphasia
54017	ZS78H11	Broca's aphasia
93809	R046900	[D]Oral apraxia
54274	F483511	Oculomotor apraxia - Cogan type
72636	Z7E5200	Gestural apraxia
15651	R043.00	[D]Aphasia
104383	ZS53.11	Verbal apraxia

72081	Z7CMA00	Simultagnosia
34751	F483500	Oculomotor apraxia
25824	SJ7x000	Neurapraxia
56701	Z7CME00	Agnosia for taste
20131	R046500	[D]Agnosia
52686	ZS78900	Mixed dysphasia
89004	ZS78A00	Semantic dysphasia
95596	ZS78212	Isolation dysphasia
71374	ZS78K00	Efferent motor dysphasia
42830	8E24.00	Dysphasia training
113777	ZS78B00	Conduction dysphasia
48292	ZS78100	Acquired dysphasias
9992	ZS78.00	Dysphasia
3537	R045100	[D]Dysphasia
66978	ZS78D12	Jargon dysphasia
49609	ZS78C00	Receptive dysphasia
64964	ZS78200	Mixed transcortical dysphasia
101822	ZS78600	Transcortical sensory dysphasia
112862	ZS78400	Transcortical motor dysphasia
48919	ZS78H00	Broca's dysphasia
98050	ZS78F00	Posterior dysphasia
107238	ZS78700	Frontal dynamic dysphasia
65074	ZS78D00	Wernicke's dysphasia
50803	ZS78I00	Non-fluent dysphasia
50602	ZS78800	Global dysphasia
10110	ZS78G00	Expressive dysphasia
35192	ZS78513	Nominal dysphasia
56551	ZS78512	Anomic dysphasia
64191	ZS78E00	Fluent dysphasia

Supplementary Table 4. SNOMED-CT Classification Codes for Dementia in CPRD Aurum.

Medcodeid	Term	Originalreadcode
**Alzheimer's Disease**		
1971401000006111	dementia in alzheimer's disease with early onset, without additional symptoms	EMISICD10|F0000
1971541000006114	dementia in alzheimer's disease with early onset, other symptoms, predominantly delusional	EMISICD10|F0001
1971771000006112	dementia in alzheimer's disease with early onset, other symptoms, predominantly hallucinatory	EMISICD10|F0002
1972131000006115	dementia in alzheimer's disease with early onset, other symptoms, predominantly depressive	EMISICD10|F0003
1972141000006113	dementia in alzheimer's disease with early onset, other mixed symptoms	EMISICD10|F0004
1972171000006117	dementia in alzheimer's disease with late onset, without additional symptoms	EMISICD10|F0010
1972181000006119	dementia in alzheimer's disease with late onset, other symptoms, predominantly delusional	EMISICD10|F0011
1972191000006116	dementia in alzheimer's disease with late onset, other symptoms, predominantly hallucinatory	EMISICD10|F0012
1972201000006118	dementia in alzheimer's disease with late onset, other symptoms, predominantly depressive	EMISICD10|F0013
1972211000006115	dementia in alzheimer's disease with late onset, other mixed symptoms	EMISICD10|F0014
1972231000006114	dementia in alzheimer's dis, atypical or mixed type, without additional symptoms	EMISICD10|F0020
1972251000006119	dementia in alzheimer's dis, atypical or mixed type, other symptoms, predominantly delusional	EMISICD10|F0021
1972291000006113	dementia in alzheimer's dis, atypical or mixed type, other symptoms, predominantly hallucinatory	EMISICD10|F0022
1972311000006112	dementia in alzheimer's dis, atypical or mixed type, other symptoms, predominantly depressive	EMISICD10|F0023
1972341000006111	dementia in alzheimer's dis, atypical or mixed type, other mixed symptoms	EMISICD10|F0024
1972371000006115	dementia in alzheimer's disease, unspecified, without additional symptoms	EMISICD10|F0090
1972401000006117	dementia in alzheimer's disease, unspecified, other symptoms, predominantly delusional	EMISICD10|F0091
1972451000006118	dementia in alzheimer's disease, unspecified, other symptoms, predominantly depressive	EMISICD10|F0093
1972471000006111	dementia in alzheimer's disease, unspecified, other mixed symptoms	EMISICD10|F0094
26545010	senile dementia	E00-1
293123100006118	ad - alzheimer's disease	^ESCTAD293123
293124100006111	alzheimer disease	^ESCTAL293124
293125100006113	alzheimer dementia	^ESCTAL293125
294635013	uncomplicated senile dementia	E000
294637017	uncomplicated presenile dementia	E0010
294644014	senile dementia with depressive or paranoid features	E002
294645010	senile dementia with paranoia	E0020
294646011	senile dementia with depression	E0021
294647019	senile dementia with depressive or paranoid features nos	E002z
294648012	senile dementia with delirium	E003
295668011	[x]dementia in alzheimer's disease	Eu00
295671015	[x]dementia in alzheimer's dis, atypical or mixed type	Eu002
295672010	[x]dementia in alzheimer's disease, unspecified	Eu00z
299325013	[x]other alzheimer's disease	Fyu30
359141000006111	[x] senile dementia nos	Eu02z-4
359151000006113	[x] senile dementia, depressed or paranoid type	Eu02z-6
359161000006110	[x] senile psychosis nos	Eu02z-5
363021000006113	[x]alzheimer's dementia unspec	Eu00z-1
363031000006111	[x]alzheimer's disease type 1	Eu001-1

363041000006118	[x]alzheimer's disease type 2	Eu000-3
376531000006119	[x]dementia in alzheimer's disease with early onset	Eu000
376541000006112	[x]dementia in alzheimer's disease with late onset	Eu001
423351000006115	[x]presenile dementia,alzheimer's type	Eu000-1
423381000006111	[x]primary degen dementia of alzheimer's type, senile onset	Eu001-3
423391000006114	[x]primary degen dementia, alzheimer's type, presenile onset	Eu000-2
425901000006116	[x]senile dementia, alzheimer's type	Eu001-2
45046017	alzheimer's disease	F110
499946014	alzheimer's disease with early onset	F1100
500317011	alzheimer's disease with late onset	F1101
6897211000006117	primary degenerative dementia of the alzheimer type, presenile onset	^ESCTPR689721
6897241000006118	dementia of the alzheimers type with early onset	^ESCTDE689724
6897251000006116	presenile dementia, alzheimer's type	^ESCTPR689725
6897271000006114	dementia in alzheimer's disease - type 2	^ESCTDE689727
6900181000006114	primary degenerative dementia of the alzheimer type, senile onset	^ESCTPR690018
6900201000006110	dementia of the alzheimers type, late onset	^ESCTDE690020
6900221000006117	sdat - senile dementia, alzheimer's type	^ESCTSD690022
6900241000006112	dementia in alzheimer's disease - type 1	^ESCTDE690024
882201000006116	senile dementia - simple type	E000-99
882211000006118	senile dementia-acute confused	E003-99
905791000006115	[rfc] alzheimer's disease	HNG0062
914951000006114	[d] dementia in alzheimer's disease	EMISNQDD3
15035271000006110	Alzheimer disease with psychosis	^ESCT1503527
8044981000006111	Alzheimer's disease co-occurrent with delirium	^ESCTAL804498
8044951000006115	Alzheimers dementia with depressed mood	^ESCTAL804495
3392901000006118	Alzheimers dementia, late onset, with delusions	^ESCTAL339290
5005541000006113	Familial Alzheimer's disease of late onset	^ESCTFA500554
5005501000006111	Familial Alzheimer's disease of early onset	^ESCTFA500550
14464361000006117	Autosomal dominant Alzheimer disease due to mutation of presenilin 1	^ESCT1446436
7834071000006116	Non-amnestic Alzheimer disease	^ESCTNO783407
5005521000006118	Non-familial Alzheimer's disease of early onset	^ESCTNO500552
5005561000006112	Non-familial Alzheimer's disease of late onset	^ESCTNO500556
7515131000006110	Primary degenerative dementia of the Alzheimer type, presenile onset in remission	^ESCTPR751513
2603271000006110	Primary degenerative dementia of the Alzheimer type, presenile onset, uncomplicated	^ESCTPR260327
3558401000006117	Primary degenerative dementia of the Alzheimer type, presenile onset, with delirium	^ESCTPR355840
3384371000006119	Primary degenerative dementia of the Alzheimer type, presenile onset, with delusions	^ESCTPR338437
2667431000006115	Primary degenerative dementia of the Alzheimer type, presenile onset, with depression	^ESCTPR266743
7515121000006112	Primary degenerative dementia of the Alzheimer type, senile onset in remission	^ESCTPR751512
3575041000006117	Primary degenerative dementia of the Alzheimer type, senile onset, uncomplicated	^ESCTPR357504
2575731000006115	Primary degenerative dementia of the Alzheimer type, senile onset, with delirium	^ESCTPR257573
3392881000006115	Primary degenerative dementia of the Alzheimer type, senile onset, with delusions	^ESCTPR339288
2929931000006112	Primary degenerative dementia of the Alzheimer type, senile onset, with depression	^ESCTPR292993
15215821000006112	Frontal variant non-amnestic Alzheimer disease	^ESCT1521582
9896131000006110	Behavioural disturbance co-occurrent and due to late onset Alzheimer dementia	^ESCTBE989613
8028391000006119	Anti-dementia drug therapy	^ESCTAN802839
8030791000006119	Altered behaviour in Alzheimer's disease	^ESCTAL803079
8030801000006118	Altered behavior in Alzheimer's disease	^ESCTAL803080
8044911000006116	Delusions in Alzheimer's disease	^ESCTDE804491
8044941000006117	Depressed mood in Alzheimer's disease	^ESCTDE804494
12370651000006112	MVAD - Mixed vascular Alzheimer dementia	^ESCT1237065

**Vascular Dementia**		
12370651000006112	MVAD - Mixed vascular Alzheimer dementia	^ESCT1237065
149347010	binswanger's disease	F21y2
1972481000006114	vascular dementia of acute onset, without additional symptoms	EMISICD10|F0100
1972501000006116	vascular dementia of acute onset, other symptoms, predominantly delusional	EMISICD10|F0101
1972571000006110	vascular dementia of acute onset, other mixed symptoms	EMISICD10|F0104
1972661000006119	multi-infarct dementia, other symptoms, predominantly depressive	EMISICD10|F0113
1972681000006112	multi-infarct dementia, other mixed symptoms	EMISICD10|F0114
1972711000006113	subcortical vascular dementia, without additional symptoms	EMISICD10|F0120
1972731000006119	subcortical vascular dementia, other symptoms, predominantly delusional	EMISICD10|F0121
1972751000006114	subcortical vascular dementia, other symptoms, predominantly hallucinatory	EMISICD10|F0122
1972771000006116	subcortical vascular dementia, other symptoms, predominantly depressive	EMISICD10|F0123
1972791000006115	subcortical vascular dementia, other mixed symptoms	EMISICD10|F0124
1972821000006112	mixed cortical and subcortical vascular dementia, without additional symptoms	EMISICD10|F0130
1972831000006110	mixed cortical and subcortical vascular dementia, other symptoms, predominantly delusional	EMISICD10|F0131
1972871000006113	mixed cortical and subcortical vascular dementia, other symptoms, predominantly hallucinatory	EMISICD10|F0132
1972911000006111	mixed cortical and subcortical vascular dementia, other symptoms, predominantly depressive	EMISICD10|F0133
1972931000006117	mixed cortical and subcortical vascular dementia, other mixed symptoms	EMISICD10|F0134
1973221000006118	other vascular dementia, without additional symptoms	EMISICD10|F0180
1973271000006117	other vascular dementia, other symptoms, predominantly delusional	EMISICD10|F0181
1973341000006118	other vascular dementia, other symptoms, predominantly hallucinatory	EMISICD10|F0182
1973381000006112	other vascular dementia, other symptoms, predominantly depressive	EMISICD10|F0183
1973401000006112	other vascular dementia, other mixed symptoms	EMISICD10|F0184
1973461000006113	vascular dementia, unspecified, without additional symptoms	EMISICD10|F0190
1973531000006117	vascular dementia, unspecified, other symptoms, predominantly hallucinatory	EMISICD10|F0192
1973551000006112	vascular dementia, unspecified, other symptoms, predominantly depressive	EMISICD10|F0193
1976831000006111	vascular dementia, unspecified, other mixed symptoms	EMISICD10|F0194
13934381000006118	Cortical vascular dementia	^ESCT1393438
294652012	uncomplicated arteriosclerotic dementia	E0040
294653019	arteriosclerotic dementia with delirium	E0041
294654013	arteriosclerotic dementia with paranoia	E0042
294655014	arteriosclerotic dementia with depression	E0043
294656010	arteriosclerotic dementia nos	E004z
295680015	[x]other vascular dementia	Eu01y
295681016	[x]vascular dementia, unspecified	Eu01z
3414231000006117	mid - multi-infarct dementia	^ESCTMI341423
3414251000006112	vad - vascular dementia	^ESCTVA341425
3414261000006114	multi infarct dementia	^ESCTMU341426
363791000006112	[x]arteriosclerotic dementia	Eu01-1
3964661000006114	subcortical atherosclerotic dementia	^ESCTSU396466
398571000006112	[x]mixed cortical and subcortical vascular dementia	Eu013
399031000006111	[x]multi-infarct dementia	Eu011
428201000006119	[x]subcortical vascular dementia	Eu012
431681000006117	[x]vascular dementia	Eu01
431691000006119	[x]vascular dementia of acute onset	Eu010
497559016	arteriosclerotic dementia	E004
696161000006115	multi infarct dementia	E004-1
914921000006117	[d] vascular dementia	EMISNQDV1
7840631000006118	Ischaemic vascular dementia	^ESCTIS784063
8024201000006111	Mixed dementia	^ESCTMI802420

8033241000006113	Multi-infarct dementia due to atherosclerosis	^ESCTMU803324
2664601000006115	Multi-infarct dementia with delirium	^ESCTMU266460
2912321000006113	Multi-infarct dementia with delusions	^ESCTMU291232
2722931000006114	Multi-infarct dementia with depression	^ESCTMU272293
3653671000006113	Multi-infarct dementia, uncomplicated	^ESCTMU365367
2729791000000114	Predominantly cortical vascular dementia	^ESCT1171774
7515061000006117	Vascular dementia in remission	^ESCTVA751506
2912331000006111	Vascular dementia, with delusions	^ESCTVA291233
2664611000006117	Vascular dementia, with delirium	^ESCTVA266461
14805251000006118	Behavioural disturbance due to multi-infarct dementia	^ESCT1480525
**Others**		
7510741000006111	Dementia associated with multiple sclerosis	^ESCTDE751074
7510731000006118	Dementia associated with normal pressure hydrocephalus	^ESCTDE751073
7511971000006114	Dementia associated with viral encephalitis	^ESCTDE751197
2502971000006115	dementia associated with alcoholism	^ESCTDE250297
2502981000006117	alcohol-induced persisting dementia	^ESCTAL250298
2707661000006119	pick disease	^ESCTPI270766
294688019	drug-induced dementia	E02y1
295686014	[x]dementia in creutzfeldt-jakob disease	Eu021
295687017	[x]dementia in huntington's disease	Eu022
295688010	[x]dementia in parkinson's disease	Eu023
299641000000112	[x]lewy body dementia	Eu025
5005421000006117	Amyotrophic lateral sclerosis with dementia	^ESCTAM500542
346929012	alcoholic dementia nos	E012-1
362941000006113	[x]alcoholic dementia nos	Eu107-1
376571000006116	[x]dementia in human immunodef virus [hiv] disease	Eu024
3802621000006119	lewy body variant of alzheimer's disease	^ESCTLE380262
3802651000006111	dementia of the lewy body type	^ESCTDE380265
401760017	other alcoholic dementia	E012
6973421000006116	dementia associated with aids	^ESCTDE697342
6973461000006110	acquired immune deficiency syndrome dementia complex	^ESCTAC697346
7043651000006119	dementia associated with parkinson's disease	^ESCTDE704365
7043661000006117	dementia associated with parkinson disease	^ESCTDE704366
7560531000006110	Frontotemporal dementia with parkinsonism-17	^ESCTFR756053
8031751000006119	Dementia due to Parkinson's disease	^ESCTDE803175
8031761000006117	Dementia due to Parkinsons disease	^ESCTDE803176
8031771000006112	Dementia due to Parkinson disease	^ESCTDE803177
7263021000006116	dementia due to huntingtons disease	^ESCTDE726302
8009521000006114	dementia due to picks disease	^ESCTDE800952
8009531000006112	dementia due to pick disease	^ESCTDE800953
914931000006119	[d] dementia with lewy bodies	EMISNQDD1
3636474012	Dementia due to and following injury of head	^ESCT1169049
7755121000006115	Behavioural variant of frontotemporal dementia	^ESCTBE775512
5005601000006112	Frontotemporal dementia	^ESCTFR500560
7750421000006118	Right temporal atrophy variant frontotemporal dementia	^ESCTRI775042
7511421000006118	Post-traumatic dementia with behavioural change	^ESCTPO751142
8042791000006116	Lewy body dementia with behavioural disturbance	^ESCTLE804279
15035231000006112	Amyotrophic lateral sclerosis with frontotemporal dementia	^ESCT1503523
7813831000006118	Familial Alzheimer-like prion disease	^ESCTFA781383
6024071000006116	Lewy body dementia	^ESCTLE602407
8042801000006115	Lewy body dementia with behavioral disturbance	^ESCTLE804280
14669181000006111	Presenile dementia with AIDS (acquired immunodeficiency syndrome)	^ESCT1466918
6024061000006111	Senile dementia of the Lewy body type	^ESCTSE602406
7755131000006117	Behavioral variant of frontotemporal dementia	^ESCTBE775513
8024641000006111	Altered behaviour in Huntington's dementia	^ESCTAL802464
**Unspecified Dementia**		
148381000006115	senile/presenile dementia	E00-2
1823871000006112	dementia confirmed	EMISNQDE35
1972021000006119	unspecified dementia, without additional symptoms	EMISICD10|F03X0
1972041000006114	unspecified dementia, other symptoms, predominantly delusional	EMISICD10|F03X1
1972061000006113	unspecified dementia, other symptoms, predominantly hallucinatory	EMISICD10|F03X2

1972071000006118	unspecified dementia, other symptoms, predominantly depressive	EMISICD10|F03X3
1972081000006115	unspecified dementia, other mixed symptoms	EMISICD10|F03X4
21256010	presenile dementia	E001
294638010	presenile dementia with delirium	E0011
294641018	presenile dementia with paranoia	E0012
294642013	presenile dementia with depression	E0013
294643015	presenile dementia nos	E001z
294718018	dementia in conditions ec	E041
295684012	[x]dementia in other diseases classified elsewhere	Eu02
295690011	[x]dementia in other specified diseases classif elsewhere	Eu02y
295714013	[x]delirium superimposed on dementia	Eu041
7874771000006117	Delirium superimposed on dementia	^ESCTDE787477
359081000006118	[x] presenile dementia nos	Eu02z-1
359101000006114	[x] primary degenerative dementia nos	Eu02z-3
359241000006119	[x] unspecified dementia	Eu02z
423221000006117	[x]predominantly cortical dementia	Eu011-1
882171000006115	dementia	E00-97
882181000006117	other senile/presenile dement.	E00-98
882191000006119	senile and presenile dementias	E00-99
914941000006112	[d] dementia	EMISNQDD2
939491000006118	[rfc] dementia	HNGNQRF130
359091000006115	[X] Presenile psychosis NOS	Eu02z-2
346897015	Presbyophrenic psychosis	E00y-1
7874761000006112	Delirium co-occurrent with dementia	^ESCTDE787476
8014401000006116	Early onset dementia with delusions	^ESCTEA801440
8014391000006118	Presenile dementia with delusions	^ESCTPR801439
8010111000006117	Primary degenerative dementia	^ESCTPR801011
7843521000006113	Rapidly progressive dementia	^ESCTRA784352
7973121000006114	Hallucinations co-occurrent and due to late onset dementia	^ESCTHA797312
12106371000006111	BPSD - behavioral and psychological symptoms of dementia	^ESCT1210637
12106381000006114	BPSD - behavioural and psychological symptoms of dementia	^ESCT1210638
12106351000006118	Behavioural and psychological symptoms of dementia	^ESCT1210635
12106391000006112	Behavioral and psychological symptoms of dementia	^ESCT1210639
2290431000000110	Antipsychotic drug therapy for dementia	8BPa
13909641000006114	Agitation due to dementia	^ESCT1390964
13909621000006119	Aggression due to dementia	^ESCT1390962
408401000000119	Dementia annual review	6AB
915111000006112	Dementia review	EMISNQDE1
915121000006116	Dementia review with third party	EMISNQDE2
8024691000006119	Epileptic dementia with behavioural disturbance	^ESCTEP802469
7863681000006115	Epilepsy co-occurrent and due to dementia	^ESCTEP786368
2284371000000110	Assessment of psychotic and behavioural symptoms of dementia	38C13
2345881000000115	Dementia advance care plan agreed	8CSA
2743011000000119	Dementia advance care plan agreed	^ESCT1172377
2345801000000113	Dementia advance care plan declined	8IAe0
2445691000000113	Dementia advance care plan review declined	8IAe2
12116721000006112	Dementia advance care planning declined	^ESCT1211672
2742971000000119	Offer of dementia advance care planning review declined	^ESCT1172375
2742991000000115	Review of dementia advance care plan	^ESCT1172376
1834091000006117	Dementia care plan	EMISNQDE37
2248021000000110	Dementia care plan	8CMZ
11930781000006119	Dementia care plan	^ESCT1193078
2439591000000113	Dementia care plan agreed	8CMZ0
2439671000000110	Dementia care plan declined	8CMZ2
2439711000000111	Dementia care plan review declined	8CMZ3
2439631000000113	Dementia care plan reviewed	8CMZ1
14479161000006110	Review of dementia care plan	^ESCT1447916
407461000000119	Dementia monitoring	66h
406841000000115	Dementia monitoring administration	9Ou
408361000000111	Dementia monitoring first letter	9Ou1
1856601000006111	Dementia monitoring in primary care	EMISNQDE41
1856611000006114	Dementia monitoring in secondary care	EMISNQDE42

8214731000006110	Dementia monitoring invitation	^ESCTDE821473
408421000000111	Dementia monitoring second letter	9Ou2
408501000000115	Dementia monitoring telephone invitation	9Ou5
408461000000115	Dementia monitoring third letter	9Ou3
408481000000112	Dementia monitoring verbal invitation	9Ou4
1856631000006115	Did not attend dementia monitoring	EMISNQDI117
1916981000006111	Dementia medication review	EMISNQDE54
2403101000000116	Dementia medication review	8BM02
2620431000000115	Shared care prescribing of drug for dementia	8BM50
2620471000000118	Shared care prescribing of drugs for dementia declined	8BM60
7794741000006116	Shared care prescribing protocol of drugs for dementia	^ESCTSH779474
2345931000000111	Review of dementia advance care plan	8CMG2
2445471000000112	Dementia advance care plan	8CMe0
2345931000000111	Review of dementia advance care plan	8CMG2
2445471000000112	Dementia advance care plan	8CMe0
2345931000000111	Review of dementia advance care plan	8CMG2
2445471000000112	Dementia advance care plan	8CMe0
**Only Used to Exclude Prevalent Dementia**		
2127581000000115	Suspected dementia	1JA2
251625013	H/O: dementia	1461
4539871000006116	History of dementia	^ESCTHI453987
1972431000006113	Mental & behav dis due to cannabinoids: resid & late-onset psychot dis, dementia	EMISICD10|F1273
1971661000006114	Mental & behav dis due to seds/hypntcs: resid & late-onset psychot dis, dementia	EMISICD10|F1373
1974931000006114	Mental & behav dis due to tobacco: resid & late-onset psychot dis, dementia	EMISICD10|F1773
1975591000006119	Mental & behav dis due to use alcohol: resid & late-onset psychot dis, dementia	EMISICD10|F1073
1973941000006119	Mental & behav dis due to use cocaine: resid & late-onset psychot dis, dementia	EMISICD10|F1473
1971701000006118	Mental & behav dis due to use opioids: resid & late-onset psychot dis, dementia	EMISICD10|F1173
1973711000006116	Mental and behav dis due to hallucinogens: resid & late-onset psychot dis, dementia	EMISICD10|F1673
1976091000006118	Mental and behav dis due to other stimulants inc caffeine: resid/late-onset psycht dis, dementia	EMISICD10|F1573
1973171000006112	Mental and behav dis due to vol solvents: resid & late-onset psychotic dis, dementia	EMISICD10|F1873
1974271000006119	Mental and behav dis mlti drg use/oth psych subs: resid/late psychot dis, dementia	EMISICD10|F1973

Supplementary Table 5. READ Codes for Dementia in CPRD GOLD.

MedCode	Readcode	Readterm
**Alzheimer's Disease**		
44674	E002.00	Senile dementia with depressive or paranoid features
60059	Eu00012	[X]Primary degen dementia, Alzheimer's type, presenile onset
61528	Eu00013	[X]Alzheimer's disease type 2
25704	Eu00011	[X]Presenile dementia, Alzheimer's type
1917	F110.00	Alzheimer's disease
49263	Eu00000	[X]Dementia in Alzheimer's disease with early onset
37015	E003.00	Senile dementia with delirium
11379	Eu00112	[X]Senile dementia, Alzheimer's type
38678	Eu00100	[X]Dementia in Alzheimer's disease with late onset
7664	Eu00.00	[X]Dementia in Alzheimer's disease
41089	E002z00	Senile dementia with depressive or paranoid features NOS
43346	Eu00113	[X]Primary degen dementia of Alzheimer's type, senile onset
46762	Eu00111	[X]Alzheimer's disease type 1
1916	E00..11	Senile dementia
27759	Eu02z16	[X] Senile dementia, depressed or paranoid type
4357	Eu02z14	[X] Senile dementia NOS
21887	E002100	Senile dementia with depression
32057	F110100	Alzheimer's disease with late onset
18386	E002000	Senile dementia with paranoia
8195	Eu00z11	[X]Alzheimer's dementia unspec
16797	F110000	Alzheimer's disease with early onset
1350	E00..12	Senile/presenile dementia
30706	Eu00200	[X]Dementia in Alzheimer's dis, atypical or mixed type
7323	E000.00	Uncomplicated senile dementia
59122	Fyu3000	[X]Other Alzheimer's disease
29386	Eu00z00	[X]Dementia in Alzheimer's disease, unspecified
27935	Eu02z15	[X] Senile psychosis NOS
**Vascular Dementia**		
43089	E004000	Uncomplicated arteriosclerotic dementia
6578	Eu01.00	[X]Vascular dementia
19477	E004.00	Arteriosclerotic dementia
55467	E004200	Arteriosclerotic dementia with paranoia
43292	E004300	Arteriosclerotic dementia with depression
42279	E004z00	Arteriosclerotic dementia NOS
9565	Eu01.11	[X]Arteriosclerotic dementia
46488	Eu01000	[X]Vascular dementia of acute onset
8634	E004.11	Multi infarct dementia
31016	Eu01300	[X]Mixed cortical and subcortical vascular dementia
55313	Eu01y00	[X]Other vascular dementia
11175	Eu01100	[X]Multi-infarct dementia
55838	Eu01111	[X]Predominantly cortical dementia
8934	Eu01200	[X]Subcortical vascular dementia
56912	E004100	Arteriosclerotic dementia with delirium
19393	Eu01z00	[X]Vascular dementia, unspecified
68194	F21y211	Binswanger's encephalopathy
5095	F21y200	Binswanger's disease
**Others**		
41185	Eu02400	[X]Dementia in human immunodef virus [HIV] disease
26323	Eu10711	[X]Alcoholic dementia NOS
62132	E02y100	Drug-induced dementia
26270	Eu02500	[X]Lewy body dementia
28402	Eu02000	[X]Dementia in Pick's disease
114713	F118100	Frontotemporal dementia

54106	Eu02100	[X]Dementia in Creutzfeldt-Jakob disease
27342	E012.11	Alcoholic dementia NOS
37014	Eu02200	[X]Dementia in Huntington's disease
9509	Eu02300	[X]Dementia in Parkinson's disease
54505	E012.00	Other alcoholic dementia
11136	F111.00	Pick's disease
**Unspecified Dementia**		
42602	E001000	Uncomplicated presenile dementia
38438	E001z00	Presenile dementia NOS
15165	E001.00	Presenile dementia
49513	E001100	Presenile dementia with delirium
60726	3AE3.00	GDS level 4 - moderate cognitive decline
70057	3AE4.00	GDS level 5 - moderately severe cognitive decline
72520	3AE6.00	GDS level 7 - very severe cognitive decline
64267	Eu02y00	[X]Dementia in other specified diseases classif elsewhere
25386	E041.00	Dementia in conditions EC
94717	3AE5.00	GDS level 6 - severe cognitive decline
53446	Eu04100	[X]Delirium superimposed on dementia
48501	Eu02z11	[X] Presenile dementia NOS
30032	E001200	Presenile dementia with paranoia
34944	Eu02z13	[X] Primary degenerative dementia NOS
12621	Eu02.00	[X]Dementia in other diseases classified elsewhere
4693	Eu02z00	[X] Unspecified dementia
27677	E001300	Presenile dementia with depression
47619	Eu02z12	[X] Presenile psychosis NOS
51494	E00y.11	Presbyophrenic psychosis
109047	8BPa.00	Antipsychotic drug therapy for dementia
109737	8BM0200	Dementia medication review
55023	66h..00	Dementia monitoring
85853	9Ou..00	Dementia monitoring administration
49674	9Ou1.00	Dementia monitoring first letter
83576	9Ou2.00	Dementia monitoring second letter
65235	9Ou5.00	Dementia monitoring telephone invite
89036	9Ou3.00	Dementia monitoring third letter
89037	9Ou4.00	Dementia monitoring verbal invite
12710	6AB..00	Dementia annual review
106311	8CMZ.00	Dementia care plan
109708	8CMZ100	Dementia care plan reviewed
108268	8CMG200	Review of dementia advance care plan
109731	8CMZ000	Dementia care plan agreed
108391	8IAe000	Dementia advance care plan declined
110123	8CMZ300	Dementia care plan review declined
110075	8IAe200	Dementia advance care plan review declined
**Only Used to Exclude Prevalent Dementia**		
5931	1461	H/O: dementia
104155	1JA2.00	Suspected dementia

Supplementary Table 6. ICD Codes Used to Exclude Dementia and Cognitive Deficits.

ICD	Description
F00	Dementia in Alzheimer disease
F01	Vascular dementia
F02	Dementia in other diseases classified elsewhere
F03	Unspecified dementia
F05.1	Delirium superimposed on dementia
F10.7	Mental and behavioural disorders due to use of alcohol (Residual and late-onset psychotic disorder)
F11.7	Mental and behavioural disorders due to use of opioids (Residual and late-onset psychotic disorder)
F12.7	Mental and behavioural disorders due to use of cannabinoids (Residual and late-onset psychotic disorder)
F13.7	Mental and behavioural disorders due to use of sedatives or hypnotics (Residual and late-onset psychotic disorder)
F14.7	Mental and behavioural disorders due to use of cocaine (Residual and late-onset psychotic disorder)
F15.7	Mental and behavioural disorders due to use of other stimulants, including caffeine (Residual and late-onset psychotic disorder)
F16.7	Mental and behavioural disorders due to use of hallucinogens (Residual and late-onset psychotic disorder)
F17.7	Mental and behavioural disorders due to use of tobacco (Residual and late-onset psychotic disorder)
F18.7	Mental and behavioural disorders due to use of volatile solvents (Residual and late-onset psychotic disorder)
F19.7	Mental and behavioural disorders due to multiple drug use and use of other psychoactive substances (Residual and late-onset psychotic disorder)
G30	Alzheimer disease
G31.0	Circumscribed brain atrophy
F06.7	Mild cognitive disorder
R47.0	Dysphasia and aphasia
R48.1	Agnosia
R48.2	Apraxia

Supplementary Table 7. Covariate Ascertainment.

Covariates	Data Type	Data Source	Lookback, Days	Notes
Index Year	Groups by years	Primary Care	[0,0]	Calendar years.
Age	Integers in Years	Primary Care	[0,0]	Modeled as a restricted cubic spline function with 5 percentile knots.
Gender	Males, Females	Primary Care	--	Individuals with indeterminate gender prescribed the study drugs were not included.
Country	England, Wales, Northern Ireland, Scottland	Primary Care	[0,0]	Practice-level.
Multiple Deprivation Index	"1", "2", "3", "4", "5", "Outside of England"	Primary Care	[0,0]	Practice-level.<br>1 = least deprived; 5 = most deprived
Smoking	Ever, Never, Missing	Primary Care	[-Inf, 0]	--
Diabetes Duration, years	Continuous Years	Primary care, HES APC	[-Inf, 0]	First diagnosis of type 2 diabetes, first HbA1c ≥6.5% (or 48 mmol/mol), or first prescription of a glucose-lowering medication.<br><br>Modeled as a restricted cubic spline function with 5 percentile knots.
Body Mass Index (BMI), kg/m²	"≤24.9", "25.0-29.9", "≥30.0", Missing	Primary care	[-365, 0]	Latest BMI record or latest weight measure during the lookback window. Height measures were based on the latest measure in adulthood.
HbA1c, %	"≤7.0", "7.1-8.0", ">8.0", Missing	Primary care	[-365, 0]	Latest measure during the lookback window. Measures in mmol/mol were converted to %.
eGFR, mL/min/1.73m²	"<30", "30-45", "45-60", "60-90", "≥90", Missing	Primary care	[-365, 0]	Latest measure during the lookback window. When creatinine levels were available, eGFR measures were derived using the CKD-EPI equation [1].
Influenza Vaccination	Yes/No	Primary care	[-2*365, 0]	--
Number of primary care visits in the past year	Counts	Primary Care	[-365, -1]	Modeled as a restricted cubic spline function with 5 percentile knots.
Number of hospitalizations in the past year	0, 1, 2, 3+, Missing	HES APC	[-365, 0]	Hospitalizations were selected based on the discharge dates.
Comorbidities	Yes/No for each condition	Primary Care, HES APC	[-3*365, 0]	1. Acute Kidney Injury<br>2. Alcoholic Liver Disease<br>3. Angina<br>4. Atrial Fibrillation/Flutter<br>5. Bipolar Disorder<br>6. Cancer<br>7. Cataract<br>8. Cerebrovascular disease<br>9. Chronic kidney disease

				10. Chronic Obstructive Pulmonary Disease<br>11. Cirrhosis<br>12. Coronary Artery Disease<br>13. Deep Vein Thrombosis<br>14. Delirium<br>15. Depression<br>16. Diabetic Neuropathy<br>17. Diabetic Retinopathy<br>18. Epilepsy<br>19. Fractures<br>20. Genital Tract Infection<br>21. Glaucoma<br>22. Gout<br>23. Hearing Impairment<br>24. Heart Failure<br>25. Hypertension<br>26. Hypoglycemia<br>27. Inflammatory Bowel Disease<br>28. Ketoacidosis<br>29. Kidney Stones<br>30. Myocardial Infarction<br>31. Non-alcoholic Fatty Liver Disease<br>32. Osteoporosis<br>33. Pancreatitis<br>34. Parkinsonism<br>35. Peripheral artery disease or aortic atherosclerotic disease<br>36. Pneumonia<br>37. Psychotic Disorders<br>38. Pulmonary Embolism<br>39. Sleep Apnea<br>40. Traumatic Brain Injury<br>41. Urinary Tract Infection
Procedures	Yes/No for each procedure	Primary Care, HES APC	[-Inf, 0]	1. Coronary Revascularization<br>2. Lower Extremity Amputation
Glucose-Lowering Medications	Yes/No for each drug category	Primary Care	[-365, 0]	1. Metformin<br>2. Thiazolidinediones<br>3. Sulphonylureas<br>4. Acarbose<br>5. Meglitinides<br>6. Insulin<br>7. DPP4 inhibitors (when GLP1 vs SGLT2)<br>8. SGLT2 Inhibitors (when GLP1 vs DPP4)

				9. GLP1 Receptor Agonists (when SGLT2 vs DPP4)
Other Medications	Yes/No for each drug category	Primary Care	[-365, 0]	1. Allopurinol<br>2. Angiotensin Converting Enzyme Inhibitors<br>3. Angiotensin Receptor Blockers<br>4. Antibiotics<br>5. Antiepileptic Drugs<br>6. Antihistamines<br>7. Antimuscarinic Bronchodilators<br>8. Antiparkinson Drugs (antimuscarinic)<br>9. Antiparkinson Drugs (dopaminergic)<br>10. Antiplatelets<br>11. Antipsychotics<br>12. Antivirals<br>13. Aromatase inhibitors<br>14. Aspirin<br>15. Benzodiazepines<br>16. Beta blockers<br>17. Beta-2 Adrenergic Receptor Agonists<br>18. Bladder antimuscarinics<br>19. Calcium channel blockers<br>20. Chemotherapy<br>21. Colchicine<br>22. Digoxin<br>23. Fibrates<br>24. Glucocorticoids<br>25. H2 receptor antagonists<br>26. Heparin<br>27. Hydroxychloroquine<br>28. Loop Diuretics<br>29. Methotrexate<br>30. Mirabegron<br>31. Nonsteroidal anti-inflammatory drugs<br>32. Oral Anticoagulants<br>33. Other Antidepressants<br>34. Potassium sparing diuretics<br>35. Proton Pump Inhibitors<br>36. Selective Serotonin Reuptake Inhibitors<br>37. Statins<br>38. Tamoxifen<br>39. Thiazide diuretics

				40. Thyroid Replacement Therapy<br>41. Tricyclic antidepressants<br>42. Z-Drugs

The diagnostic codes used to define comorbidities, medications, and procedures are available in https://gitea.com/joeywu/CPRD_2545

Supplementary Table 8. Breakdown of Molecules.

	Before Weighting		After Weighting	
Molecules	Count	Within-Class Proportion	Count	Within-Class Proportion
**GLP1 Receptor Agonists vs DPP4 Inhibitors**				
Albiglutide or Lixisenatide	768	3.5%	569	4.2%
Dulaglutide	6098	27.7%	3486	25.5%
Exenatide	5495	24.9%	3655	26.7%
Liraglutide	5739	26.0%	3943	28.8%
Semaglutide (subcutaneous)	3942	17.9%	2040	14.9%
Alogliptin	25799	15.1%	2272	16.6%
Linagliptin	42866	25.1%	2899	21.2%
Saxagliptin	9623	5.6%	672	4.9%
Sitagliptin	87652	51.4%	7440	54.3%
Vildagliptin	4577	2.7%	412	3.0%
**SGLT2 Inhibitors vs DPP4 Inhibitors**				
Canagliflozin	6220	11.9%	3676	14.4%
Dapagliflozin	26492	50.7%	12449	48.8%
Empagliflozin	19496	37.3%	9377	36.7%
Ertugliflozin	66	0.1%	31	0.1%
Alogliptin	24785	19.1%	6848	26.8%
Linagliptin	42259	32.6%	7645	29.9%
Saxagliptin	6227	4.8%	797	3.1%
Sitagliptin	55162	42.6%	10112	39.6%
Vildagliptin	1038	0.8%	131	0.5%
**GLP1 Receptor Agonists vs SGLT2 Inhibitors**				
Albiglutide or Lixisenatide	1692	7.2%	904	6.4%
Dulaglutide	7787	33.3%	5392	37.9%
Exenatide	2387	10.2%	1075	7.6%
Liraglutide	7232	30.9%	3756	26.4%
Semaglutide (subcutaneous)	4302	18.4%	3086	21.7%
Canagliflozin	12317	13.3%	2165	15.2%
Dapagliflozin	45128	48.9%	7055	49.6%
Empagliflozin	34652	37.6%	4970	35.0%
Ertugliflozin	183	0.2%	24	0.2%

Abbreviations: GLP1 = glucagon-like peptide-1; SGLT2 = sodium-glucose cotransporter 2; DPP4 = dipeptidyl peptidase-4

Supplementary Table 9. Full Baseline Characteristics of Initiators of GLP1 receptor agonists versus DPP4 Inhibitors.

	Before weighting			After weighting		
**Characteristics**	**GLP1 (N=22042)**	**DPP4 (N=170517)**	**SMD**	**GLP1 (N=13695)**	**DPP4 (N=13695)**	**aSMD**
Age, years						
Mean (SD)	66.1 (5.3)	71.2 (7.8)	-0.767	66.9 (5.6)	66.9 (5.6)	<0.001
Median (IQR)	65 (62-69)	70 (65-77)		66 (62-70)	66 (62-70)	
Male	12328 (55.9%)	96480 (56.6%)	-0.013	7615 (55.6%)	7615 (55.6%)	<0.001
Country						
England	20935 (95.0%)	166059 (97.4%)	-0.126	13055 (95.3%)	13055 (95.3%)	<0.001
Northen Ireland	214 (1.0%)	1019 (0.6%)	0.042	121 (0.9%)	121 (0.9%)	<0.001
Scotland	408 (1.9%)	1495 (0.9%)	0.084	229 (1.7%)	229 (1.7%)	<0.001
Wales	485 (2.2%)	1944 (1.1%)	0.083	288 (2.1%)	288 (2.1%)	<0.001
Multiple Deprivation Index						
1	3388 (15.4%)	24657 (14.5%)	0.026	2028 (14.8%)	2028 (14.8%)	<0.001
2	3309 (15.0%)	25395 (14.9%)	0.003	2036 (14.9%)	2036 (14.9%)	<0.001
3	4209 (19.1%)	31992 (18.8%)	0.009	2619 (19.1%)	2619 (19.1%)	<0.001
4	4952 (22.5%)	40410 (23.7%)	-0.029	3098 (22.6%)	3098 (22.6%)	<0.001
5	5077 (23.0%)	43605 (25.6%)	-0.059	3274 (23.9%)	3274 (23.9%)	<0.001
Outside of England	1107 (5.0%)	4458 (2.6%)	0.126	639 (4.7%)	639 (4.7%)	<0.001
Smoking						
Ever	18022 (81.8%)	135439 (79.4%)	0.059	11130 (81.3%)	11130 (81.3%)	<0.001
Never	3970 (18.0%)	34938 (20.5%)	-0.063	2537 (18.5%)	2537 (18.5%)	<0.001
Missing	50 (0.2%)	140 (0.1%)	0.037	27 (0.2%)	27 (0.2%)	<0.001
Diabetes duration, years						
Mean (SD)	11.2 (6.4)	9.7 (6.2)	0.224	10.3 (6.2)	10.3 (6.2)	<0.001
Median (IQR)	10.4 (6.4-15.1)	8.9 (5.2-13.2)		9.5 (5.8-13.9)	9.5 (5.8-13.9)	
Body Mass Index, kg/m²						
≤24.9	206 (0.9%)	19953 (11.7%)	-0.454	185 (1.4%)	185 (1.4%)	<0.001
25.0-29.9	2058 (9.3%)	51516 (30.2%)	-0.543	1618 (11.8%)	1618 (11.8%)	<0.001
≥30.0	18345 (83.2%)	83743 (49.1%)	0.773	10919 (79.7%)	10919 (79.7%)	<0.001
Missing	1433 (6.5%)	15305 (9.0%)	-0.093	971 (7.1%)	971 (7.1%)	<0.001
HbA1c, %						
≤7.0	1876 (8.5%)	16419 (9.6%)	-0.039	1230 (9.0%)	1230 (9.0%)	<0.001
7.1-8.0	4192 (19.0%)	54254 (31.8%)	-0.297	2932 (21.4%)	2932 (21.4%)	<0.001
>8.0	15459 (70.1%)	95817 (56.2%)	0.292	9196 (67.1%)	9196 (67.1%)	<0.001
Missing	515 (2.3%)	4027 (2.4%)	-0.002	337 (2.5%)	337 (2.5%)	<0.001
eGFR, mL/min/1.73m²						
≥90	8733 (39.6%)	50915 (29.9%)	0.206	5200 (38.0%)	5200 (38.0%)	<0.001
60-90	9152 (41.5%)	73718 (43.2%)	-0.035	5768 (42.1%)	5768 (42.1%)	<0.001
45-60	2190 (9.9%)	21911 (12.8%)	-0.092	1429 (10.4%)	1429 (10.4%)	<0.001
30-45	824 (3.7%)	15016 (8.8%)	-0.210	586 (4.3%)	586 (4.3%)	<0.001
<30	120 (0.5%)	3748 (2.2%)	-0.143	91 (0.7%)	91 (0.7%)	<0.001
Missing	1023 (4.6%)	5209 (3.1%)	0.083	621 (4.5%)	621 (4.5%)	<0.001
Flu vaccination	19545 (88.7%)	147628 (86.6%)	0.064	12052 (88.0%)	12052 (88.0%)	<0.001
Number of primary care visits						
Mean (SD)	35.0 (19.1)	30.2 (17.5)	0.258	33.5 (18.6)	33.5 (18.7)	<0.001
Median (IQR)	31 (21-44)	26 (18-38)		29 (20-42)	29 (20-42)	
Number of Hospitalizations						
0	13035 (59.1%)	96784 (56.8%)	0.048	8118 (59.3%)	8118 (59.3%)	<0.001

1	2749 (12.5%)	22453 (13.2%)	-0.021	1725 (12.6%)	1725 (12.6%)	<0.001
2	1038 (4.7%)	8891 (5.2%)	-0.023	649 (4.7%)	649 (4.7%)	<0.001
≥3	732 (3.3%)	7475 (4.4%)	-0.055	469 (3.4%)	469 (3.4%)	<0.001
Missing	4488 (20.4%)	34914 (20.5%)	-0.003	2734 (20.0%)	2734 (20.0%)	<0.001
Year of Entry						
2007	102 (0.5%)	438 (0.3%)	0.034	63 (0.5%)	63 (0.5%)	<0.001
2008	742 (3.4%)	2343 (1.4%)	0.131	416 (3.0%)	416 (3.0%)	<0.001
2009	1353 (6.1%)	6285 (3.7%)	0.114	823 (6.0%)	823 (6.0%)	<0.001
2010	1765 (8.0%)	11701 (6.9%)	0.044	1199 (8.8%)	1199 (8.8%)	<0.001
2011	1221 (5.5%)	10941 (6.4%)	-0.037	894 (6.5%)	894 (6.5%)	<0.001
2012	1494 (6.8%)	12258 (7.2%)	-0.016	1050 (7.7%)	1050 (7.7%)	<0.001
2013	951 (4.3%)	11618 (6.8%)	-0.109	718 (5.2%)	718 (5.2%)	<0.001
2014	763 (3.5%)	11625 (6.8%)	-0.152	594 (4.3%)	594 (4.3%)	<0.001
2015	847 (3.8%)	13486 (7.9%)	-0.174	644 (4.7%)	644 (4.7%)	<0.001
2016	810 (3.7%)	14804 (8.7%)	-0.209	606 (4.4%)	606 (4.4%)	<0.001
2017	1015 (4.6%)	15456 (9.1%)	-0.177	713 (5.2%)	713 (5.2%)	<0.001
2018	1180 (5.4%)	15411 (9.0%)	-0.143	802 (5.9%)	802 (5.9%)	<0.001
2019	1852 (8.4%)	13817 (8.1%)	0.011	1109 (8.1%)	1109 (8.1%)	<0.001
2020	1716 (7.8%)	9910 (5.8%)	0.078	969 (7.1%)	969 (7.1%)	<0.001
2021	2982 (13.5%)	11021 (6.5%)	0.237	1536 (11.2%)	1536 (11.2%)	<0.001
2022	3249 (14.7%)	9403 (5.5%)	0.309	1559 (11.4%)	1559 (11.4%)	<0.001
Cerebrovascular disease	605 (2.7%)	6547 (3.8%)	-0.061	389 (2.8%)	389 (2.8%)	<0.001
Peripheral artery disease or aortic atherosclerotic disease	855 (3.9%)	6287 (3.7%)	0.010	492 (3.6%)	492 (3.6%)	<0.001
Atrial fibrillation	1592 (7.2%)	15212 (8.9%)	-0.062	1014 (7.4%)	1014 (7.4%)	<0.001
Myocardial infarction	545 (2.5%)	4661 (2.7%)	-0.016	329 (2.4%)	329 (2.4%)	<0.001
Angina	1612 (7.3%)	12904 (7.6%)	-0.010	982 (7.2%)	982 (7.2%)	<0.001
Heart failure	1191 (5.4%)	10295 (6.0%)	-0.027	720 (5.3%)	720 (5.3%)	<0.001
Coronary artery disease	3238 (14.7%)	26822 (15.7%)	-0.029	1999 (14.6%)	1999 (14.6%)	<0.001
Hypertension	12772 (57.9%)	99660 (58.4%)	-0.01	7953 (58.1%)	7953 (58.1%)	<0.001
Deep vein thrombosis	222 (1.0%)	1557 (0.9%)	0.010	134 (1.0%)	134 (1.0%)	<0.001
Pulmonary embolism	188 (0.9%)	1228 (0.7%)	0.015	106 (0.8%)	106 (0.8%)	<0.001
Hearing loss	854 (3.9%)	8853 (5.2%)	-0.063	544 (4.0%)	544 (4.0%)	<0.001
Glaucoma	407 (1.8%)	5350 (3.1%)	-0.083	270 (2.0%)	270 (2.0%)	<0.001
Cataract	1588 (7.2%)	17395 (10.2%)	-0.106	1002 (7.3%)	1002 (7.3%)	<0.001
Diabetic retinopathy	4606 (20.9%)	29613 (17.4%)	0.090	2619 (19.1%)	2619 (19.1%)	<0.001
Psychotic disorders	199 (0.9%)	1844 (1.1%)	-0.018	129 (0.9%)	129 (0.9%)	<0.001
Bipolar disorder	87 (0.4%)	551 (0.3%)	0.012	51 (0.4%)	51 (0.4%)	<0.001
Depression	2845 (12.9%)	13465 (7.9%)	0.165	1604 (11.7%)	1604 (11.7%)	<0.001
Delirium	54 (0.2%)	889 (0.5%)	-0.045	37 (0.3%)	37 (0.3%)	<0.001
Traumatic brain injury	34 (0.2%)	336 (0.2%)	-0.010	21 (0.2%)	21 (0.2%)	<0.001
Parkinsonism	42 (0.2%)	667 (0.4%)	-0.037	30 (0.2%)	30 (0.2%)	<0.001
Sleep apnea	1374 (6.2%)	3247 (1.9%)	0.220	662 (4.8%)	662 (4.8%)	<0.001
Diabetic neuropathy	762 (3.5%)	3647 (2.1%)	0.080	395 (2.9%)	395 (2.9%)	<0.001
Epilepsy	146 (0.7%)	1339 (0.8%)	-0.014	99 (0.7%)	99 (0.7%)	<0.001
Hypoglycemia	463 (2.1%)	3345 (2.0%)	0.010	257 (1.9%)	257 (1.9%)	<0.001
Ketoacidosis	106 (0.5%)	298 (0.2%)	0.054	45 (0.3%)	45 (0.3%)	<0.001
Genital tract infection	1179 (5.3%)	5445 (3.2%)	0.107	659 (4.8%)	659 (4.8%)	<0.001
Urinary tract infection	1860 (8.4%)	16028 (9.4%)	-0.034	1176 (8.6%)	1176 (8.6%)	<0.001
Acute kidney injury	661 (3.0%)	7302 (4.3%)	-0.069	418 (3.0%)	418 (3.0%)	<0.001
Chronic kidney disease	3330 (15.1%)	33725 (19.8%)	-0.123	2133 (15.6%)	2133 (15.6%)	<0.001
Kidney stones	398 (1.8%)	2874 (1.7%)	0.009	251 (1.8%)	251 (1.8%)	<0.001

Pneumonia	669 (3.0%)	5639 (3.3%)	-0.016	397 (2.9%)	397 (2.9%)	<0.001
Chronic obstructive pulmonary disease	2114 (9.6%)	15949 (9.4%)	0.008	1317 (9.6%)	1317 (9.6%)	<0.001
Fractures	961 (4.4%)	8131 (4.8%)	-0.02	596 (4.4%)	596 (4.4%)	<0.001
Osteoporosis	286 (1.3%)	4010 (2.4%)	-0.079	198 (1.4%)	198 (1.4%)	<0.001
Pancreatitis	61 (0.3%)	725 (0.4%)	-0.025	39 (0.3%)	39 (0.3%)	<0.001
Non-alcoholic fatty liver disease	821 (3.7%)	3480 (2.0%)	0.101	444 (3.2%)	444 (3.2%)	<0.001
Alcoholic liver disease	85 (0.4%)	538 (0.3%)	0.012	52 (0.4%)	52 (0.4%)	<0.001
Cirrhosis	184 (0.8%)	886 (0.5%)	0.038	96 (0.7%)	96 (0.7%)	<0.001
Inflammatory bowel disease	138 (0.6%)	1494 (0.9%)	-0.029	94 (0.7%)	94 (0.7%)	<0.001
Cancer	1639 (7.4%)	16371 (9.6%)	-0.078	1056 (7.7%)	1056 (7.7%)	<0.001
Gout	935 (4.2%)	8198 (4.8%)	-0.027	600 (4.4%)	600 (4.4%)	<0.001
Revascularization	2191 (9.9%)	17118 (10.0%)	-0.003	1309 (9.6%)	1309 (9.6%)	<0.001
Lower extremity amputation	166 (0.8%)	827 (0.5%)	0.034	87 (0.6%)	87 (0.6%)	<0.001
Metformin	19796 (89.8%)	148898 (87.3%)	0.078	12330 (90.0%)	12330 (90.0%)	<0.001
Thiazolidinediones	3635 (16.5%)	19081 (11.2%)	0.154	2460 (18.0%)	2460 (18.0%)	<0.001
Sulfonylureas	10489 (47.6%)	79285 (46.5%)	0.022	6865 (50.1%)	6865 (50.1%)	<0.001
Acarbose	112 (0.5%)	656 (0.4%)	0.019	69 (0.5%)	69 (0.5%)	<0.001
Meglitinides	193 (0.9%)	1201 (0.7%)	0.019	131 (1.0%)	131 (1.0%)	<0.001
Insulin	6600 (29.9%)	8032 (4.7%)	0.707	2388 (17.4%)	2388 (17.4%)	<0.001
SGLT2 inhibitors	5316 (24.1%)	7971 (4.7%)	0.576	2306 (16.8%)	2306 (16.8%)	<0.001
Statins	18880 (85.7%)	140742 (82.5%)	0.085	11612 (84.8%)	11612 (84.8%)	<0.001
Fibrates	676 (3.1%)	3574 (2.1%)	0.061	384 (2.8%)	384 (2.8%)	<0.001
Steroids	10081 (45.7%)	73381 (43.0%)	0.054	6178 (45.1%)	6178 (45.1%)	<0.001
Nonsteroidal anti-inflammatory drugs	5542 (25.1%)	41351 (24.3%)	0.021	3484 (25.4%)	3484 (25.4%)	<0.001
Aspirin	8177 (37.1%)	60688 (35.6%)	0.031	5073 (37.0%)	5073 (37.0%)	<0.001
Anticoagulants	2037 (9.2%)	17092 (10.0%)	-0.027	1252 (9.1%)	1252 (9.1%)	<0.001
Heparin	120 (0.5%)	1072 (0.6%)	-0.011	80 (0.6%)	80 (0.6%)	<0.001
Antiplatelets	1742 (7.9%)	14107 (8.3%)	-0.014	1038 (7.6%)	1038 (7.6%)	<0.001
Angiotensin converting enzyme inhibitors	11973 (54.3%)	83937 (49.2%)	0.102	7282 (53.2%)	7282 (53.2%)	<0.001
Angiotensin receptor blockers	5444 (24.7%)	38652 (22.7%)	0.048	3335 (24.3%)	3335 (24.3%)	<0.001
Beta-blockers	6781 (30.8%)	53082 (31.1%)	-0.008	4164 (30.4%)	4164 (30.4%)	<0.001
Calcium channel blockers	8631 (39.2%)	65775 (38.6%)	0.012	5330 (38.9%)	5330 (38.9%)	<0.001
Loop diuretics	4099 (18.6%)	27559 (16.2%)	0.064	2459 (18.0%)	2459 (18.0%)	<0.001
Thiazide diuretics	4882 (22.1%)	35863 (21.0%)	0.027	3058 (22.3%)	3058 (22.3%)	<0.001
Potassium sparing diuretics	1267 (5.7%)	7939 (4.7%)	0.049	730 (5.3%)	730 (5.3%)	<0.001
Digoxin	557 (2.5%)	6279 (3.7%)	-0.067	366 (2.7%)	366 (2.7%)	<0.001
Methotrexate	222 (1.0%)	1559 (0.9%)	0.010	140 (1.0%)	140 (1.0%)	<0.001
Hydroxychloroquine	87 (0.4%)	539 (0.3%)	0.013	51 (0.4%)	51 (0.4%)	<0.001
Allopurinol	1226 (5.6%)	9195 (5.4%)	0.007	769 (5.6%)	769 (5.6%)	<0.001
Colchicine	289 (1.3%)	2520 (1.5%)	-0.014	185 (1.3%)	185 (1.3%)	<0.001
Antibiotics	10926 (49.6%)	80423 (47.2%)	0.048	6761 (49.4%)	6761 (49.4%)	<0.001
Antivirals	314 (1.4%)	2285 (1.3%)	0.007	193 (1.4%)	193 (1.4%)	<0.001
Proton pump inhibitors	9889 (44.9%)	71047 (41.7%)	0.065	5977 (43.6%)	5977 (43.6%)	<0.001
H2 receptor antagonists	692 (3.1%)	6848 (4.0%)	-0.047	448 (3.3%)	448 (3.3%)	<0.001
Tamoxifen	48 (0.2%)	471 (0.3%)	-0.012	34 (0.2%)	34 (0.2%)	<0.001
Aromatase inhibitors	159 (0.7%)	1426 (0.8%)	-0.013	102 (0.7%)	102 (0.7%)	<0.001

Chemotherapy	307 (1.4%)	2495 (1.5%)	-0.006	193 (1.4%)	193 (1.4%)	<0.001
Thyroid replacement therapy	2612 (11.9%)	17607 (10.3%)	0.049	1587 (11.6%)	1587 (11.6%)	<0.001
Beta-3 agonist	173 (0.8%)	911 (0.5%)	0.031	99 (0.7%)	99 (0.7%)	<0.001
Bladder antimuscarinics	1137 (5.2%)	7632 (4.5%)	0.032	692 (5.1%)	692 (5.1%)	<0.001
Antihistamines	2492 (11.3%)	18036 (10.6%)	0.023	1531 (11.2%)	1531 (11.2%)	<0.001
Beta-2 agonists	5061 (23.0%)	32191 (18.9%)	0.100	3047 (22.2%)	3047 (22.2%)	<0.001
Antimuscarinic bronchodilators	1635 (7.4%)	10548 (6.2%)	0.049	975 (7.1%)	975 (7.1%)	<0.001
Antiparkinsonian drugs (dopaminergic)	204 (0.9%)	1429 (0.8%)	0.009	119 (0.9%)	119 (0.9%)	<0.001
Antiparkinsonian drugs (antimuscarinic)	51 (0.2%)	387 (0.2%)	0.001	33 (0.2%)	33 (0.2%)	<0.001
Antipsychotics	1086 (4.9%)	8292 (4.9%)	0.003	660 (4.8%)	660 (4.8%)	<0.001
Benzodiazepines	918 (4.2%)	6863 (4.0%)	0.007	572 (4.2%)	572 (4.2%)	<0.001
Nonbenzodiazepine hypnotics (Z-drugs)	746 (3.4%)	5261 (3.1%)	0.017	447 (3.3%)	447 (3.3%)	<0.001
Antiepileptic drugs	1756 (8.0%)	9725 (5.7%)	0.090	983 (7.2%)	983 (7.2%)	<0.001
Selective serotonin reuptake inhibitors	3498 (15.9%)	15898 (9.3%)	0.198	1933 (14.1%)	1933 (14.1%)	<0.001
Tricyclic antidepressants	3230 (14.7%)	18703 (11.0%)	0.110	1890 (13.8%)	1890 (13.8%)	<0.001
Other antidepressants	1763 (8.0%)	7475 (4.4%)	0.150	911 (6.7%)	911 (6.7%)	<0.001

Abbreviations: GLP1 = glucagon-like peptide-1; SGLT2 = sodium-glucose cotransporter 2; DPP4 = dipeptidyl peptidase-4; aSMD = Absolute standardized mean difference; SD = standard deviation; IQR = interquartile range. aSMDs ≥ 0.1 were considered as meaningful negligible between-group differences [2].

Supplementary Table 10. Full Baseline Characteristics of Initiators of SGLT2 Inhibitors versus DPP4 Inhibitors.

	Before weighting			After weighting		
**Characteristics**	**SGLT2 (N=52274)**	**DPP4 (N=129471)**	**SMD**	**SGLT2 (N=25533)**	**DPP4 (N=25533)**	**aSMD**
Age, years						
Mean (SD)	68.5 (6.7)	71.7 (8.0)	-0.436	69.0 (6.9)	69.0 (6.9)	<0.001
Median (IQR)	67 (63-73)	71 (65-77)		68 (63-73)	68 (63-73)	
Male	32838 (62.8%)	73369 (56.7%)	0.126	15385 (60.3%)	15385 (60.3%)	<0.001
Country						
England	49809 (95.3%)	122895 (94.9%)	0.017	23995 (94.0%)	23995 (94.0%)	<0.001
Northen Ireland	523 (1.0%)	1201 (0.9%)	0.007	293 (1.1%)	293 (1.1%)	<0.001
Scotland	995 (1.9%)	2419 (1.9%)	0.003	619 (2.4%)	619 (2.4%)	<0.001
Wales	947 (1.8%)	2956 (2.3%)	-0.033	626 (2.5%)	626 (2.5%)	<0.001
Multiple Deprivation Index						
1	8353 (16.0%)	18362 (14.2%)	0.050	3786 (14.8%)	3786 (14.8%)	<0.001
2	7923 (15.2%)	18923 (14.6%)	0.015	3734 (14.6%)	3734 (14.6%)	<0.001
3	10276 (19.7%)	23513 (18.2%)	0.038	4810 (18.8%)	4810 (18.8%)	<0.001
4	11479 (22.0%)	29886 (23.1%)	-0.027	5682 (22.3%)	5682 (22.3%)	<0.001
5	11778 (22.5%)	32211 (24.9%)	-0.055	5983 (23.4%)	5983 (23.4%)	<0.001
Outside of England	2465 (4.7%)	6576 (5.1%)	-0.017	1538 (6.0%)	1538 (6.0%)	<0.001
Smoking						
Ever	41985 (80.3%)	103087 (79.6%)	0.017	20402 (79.9%)	20402 (79.9%)	<0.001
Never	10221 (19.6%)	26294 (20.3%)	-0.019	5105 (20.0%)	5105 (20.0%)	<0.001
Missing	68 (0.1%)	90 (0.1%)	0.019	25 (0.1%)	25 (0.1%)	<0.001
Diabetes duration, years						
Mean (SD)	9.6 (6.6)	10.1 (6.4)	-0.069	9.6 (6.4)	9.6 (6.4)	<0.001
Median (IQR)	8.6 (4.6-13.6)	9.3 (5.3-13.8)		8.7 (4.8-13.4)	8.7 (4.8-13.4)	
Body Mass Index, kg/m²						
≤24.9	3943 (7.5%)	15453 (11.9%)	-0.149	2156 (8.4%)	2156 (8.4%)	<0.001
25.0-29.9	13189 (25.2%)	39368 (30.4%)	-0.116	6837 (26.8%)	6837 (26.8%)	<0.001
≥30.0	30058 (57.5%)	61111 (47.2%)	0.207	13961 (54.7%)	13961 (54.7%)	<0.001
Missing	5084 (9.7%)	13539 (10.5%)	-0.024	2579 (10.1%)	2579 (10.1%)	<0.001
HbA1c, %						
≤7.0	7388 (14.1%)	12572 (9.7%)	0.137	2749 (10.8%)	2749 (10.8%)	<0.001
7.1-8.0	13404 (25.6%)	40593 (31.4%)	-0.127	7112 (27.9%)	7112 (27.9%)	<0.001
>8.0	30125 (57.6%)	73598 (56.8%)	0.016	15095 (59.1%)	15095 (59.1%)	<0.001
Missing	1357 (2.6%)	2708 (2.1%)	0.033	578 (2.3%)	578 (2.3%)	<0.001
eGFR, mL/min/1.73m²						
≥90	22275 (42.6%)	38982 (30.1%)	0.262	10384 (40.7%)	10384 (40.7%)	<0.001
60-90	23273 (44.5%)	54214 (41.9%)	0.053	11463 (44.9%)	11463 (44.9%)	<0.001
45-60	3632 (6.9%)	17044 (13.2%)	-0.208	1929 (7.6%)	1929 (7.6%)	<0.001
30-45	1663 (3.2%)	13000 (10.0%)	-0.279	990 (3.9%)	990 (3.9%)	<0.001
<30	397 (0.8%)	3420 (2.6%)	-0.146	241 (0.9%)	241 (0.9%)	<0.001
Missing	1034 (2.0%)	2811 (2.2%)	-0.014	526 (2.1%)	526 (2.1%)	<0.001
Flu vaccination	44976 (86.0%)	111275 (85.9%)	0.003	21768 (85.3%)	21768 (85.3%)	<0.001
Number of primary care visits						
Mean (SD)	31.7 (17.4)	30.9 (17.7)	0.041	30.5 (17.1)	30.5 (17.1)	<0.001
Median (IQR)	28 (19-40)	27 (18-39)		27 (18-38)	27 (18-38)	
Number of Hospitalizations						
0	30667 (58.7%)	73454 (56.7%)	0.039	15251 (59.7%)	15251 (59.7%)	<0.001

1	6556 (12.5%)	16908 (13.1%)	-0.015	3122 (12.2%)	3122 (12.2%)	<0.001
2	2583 (4.9%)	6933 (5.4%)	-0.019	1173 (4.6%)	1173 (4.6%)	<0.001
≥3	1978 (3.8%)	6104 (4.7%)	-0.046	938 (3.7%)	938 (3.7%)	<0.001
Missing	10490 (20.1%)	26072 (20.1%)	-0.002	5048 (19.8%)	5048 (19.8%)	<0.001
Year of Entry						
2013	317 (0.6%)	13393 (10.3%)	-0.438	288 (1.1%)	288 (1.1%)	<0.001
2014	1537 (2.9%)	13331 (10.3%)	-0.299	1176 (4.6%)	1176 (4.6%)	<0.001
2015	2703 (5.2%)	14842 (11.5%)	-0.229	1960 (7.7%)	1960 (7.7%)	<0.001
2016	3141 (6.0%)	15723 (12.1%)	-0.215	2244 (8.8%)	2244 (8.8%)	<0.001
2017	3446 (6.6%)	15958 (12.3%)	-0.197	2438 (9.5%)	2438 (9.5%)	<0.001
2018	4177 (8.0%)	15676 (12.1%)	-0.137	2854 (11.2%)	2854 (11.2%)	<0.001
2019	5357 (10.2%)	13548 (10.5%)	-0.007	3248 (12.7%)	3248 (12.7%)	<0.001
2020	5138 (9.8%)	9391 (7.3%)	0.092	2780 (10.9%)	2780 (10.9%)	<0.001
2021	9296 (17.8%)	10027 (7.7%)	0.304	3995 (15.6%)	3995 (15.6%)	<0.001
2022	17162 (32.8%)	7582 (5.9%)	0.727	4550 (17.8%)	4550 (17.8%)	<0.001
Cerebrovascular disease	1807 (3.5%)	5383 (4.2%)	-0.037	892 (3.5%)	892 (3.5%)	<0.001
Peripheral artery disease or aortic atherosclerotic disease	1709 (3.3%)	4838 (3.7%)	-0.025	801 (3.1%)	801 (3.1%)	<0.001
Atrial fibrillation	5653 (10.8%)	12535 (9.7%)	0.037	2298 (9.0%)	2298 (9.0%)	<0.001
Myocardial infarction	2188 (4.2%)	3656 (2.8%)	0.074	797 (3.1%)	797 (3.1%)	<0.001
Angina	3480 (6.7%)	9498 (7.3%)	-0.027	1603 (6.3%)	1603 (6.3%)	<0.001
Heart failure	6245 (11.9%)	8582 (6.6%)	0.184	1968 (7.7%)	1968 (7.7%)	<0.001
Coronary artery disease	8770 (16.8%)	20124 (15.5%)	0.034	3704 (14.5%)	3704 (14.5%)	<0.001
Hypertension	29715 (56.8%)	76392 (59.0%)	-0.044	14365 (56.3%)	14365 (56.3%)	<0.001
Deep vein thrombosis	397 (0.8%)	1210 (0.9%)	-0.019	197 (0.8%)	197 (0.8%)	<0.001
Pulmonary embolism	414 (0.8%)	1041 (0.8%)	-0.001	188 (0.7%)	188 (0.7%)	<0.001
Hearing loss	2373 (4.5%)	7196 (5.6%)	-0.047	1203 (4.7%)	1203 (4.7%)	<0.001
Glaucoma	1274 (2.4%)	4176 (3.2%)	-0.048	657 (2.6%)	657 (2.6%)	<0.001
Cataract	4147 (7.9%)	13323 (10.3%)	-0.082	2077 (8.1%)	2077 (8.1%)	<0.001
Diabetic retinopathy	7141 (13.7%)	20097 (15.5%)	-0.053	3460 (13.6%)	3460 (13.6%)	<0.001
Psychotic disorders	421 (0.8%)	1509 (1.2%)	-0.036	228 (0.9%)	228 (0.9%)	<0.001
Bipolar disorder	164 (0.3%)	459 (0.4%)	-0.007	83 (0.3%)	83 (0.3%)	<0.001
Depression	4707 (9.0%)	10207 (7.9%)	0.040	2227 (8.7%)	2227 (8.7%)	<0.001
Delirium	260 (0.5%)	890 (0.7%)	-0.025	128 (0.5%)	128 (0.5%)	<0.001
Traumatic brain injury	124 (0.2%)	289 (0.2%)	0.003	56 (0.2%)	56 (0.2%)	<0.001
Parkinsonism	126 (0.2%)	552 (0.4%)	-0.032	74 (0.3%)	74 (0.3%)	<0.001
Sleep apnea	1851 (3.5%)	2743 (2.1%)	0.086	751 (2.9%)	751 (2.9%)	<0.001
Diabetic neuropathy	867 (1.7%)	2779 (2.1%)	-0.036	444 (1.7%)	444 (1.7%)	<0.001
Epilepsy	321 (0.6%)	1034 (0.8%)	-0.022	171 (0.7%)	171 (0.7%)	<0.001
Hypoglycemia	645 (1.2%)	2726 (2.1%)	-0.068	345 (1.4%)	345 (1.4%)	<0.001
Ketoacidosis	108 (0.2%)	253 (0.2%)	0.002	50 (0.2%)	50 (0.2%)	<0.001
Genital tract infection	1775 (3.4%)	3865 (3.0%)	0.023	829 (3.2%)	829 (3.2%)	<0.001
Urinary tract infection	2889 (5.5%)	12575 (9.7%)	-0.158	1654 (6.5%)	1654 (6.5%)	<0.001
Acute kidney injury	1920 (3.7%)	6991 (5.4%)	-0.083	953 (3.7%)	953 (3.7%)	<0.001
Chronic kidney disease	6298 (12.0%)	26122 (20.2%)	-0.222	3197 (12.5%)	3197 (12.5%)	<0.001
Kidney stones	880 (1.7%)	2343 (1.8%)	-0.010	425 (1.7%)	425 (1.7%)	<0.001
Pneumonia	1904 (3.6%)	4944 (3.8%)	-0.009	857 (3.4%)	857 (3.4%)	<0.001
Chronic obstructive pulmonary disease	4826 (9.2%)	12771 (9.9%)	-0.022	2343 (9.2%)	2343 (9.2%)	<0.001
Fractures	2057 (3.9%)	6425 (5.0%)	-0.050	1049 (4.1%)	1049 (4.1%)	<0.001
Osteoporosis	801 (1.5%)	3291 (2.5%)	-0.072	429 (1.7%)	429 (1.7%)	<0.001
Pancreatitis	329 (0.6%)	574 (0.4%)	0.025	137 (0.5%)	137 (0.5%)	<0.001

Non-alcoholic fatty liver disease	1791 (3.4%)	2902 (2.2%)	0.071	776 (3.0%)	776 (3.0%)	<0.001
Alcoholic liver disease	244 (0.5%)	466 (0.4%)	0.017	118 (0.5%)	118 (0.5%)	<0.001
Cirrhosis	357 (0.7%)	775 (0.6%)	0.011	173 (0.7%)	173 (0.7%)	<0.001
Inflammatory bowel disease	497 (1.0%)	1160 (0.9%)	0.006	235 (0.9%)	235 (0.9%)	<0.001
Cancer	4241 (8.1%)	13009 (10.0%)	-0.067	2186 (8.6%)	2186 (8.6%)	<0.001
Gout	2534 (4.8%)	6764 (5.2%)	-0.017	1144 (4.5%)	1144 (4.5%)	<0.001
Revascularization	6545 (12.5%)	13376 (10.3%)	0.069	2731 (10.7%)	2731 (10.7%)	<0.001
Lower extremity amputation	219 (0.4%)	685 (0.5%)	-0.016	110 (0.4%)	110 (0.4%)	<0.001
Metformin	45668 (87.4%)	111851 (86.4%)	0.029	22718 (89.0%)	22718 (89.0%)	<0.001
Thiazolidinediones	2768 (5.3%)	7728 (6.0%)	-0.029	1444 (5.7%)	1444 (5.7%)	<0.001
Sulfonylureas	15838 (30.3%)	54028 (41.7%)	-0.240	8700 (34.1%)	8700 (34.1%)	<0.001
Acarbose	50 (0.1%)	243 (0.2%)	-0.024	28 (0.1%)	28 (0.1%)	<0.001
Meglitinides	113 (0.2%)	470 (0.4%)	-0.027	68 (0.3%)	68 (0.3%)	<0.001
Insulin	5617 (10.7%)	7673 (5.9%)	0.175	2150 (8.4%)	2150 (8.4%)	<0.001
GLP1 receptor agonists	3884 (7.4%)	1993 (1.5%)	0.288	1026 (4.0%)	1026 (4.0%)	<0.001
Statins	43425 (83.1%)	105742 (81.7%)	0.037	20947 (82.0%)	20947 (82.0%)	<0.001
Fibrates	912 (1.7%)	2538 (2.0%)	-0.016	454 (1.8%)	454 (1.8%)	<0.001
Steroids	20530 (39.3%)	56051 (43.3%)	-0.082	10359 (40.6%)	10359 (40.6%)	<0.001
Nonsteroidal anti-inflammatory drugs	10454 (20.0%)	30767 (23.8%)	-0.091	5495 (21.5%)	5495 (21.5%)	<0.001
Aspirin	13785 (26.4%)	40121 (31.0%)	-0.102	6766 (26.5%)	6766 (26.5%)	<0.001
Anticoagulants	6919 (13.2%)	14493 (11.2%)	0.062	2829 (11.1%)	2829 (11.1%)	<0.001
Heparin	203 (0.4%)	868 (0.7%)	-0.039	115 (0.4%)	115 (0.4%)	<0.001
Antiplatelets	4948 (9.5%)	11524 (8.9%)	0.020	2178 (8.5%)	2178 (8.5%)	<0.001
Angiotensin converting enzyme inhibitors	25537 (48.9%)	61867 (47.8%)	0.021	12137 (47.5%)	12137 (47.5%)	<0.001
Angiotensin receptor blockers	12163 (23.3%)	28872 (22.3%)	0.023	5545 (21.7%)	5545 (21.7%)	<0.001
Beta-blockers	17635 (33.7%)	41274 (31.9%)	0.040	7805 (30.6%)	7805 (30.6%)	<0.001
Calcium channel blockers	19408 (37.1%)	50337 (38.9%)	-0.036	9565 (37.5%)	9565 (37.5%)	<0.001
Loop diuretics	8145 (15.6%)	20965 (16.2%)	-0.017	3478 (13.6%)	3478 (13.6%)	<0.001
Thiazide diuretics	8419 (16.1%)	25758 (19.9%)	-0.099	4413 (17.3%)	4413 (17.3%)	<0.001
Potassium sparing diuretics	4813 (9.2%)	6185 (4.8%)	0.174	1486 (5.8%)	1486 (5.8%)	<0.001
Digoxin	1674 (3.2%)	4717 (3.6%)	-0.024	736 (2.9%)	736 (2.9%)	<0.001
Methotrexate	447 (0.9%)	1251 (1.0%)	-0.012	231 (0.9%)	231 (0.9%)	<0.001
Hydroxychloroquine	200 (0.4%)	460 (0.4%)	0.005	93 (0.4%)	93 (0.4%)	<0.001
Allopurinol	3137 (6.0%)	7617 (5.9%)	0.005	1400 (5.5%)	1400 (5.5%)	<0.001
Colchicine	809 (1.5%)	2174 (1.7%)	-0.010	363 (1.4%)	363 (1.4%)	<0.001
Antibiotics	20384 (39.0%)	60139 (46.4%)	-0.151	10484 (41.1%)	10484 (41.1%)	<0.001
Antivirals	548 (1.0%)	1681 (1.3%)	-0.023	291 (1.1%)	291 (1.1%)	<0.001
Proton pump inhibitors	23529 (45.0%)	56829 (43.9%)	0.022	11181 (43.8%)	11181 (43.8%)	<0.001
H2 receptor antagonists	1274 (2.4%)	5449 (4.2%)	-0.099	770 (3.0%)	770 (3.0%)	<0.001
Tamoxifen	114 (0.2%)	335 (0.3%)	-0.008	59 (0.2%)	59 (0.2%)	<0.001
Aromatase inhibitors	391 (0.7%)	1134 (0.9%)	-0.014	204 (0.8%)	204 (0.8%)	<0.001
Chemotherapy	755 (1.4%)	2072 (1.6%)	-0.013	380 (1.5%)	380 (1.5%)	<0.001
Thyroid replacement therapy	5012 (9.6%)	13626 (10.5%)	-0.031	2519 (9.9%)	2519 (9.9%)	<0.001
Beta-3 agonist	422 (0.8%)	900 (0.7%)	0.013	204 (0.8%)	204 (0.8%)	<0.001

Bladder antimuscarinics	1711 (3.3%)	6090 (4.7%)	-0.073	958 (3.8%)	958 (3.8%)	<0.001
Antihistamines	4753 (9.1%)	13814 (10.7%)	-0.053	2481 (9.7%)	2481 (9.7%)	<0.001
Beta-2 agonists	10078 (19.3%)	25186 (19.5%)	-0.004	4912 (19.2%)	4912 (19.2%)	<0.001
Antimuscarinic bronchodilators	3581 (6.9%)	8578 (6.6%)	0.009	1698 (6.6%)	1698 (6.6%)	<0.001
Antiparkinsonian drugs (dopaminergic)	396 (0.8%)	1215 (0.9%)	-0.020	210 (0.8%)	210 (0.8%)	<0.001
Antiparkinsonian drugs (antimuscarinic)	86 (0.2%)	304 (0.2%)	-0.016	51 (0.2%)	51 (0.2%)	<0.001
Antipsychotics	1995 (3.8%)	6312 (4.9%)	-0.052	1072 (4.2%)	1072 (4.2%)	<0.001
Benzodiazepines	1512 (2.9%)	4949 (3.8%)	-0.052	821 (3.2%)	821 (3.2%)	<0.001
Nonbenzodiazepine hypnotics (Z-drugs)	1283 (2.5%)	4078 (3.1%)	-0.042	690 (2.7%)	690 (2.7%)	<0.001
Antiepileptic drugs	3156 (6.0%)	8215 (6.3%)	-0.013	1573 (6.2%)	1573 (6.2%)	<0.001
Selective serotonin reuptake inhibitors	6062 (11.6%)	12695 (9.8%)	0.058	2856 (11.2%)	2856 (11.2%)	<0.001
Tricyclic antidepressants	5358 (10.2%)	14463 (11.2%)	-0.030	2721 (10.7%)	2721 (10.7%)	<0.001
Other antidepressants	2994 (5.7%)	6378 (4.9%)	0.036	1417 (5.5%)	1417 (5.5%)	<0.001

Abbreviations: GLP1 = glucagon-like peptide-1; SGLT2 = sodium-glucose cotransporter 2; DPP4 = dipeptidyl peptidase-4; aSMD = Absolute standardized mean difference; SD = standard deviation; IQR = interquartile range. aSMDs ≥ 0.1 were considered as meaningful negligible between-group differences [2].

Supplementary Table 11. Full Baseline Characteristics of Initiators of GLP1 receptor agonists versus SGLT2 Inhibitors.

	Before weighting			After weighting		
**Characteristics**	**GLP1 (N=23400)**	**SGLT2 (N=92280)**	**SMD**	**GLP1 (N=14214)**	**SGLT2 (N=14214)**	**aSMD**
Age, years						
Mean (SD)	67.7 (6.1)	68.9 (6.8)	-0.177	67.9 (6.2)	67.9 (6.2)	<0.001
Median (IQR)	67 (63-72)	68 (63-73)		67 (63-72)	67 (63-72)	
Male	13010 (55.6%)	57631 (62.5%)	-0.140	8106 (57.0%)	8106 (57.0%)	<0.001
Country						
England	21969 (93.9%)	89551 (97.0%)	-0.152	13466 (94.7%)	13466 (94.7%)	<0.001
Northen Ireland	214 (0.9%)	632 (0.7%)	0.026	125 (0.9%)	125 (0.9%)	<0.001
Scotland	605 (2.6%)	960 (1.0%)	0.116	305 (2.1%)	305 (2.1%)	<0.001
Wales	612 (2.6%)	1137 (1.2%)	0.101	318 (2.2%)	318 (2.2%)	<0.001
Multiple Deprivation Index						
1	3431 (14.7%)	14185 (15.4%)	-0.020	2106 (14.8%)	2106 (14.8%)	<0.001
2	3297 (14.1%)	14010 (15.2%)	-0.031	2033 (14.3%)	2033 (14.3%)	<0.001
3	4381 (18.7%)	17751 (19.2%)	-0.013	2703 (19.0%)	2703 (19.0%)	<0.001
4	5351 (22.9%)	21633 (23.4%)	-0.014	3245 (22.8%)	3245 (22.8%)	<0.001
5	5509 (23.5%)	21972 (23.8%)	-0.006	3379 (23.8%)	3379 (23.8%)	<0.001
Outside of England	1431 (6.1%)	2729 (3.0%)	0.152	748 (5.3%)	748 (5.3%)	<0.001
Smoking						
Ever	19333 (82.6%)	74295 (80.5%)	0.054	11681 (82.2%)	11681 (82.2%)	<0.001
Never	4037 (17.3%)	17913 (19.4%)	-0.056	2512 (17.7%)	2512 (17.7%)	<0.001
Missing	30 (0.1%)	72 (0.1%)	0.016	20 (0.1%)	20 (0.1%)	<0.001
Diabetes duration, years						
Mean (SD)	11.8 (6.5)	10.8 (6.5)	0.153	11.4 (6.5)	11.4 (6.5)	<0.001
Median (IQR)	11.1 (7.0-15.7)	10.0 (5.9-14.8)		6.6 (10.7-15.4)	6.6 (10.7-15.4)	
Body Mass Index, kg/m²						
≤24.9	275 (1.2%)	8352 (9.1%)	-0.363	250 (1.8%)	250 (1.8%)	<0.001
25.0-29.9	2447 (10.5%)	26931 (29.2%)	-0.483	1960 (13.8%)	1960 (13.8%)	<0.001
≥30.0	19003 (81.2%)	48498 (52.6%)	0.639	10854 (76.4%)	10854 (76.4%)	<0.001
Missing	1675 (7.2%)	8499 (9.2%)	-0.075	1150 (8.1%)	1150 (8.1%)	<0.001
HbA1c, %						
≤7.0	1400 (6.0%)	9413 (10.2%)	-0.155	956 (6.7%)	956 (6.7%)	<0.001
7.1-8.0	3787 (16.2%)	22838 (24.7%)	-0.213	2582 (18.2%)	2582 (18.2%)	<0.001
>8.0	17796 (76.1%)	58135 (63.0%)	0.286	10418 (73.3%)	10418 (73.3%)	<0.001
Missing	417 (1.8%)	1894 (2.1%)	-0.020	257 (1.8%)	257 (1.8%)	<0.001
eGFR, mL/min/1.73m²						
≥90	8235 (35.2%)	38401 (41.6%)	-0.132	5451 (38.4%)	5451 (38.4%)	<0.001
60-90	9489 (40.6%)	41679 (45.2%)	-0.093	6104 (42.9%)	6104 (42.9%)	<0.001
45-60	3146 (13.4%)	6384 (6.9%)	0.217	1458 (10.3%)	1458 (10.3%)	<0.001
30-45	1601 (6.8%)	3338 (3.6%)	0.145	726 (5.1%)	726 (5.1%)	<0.001
<30	308 (1.3%)	872 (0.9%)	0.035	158 (1.1%)	158 (1.1%)	<0.001
Missing	621 (2.7%)	1606 (1.7%)	0.062	316 (2.2%)	316 (2.2%)	<0.001
Flu vaccination	20825 (89.0%)	79727 (86.4%)	0.079	12539 (88.2%)	12539 (88.2%)	<0.001
Number of primary care visits						
Mean (SD)	35.6 (19.1)	30.8 (16.9)	0.270	33.9 (18.3)	33.9 (18.2)	<0.001
Median (IQR)	32 (22-45)	27 (19-39)		30 (21-43)	21 (30-43)	
Number of Hospitalizations						
0	13655 (58.4%)	54409 (59.0%)	-0.012	8401 (59.1%)	8401 (59.1%)	<0.001
1	3007 (12.9%)	11474 (12.4%)	0.013	1791 (12.6%)	1791 (12.6%)	<0.001
2	1143 (4.9%)	4345 (4.7%)	0.008	671 (4.7%)	671 (4.7%)	<0.001

≥3	847 (3.6%)	3416 (3.7%)	-0.004	503 (3.5%)	503 (3.5%)	<0.001
Missing	4748 (20.3%)	18636 (20.2%)	0.002	2847 (20.0%)	2847 (20.0%)	<0.001
Year of Entry						
2013	2588 (11.1%)	553 (0.6%)	0.458	410 (2.9%)	410 (2.9%)	<0.001
2014	1989 (8.5%)	2622 (2.8%)	0.247	972 (6.8%)	972 (6.8%)	<0.001
2015	2001 (8.6%)	5355 (5.8%)	0.107	1256 (8.8%)	1256 (8.8%)	<0.001
2016	1760 (7.5%)	6137 (6.7%)	0.034	1167 (8.2%)	1167 (8.2%)	<0.001
2017	1890 (8.1%)	7062 (7.7%)	0.016	1248 (8.8%)	1248 (8.8%)	<0.001
2018	2087 (8.9%)	8552 (9.3%)	-0.012	1376 (9.7%)	1376 (9.7%)	<0.001
2019	2787 (11.9%)	10424 (11.3%)	0.019	1763 (12.4%)	1763 (12.4%)	<0.001
2020	2125 (9.1%)	9253 (10.0%)	-0.032	1420 (10.0%)	1420 (10.0%)	<0.001
2021	3355 (14.3%)	15524 (16.8%)	-0.069	2320 (16.3%)	2320 (16.3%)	<0.001
2022	2818 (12.0%)	26798 (29.0%)	-0.430	2282 (16.1%)	2282 (16.1%)	<0.001
Cerebrovascular disease	773 (3.3%)	3093 (3.4%)	-0.003	459 (3.2%)	459 (3.2%)	<0.001
Peripheral artery disease or aortic atherosclerotic disease	963 (4.1%)	2941 (3.2%)	0.050	522 (3.7%)	522 (3.7%)	<0.001
Atrial fibrillation	2000 (8.5%)	8628 (9.3%)	-0.028	1210 (8.5%)	1210 (8.5%)	<0.001
Myocardial infarction	606 (2.6%)	3365 (3.6%)	-0.061	383 (2.7%)	383 (2.7%)	<0.001
Angina	1693 (7.2%)	6079 (6.6%)	0.026	967 (6.8%)	967 (6.8%)	<0.001
Heart failure	1525 (6.5%)	8989 (9.7%)	-0.118	946 (6.7%)	946 (6.7%)	<0.001
Coronary artery disease	3636 (15.5%)	14670 (15.9%)	-0.010	2130 (15.0%)	2130 (15.0%)	<0.001
Hypertension	14184 (60.6%)	52448 (56.8%)	0.077	8486 (59.7%)	8486 (59.7%)	<0.001
Deep vein thrombosis	249 (1.1%)	651 (0.7%)	0.038	131 (0.9%)	131 (0.9%)	<0.001
Pulmonary embolism	216 (0.9%)	635 (0.7%)	0.026	118 (0.8%)	118 (0.8%)	<0.001
Hearing loss	1072 (4.6%)	4225 (4.6%)	0.000	646 (4.5%)	646 (4.5%)	<0.001
Glaucoma	546 (2.3%)	2352 (2.5%)	-0.014	324 (2.3%)	324 (2.3%)	<0.001
Cataract	1957 (8.4%)	8087 (8.8%)	-0.014	1168 (8.2%)	1168 (8.2%)	<0.001
Diabetic retinopathy	4325 (18.5%)	13906 (15.1%)	0.091	2417 (17.0%)	2417 (17.0%)	<0.001
Psychotic disorders	244 (1.0%)	771 (0.8%)	0.021	141 (1.0%)	141 (1.0%)	<0.001
Bipolar disorder	90 (0.4%)	275 (0.3%)	0.015	53 (0.4%)	53 (0.4%)	<0.001
Depression	2740 (11.7%)	7773 (8.4%)	0.109	1583 (11.1%)	1583 (11.1%)	<0.001
Delirium	107 (0.5%)	463 (0.5%)	-0.006	67 (0.5%)	67 (0.5%)	<0.001
Traumatic brain injury	46 (0.2%)	219 (0.2%)	-0.009	30 (0.2%)	30 (0.2%)	<0.001
Parkinsonism	74 (0.3%)	243 (0.3%)	0.010	38 (0.3%)	38 (0.3%)	<0.001
Sleep apnea	1423 (6.1%)	2623 (2.8%)	0.157	726 (5.1%)	726 (5.1%)	<0.001
Diabetic neuropathy	760 (3.2%)	1686 (1.8%)	0.090	384 (2.7%)	384 (2.7%)	<0.001
Epilepsy	196 (0.8%)	612 (0.7%)	0.020	109 (0.8%)	109 (0.8%)	<0.001
Hypoglycemia	517 (2.2%)	1307 (1.4%)	0.059	275 (1.9%)	275 (1.9%)	<0.001
Ketoacidosis	100 (0.4%)	167 (0.2%)	0.045	48 (0.3%)	48 (0.3%)	<0.001
Genital tract infection	1071 (4.6%)	3137 (3.4%)	0.060	612 (4.3%)	612 (4.3%)	<0.001
Urinary tract infection	2249 (9.6%)	5345 (5.8%)	0.144	1175 (8.3%)	1175 (8.3%)	<0.001
Acute kidney injury	1135 (4.9%)	3319 (3.6%)	0.062	603 (4.2%)	603 (4.2%)	<0.001
Chronic kidney disease	4531 (19.4%)	11675 (12.7%)	0.184	2275 (16.0%)	2275 (16.0%)	<0.001
Kidney stones	518 (2.2%)	1580 (1.7%)	0.036	289 (2.0%)	289 (2.0%)	<0.001
Pneumonia	809 (3.5%)	3122 (3.4%)	0.004	477 (3.4%)	477 (3.4%)	<0.001
Chronic obstructive pulmonary disease	2506 (10.7%)	7970 (8.6%)	0.070	1435 (10.1%)	1435 (10.1%)	<0.001
Fractures	1029 (4.4%)	3707 (4.0%)	0.019	609 (4.3%)	609 (4.3%)	<0.001
Osteoporosis	376 (1.6%)	1470 (1.6%)	0.001	224 (1.6%)	224 (1.6%)	<0.001
Pancreatitis	50 (0.2%)	499 (0.5%)	-0.053	38 (0.3%)	38 (0.3%)	<0.001
Non-alcoholic fatty liver disease	945 (4.0%)	3102 (3.4%)	0.036	579 (4.1%)	579 (4.1%)	<0.001
Alcoholic liver disease	99 (0.4%)	371 (0.4%)	0.003	58 (0.4%)	58 (0.4%)	<0.001
Cirrhosis	209 (0.9%)	607 (0.7%)	0.027	122 (0.9%)	122 (0.9%)	<0.001

Inflammatory bowel disease	171 (0.7%)	875 (0.9%)	-0.024	115 (0.8%)	115 (0.8%)	<0.001
Cancer	1962 (8.4%)	7466 (8.1%)	0.011	1171 (8.2%)	1171 (8.2%)	<0.001
Gout	1151 (4.9%)	4002 (4.3%)	0.028	669 (4.7%)	669 (4.7%)	<0.001
Revascularization	2471 (10.6%)	10962 (11.9%)	-0.042	1504 (10.6%)	1504 (10.6%)	<0.001
Lower extremity amputation	219 (0.9%)	383 (0.4%)	0.064	110 (0.8%)	110 (0.8%)	<0.001
Metformin	20375 (87.1%)	80871 (87.6%)	-0.017	12499 (87.9%)	12499 (87.9%)	<0.001
Thiazolidinediones	2090 (8.9%)	4871 (5.3%)	0.143	1088 (7.7%)	1088 (7.7%)	<0.001
Sulfonylureas	12235 (52.3%)	36095 (39.1%)	0.267	6931 (48.8%)	6931 (48.8%)	<0.001
Acarbose	58 (0.2%)	137 (0.1%)	0.022	28 (0.2%)	28 (0.2%)	<0.001
Meglitinides	131 (0.6%)	300 (0.3%)	0.035	61 (0.4%)	61 (0.4%)	<0.001
Insulin	5949 (25.4%)	7882 (8.5%)	0.461	2701 (19.0%)	2701 (19.0%)	<0.001
DPP4 inhibitors	11919 (50.9%)	40321 (43.7%)	0.145	7099 (49.9%)	7099 (49.9%)	<0.001
Statins	20072 (85.8%)	77499 (84.0%)	0.050	12065 (84.9%)	12065 (84.9%)	<0.001
Fibrates	630 (2.7%)	1657 (1.8%)	0.061	330 (2.3%)	330 (2.3%)	<0.001
Steroids	10750 (45.9%)	35875 (38.9%)	0.143	6260 (44.0%)	6260 (44.0%)	<0.001
Nonsteroidal anti-inflammatory drugs	5695 (24.3%)	18917 (20.5%)	0.092	3332 (23.4%)	3332 (23.4%)	<0.001
Aspirin	7509 (32.1%)	24929 (27.0%)	0.111	4174 (29.4%)	4174 (29.4%)	<0.001
Anticoagulants	2621 (11.2%)	10658 (11.5%)	-0.011	1571 (11.1%)	1571 (11.1%)	<0.001
Heparin	126 (0.5%)	325 (0.4%)	0.028	61 (0.4%)	61 (0.4%)	<0.001
Antiplatelets	2060 (8.8%)	8342 (9.0%)	-0.008	1229 (8.6%)	1229 (8.6%)	<0.001
Angiotensin converting enzyme inhibitors	12194 (52.1%)	44736 (48.5%)	0.073	7244 (51.0%)	7244 (51.0%)	<0.001
Angiotensin receptor blockers	5961 (25.5%)	21276 (23.1%)	0.056	3466 (24.4%)	3466 (24.4%)	<0.001
Beta-blockers	7764 (33.2%)	29588 (32.1%)	0.024	4574 (32.2%)	4574 (32.2%)	<0.001
Calcium channel blockers	9356 (40.0%)	34534 (37.4%)	0.053	5615 (39.5%)	5615 (39.5%)	<0.001
Loop diuretics	4382 (18.7%)	12331 (13.4%)	0.147	2261 (15.9%)	2261 (15.9%)	<0.001
Thiazide diuretics	5009 (21.4%)	14736 (16.0%)	0.140	2827 (19.9%)	2827 (19.9%)	<0.001
Potassium sparing diuretics	1407 (6.0%)	6619 (7.2%)	-0.047	801 (5.6%)	801 (5.6%)	<0.001
Digoxin	665 (2.8%)	2589 (2.8%)	0.002	376 (2.6%)	376 (2.6%)	<0.001
Methotrexate	245 (1.0%)	810 (0.9%)	0.017	146 (1.0%)	146 (1.0%)	<0.001
Hydroxychloroquine	113 (0.5%)	311 (0.3%)	0.023	62 (0.4%)	62 (0.4%)	<0.001
Allopurinol	1527 (6.5%)	4996 (5.4%)	0.047	875 (6.2%)	875 (6.2%)	<0.001
Colchicine	376 (1.6%)	1260 (1.4%)	0.020	212 (1.5%)	212 (1.5%)	<0.001
Antibiotics	11588 (49.5%)	35950 (39.0%)	0.214	6570 (46.2%)	6570 (46.2%)	<0.001
Antivirals	278 (1.2%)	1003 (1.1%)	0.010	169 (1.2%)	169 (1.2%)	<0.001
Proton pump inhibitors	11359 (48.5%)	41352 (44.8%)	0.075	6755 (47.5%)	6755 (47.5%)	<0.001
H2 receptor antagonists	869 (3.7%)	2360 (2.6%)	0.066	475 (3.3%)	475 (3.3%)	<0.001
Tamoxifen	53 (0.2%)	188 (0.2%)	0.005	32 (0.2%)	32 (0.2%)	<0.001
Aromatase inhibitors	206 (0.9%)	680 (0.7%)	0.016	125 (0.9%)	125 (0.9%)	<0.001
Chemotherapy	340 (1.5%)	1327 (1.4%)	0.001	209 (1.5%)	209 (1.5%)	<0.001
Thyroid replacement therapy	2890 (12.4%)	8574 (9.3%)	0.099	1621 (11.4%)	1621 (11.4%)	<0.001
Beta-3 agonist	276 (1.2%)	761 (0.8%)	0.036	164 (1.2%)	164 (1.2%)	<0.001
Bladder antimuscarinics	1389 (5.9%)	3131 (3.4%)	0.121	747 (5.3%)	747 (5.3%)	<0.001
Antihistamines	2809 (12.0%)	8961 (9.7%)	0.074	1584 (11.1%)	1584 (11.1%)	<0.001
Beta-2 agonists	5476 (23.4%)	16971 (18.4%)	0.123	3145 (22.1%)	3145 (22.1%)	<0.001
Antimuscarinic bronchodilators	1855 (7.9%)	5725 (6.2%)	0.067	1059 (7.5%)	1059 (7.5%)	<0.001
Antiparkinsonian drugs (dopaminergic)	263 (1.1%)	695 (0.8%)	0.038	147 (1.0%)	147 (1.0%)	<0.001

Antiparkinsonian drugs (antimuscarinic)	57 (0.2%)	163 (0.2%)	0.015	35 (0.2%)	35 (0.2%)	<0.001
Antipsychotics	1160 (5.0%)	3631 (3.9%)	0.050	664 (4.7%)	664 (4.7%)	<0.001
Benzodiazepines	933 (4.0%)	2680 (2.9%)	0.059	522 (3.7%)	522 (3.7%)	<0.001
Nonbenzodiazepine hypnotics (Z-drugs)	761 (3.3%)	2236 (2.4%)	0.050	420 (3.0%)	420 (3.0%)	<0.001
Antiepileptic drugs	2101 (9.0%)	5486 (5.9%)	0.116	1151 (8.1%)	1151 (8.1%)	<0.001
Selective serotonin reuptake inhibitors	3576 (15.3%)	9764 (10.6%)	0.140	2029 (14.3%)	2029 (14.3%)	<0.001
Tricyclic antidepressants	3468 (14.8%)	9318 (10.1%)	0.143	1933 (13.6%)	1933 (13.6%)	<0.001
Other antidepressants	1879 (8.0%)	5021 (5.4%)	0.103	1055 (7.4%)	1055 (7.4%)	<0.001

Abbreviations: GLP1 = glucagon-like peptide-1; SGLT2 = sodium-glucose cotransporter 2; DPP4 = dipeptidyl peptidase-4; aSMD = Absolute standardized mean difference; SD = standard deviation; IQR = interquartile range. aSMDs ≥ 0.1 were considered as meaningful negligible between-group differences [2].

Supplementary Table 12. Dementia Risks Associated with SGLT2 Inhibitors versus DPP4 Inhibitors, GLP1 receptor agonists versus DPP4 Inhibitors, and GLP1 Receptor Agonists versus SGLT2 Inhibitors Before Propensity Score Weighting.

Outcome	Exposure	N	Median Follow-Up Time [IQR], years [a]	Total Person-Years At Risk	Events	Rates [b]	IRD [95% CI] [b]	HR [95% CI]
**GLP1 receptor agonists versus DPP4 Inhibitors (Intention-to-Treat)**								
All-Cause Dementia	GLP1	22042	3.03 [4.99, 8.61]	114217.03	692	6.06	-3.88 [-4.38, -3.39]	0.61 [0.56, 0.65]
	DPP4	170517	3.58 [6.03, 8.90]	947698.90	9421	9.94	0 [Reference]	1 [Reference]
Alzheimer's disease	GLP1	22042	3.05 [5.03, 8.72]	115380.97	304	2.63	-2.09 [-2.41, -1.76]	0.56 [0.50, 0.62]
	DPP4	170517	3.64 [6.12, 9.00]	960926.65	4536	4.72	0 [Reference]	1 [Reference]
Vascular Dementia	GLP1	22042	3.05 [5.03, 8.74]	115606.33	242	2.09	-1.21 [-1.49, -0.92]	0.63 [0.55, 0.72]
	DPP4	170517	3.66 [6.15, 9.04]	964695.93	3183	3.30	0 [Reference]	1 [Reference]
**GLP1 receptor agonists versus DPP4 Inhibitors (As Treated)**								
All-Cause Dementia	GLP1	22042	1.31 [1.94, 3.07]	34502.07	115	3.33	-5.28 [-5.96, -4.59]	0.40 [0.33, 0.49]
	DPP4	170517	1.37 [2.12, 3.82]	346371.37	2982	8.61	0 [Reference]	1 [Reference]
Alzheimer's disease	GLP1	22042	1.31 [1.94, 3.08]	34590.74	40	1.16	-2.57 [-2.98, -2.16]	0.34 [0.25, 0.46]
	DPP4	170517	1.37 [2.14, 3.85]	349212.78	1302	3.73	0 [Reference]	1 [Reference]
Vascular Dementia	GLP1	22042	1.31 [1.94, 3.08]	34612.93	31	0.90	-1.70 [-2.06, -1.34]	0.37 [0.26, 0.53]
	DPP4	170517	1.37 [2.14, 3.86]	349927.56	908	2.59	0 [Reference]	1 [Reference]
**SGLT2 inhibitors versus DPP4 inhibitors (Intention-to-Treat)**								
All-Cause Dementia	SGLT2	52274	2.43 [3.46, 5.63]	169626.37	709	4.18	-4.91 [-5.29, -4.52]	0.48 [0.44, 0.52]
	DPP4	129471	3.44 [5.64, 7.91]	623640.70	5666	9.09	0 [Reference]	1 [Reference]
Alzheimer's disease	SGLT2	52274	2.44 [3.47, 5.65]	170407.76	290	1.70	-2.65 [-2.91, -2.40]	0.43 [0.38, 0.48]
	DPP4	129471	3.50 [5.69, 7.98]	630022.20	2743	4.35	0 [Reference]	1 [Reference]
Vascular Dementia	SGLT2	52274	2.44 [3.47, 5.65]	170506.87	274	1.61	-1.44 [-1.68, -1.21]	0.57 [0.51, 0.65]
	DPP4	129471	3.52 [5.71, 7.99]	632104.42	1929	3.05	0 [Reference]	1 [Reference]
**SGLT2 inhibitors versus DPP4 inhibitors (As Treated)**								
All-Cause Dementia	SGLT2	52274	1.28 [1.86, 2.67]	66325.03	216	3.26	-5.67 [-6.25, -5.09]	0.38 [0.33, 0.44]
	DPP4	129471	1.33 [2.02, 3.52]	229725.65	2051	8.93	0 [Reference]	1 [Reference]
Alzheimer's disease	SGLT2	52274	1.28 [1.86, 2.67]	66504.33	64	0.96	-2.91 [-3.25, -2.56]	0.28 [0.22, 0.36]
	DPP4	129471	1.34 [2.03, 3.55]	231339.72	895	3.87	0 [Reference]	1 [Reference]
Vascular Dementia	SGLT2	52274	1.28 [1.86, 2.67]	66507.39	65	0.98	-1.84 [-2.16, -1.51]	0.38 [0.30, 0.49]
	DPP4	129471	1.34 [2.04, 3.55]	231753.93	652	2.81	0 [Reference]	1 [Reference]
**GLP1 receptor agonists versus SGLT2 Inhibitors (Intention-to-Treat)**								
All-Cause Dementia	GLP1	23400	3.14 [4.96, 7.50]	105318.15	569	5.40	0.82 [0.32, 1.32]	1.09 [0.99, 1.21]
	SGLT2	92280	2.52 [3.69, 5.94]	315907.39	1447	4.58	0 [Reference]	1 [Reference]
Alzheimer's disease	GLP1	23400	3.16 [4.99, 7.55]	106099.62	248	2.34	0.25 [-0.09, 0.58]	0.99 [0.85, 1.15]
	SGLT2	92280	2.52 [3.70, 5.97]	317448.72	664	2.09	0 [Reference]	1 [Reference]

Vascular Dementia	GLP1	23400	3.16 [5.00, 7.56]	106233.66	202	1.90	0.28 [-0.02, 0.58]	1.03 [0.88, 1.22]
	SGLT2	92280	2.52 [3.71, 5.97]	317825.60	516	1.62	0 [Reference]	1 [Reference]
**GLP1 receptor agonists versus SGLT2 Inhibitors (As Treated)**								
All-Cause Dementia	GLP1	23400	1.30 [1.84, 2.94]	32522.37	128	3.94	0.33 [-0.43, 1.09]	1.09 [0.90, 1.33]
	SGLT2	92280	1.29 [1.89, 2.77]	125292.42	452	3.61	0 [Reference]	1 [Reference]
Alzheimer's disease	GLP1	23400	1.30 [1.85, 2.94]	32621.43	40	1.23	-0.12 [-0.55, 0.31]	0.91 [0.64, 1.28]
	SGLT2	92280	1.29 [1.89, 2.78]	125655.12	169	1.34	0 [Reference]	1 [Reference]
Vascular Dementia	GLP1	23400	1.30 [1.85, 2.94]	32620.82	45	1.38	0.36 [-0.08, 0.80]	1.34 [0.95, 1.88]
	SGLT2	92280	1.29 [1.89, 2.78]	125682.99	128	1.02	0 [Reference]	1 [Reference]

Abbreviations: GLP1 = glucagon-like peptide-1; SGLT2 = sodium-glucose cotransporter 2; DPP4 = dipeptidyl peptidase-4; IQR = interquartile range; IRD = incidence rate difference; HR = hazard ratio; CI = confidence interval.

a. Reported follow-up time started from cohort entry.

b. Incidence rates and IRDs per 1,000 person-years were estimated using a Poisson regression.

Supplementary Table 13. Summary of Endpoints.

	Before Weighting		After Weighting	
**GLP1 receptor agonists versus DPP4 inhibitors (intention-to-treat)**				
	**GLP1**	**DPP4**	**GLP1**	**DPP4**
All-cause dementia	692 (3.1%)	9421 (5.5%)	476 (3.5%)	506 (3.7%)
Death	4001 (18.2%)	39886 (23.4%)	2691 (19.6%)	2583 (18.9%)
End of registration	3272 (14.8%)	27235 (16.0%)	2131 (15.6%)	2189 (16.0%)
End of study period	14077 (63.9%)	93975 (55.1%)	8397 (61.3%)	8416 (61.5%)
**GLP1 receptor agonists versus DPP4 inhibitors (as treated)**				
All-cause dementia	115 (0.5%)	2982 (1.7%)	81 (0.6%)	120 (0.9%)
1 year after discontinuation	16482 (74.8%)	115786 (67.9%)	10337 (75.5%)	8809 (64.3%)
1 year after crossover	979 (4.4%)	10710 (6.3%)	708 (5.2%)	1706 (12.5%)
Death	713 (3.2%)	12812 (7.5%)	484 (3.5%)	634 (4.6%)
End of registration	839 (3.8%)	8663 (5.1%)	509 (3.7%)	583 (4.3%)
End of study period	2914 (13.2%)	19564 (11.5%)	1576 (11.5%)	1843 (13.5%)
	**Before Weighting**		**After Weighting**	
**SGLT2 inhibitors versus DPP4 inhibitors (intention-to-treat)**				
	**SGLT2**	**DPP4**	**SGLT2**	**DPP4**
All-cause dementia	709 (1.4%)	5666 (4.4%)	483 (1.9%)	558 (2.2%)
Death	3935 (7.5%)	26232 (20.3%)	2420 (9.5%)	2892 (11.3%)
End of registration	4748 (9.1%)	18807 (14.5%)	2923 (11.4%)	2891 (11.3%)
End of study period	42882 (82.0%)	78766 (60.8%)	19707 (77.2%)	19192 (75.2%)
**SGLT2 inhibitors versus DPP4 inhibitors (as treated)**				
All-cause dementia	216 (0.4%)	2051 (1.6%)	133 (0.5%)	207 (0.8%)
1 year after discontinuation	29653 (56.7%)	79181 (61.2%)	15354 (60.1%)	14300 (56.0%)
1 year after crossover	6250 (12.0%)	20373 (15.7%)	3721 (14.6%)	5228 (20.5%)
Death	1380 (2.6%)	9429 (7.3%)	732 (2.9%)	1061 (4.2%)
End of registration	1619 (3.1%)	6145 (4.7%)	865 (3.4%)	973 (3.8%)
End of study period	13156 (25.2%)	12292 (9.5%)	4727 (18.5%)	3764 (14.7%)
	**Before Weighting**		**After Weighting**	
**GLP1 receptor agonists versus SGLT2 inhibitors (intention-to-treat)**				
	**GLP1**	**SGLT2**	**GLP1**	**SGLT2**
All-cause dementia	569 (2.4%)	1447 (1.6%)	281 (2.0%)	289 (2.0%)
Death	3416 (14.6%)	7349 (8.0%)	1687 (11.9%)	1481 (10.4%)
End of registration	3054 (13.1%)	8528 (9.2%)	1659 (11.7%)	1646 (11.6%)
End of study period	16361 (69.9%)	74956 (81.2%)	10586 (74.5%)	10798 (76.0%)
**GLP1 receptor agonists versus SGLT2 inhibitors (as treated)**				
All-cause dementia	128 (0.5%)	452 (0.5%)	71 (0.5%)	74 (0.5%)
1 year after discontinuation	15607 (66.7%)	56409 (61.1%)	9252 (65.1%)	8939 (62.9%)

1 year after crossover	3809 (16.3%)	6966 (7.5%)	2333 (16.4%)	1676 (11.8%)
Death	791 (3.4%)	2577 (2.8%)	441 (3.1%)	432 (3.0%)
End of registration	751 (3.2%)	2987 (3.2%)	434 (3.1%)	484 (3.4%)
End of study period	2314 (9.9%)	22889 (24.8%)	1682 (11.8%)	2609 (18.4%)

Abbreviations: GLP1 = glucagon-like peptide-1; SGLT2 = sodium-glucose cotransporter 2; DPP4 = dipeptidyl peptidase-4.

Supplementary Table 14. Summary of Cohort Studies of Dementia Risk with GLP1 Receptor Agonists.

Study	Relative Dementia Risk	Causal Contrast	Potential Sources of Important Biases	Adjusted for Obesity
Zhou et al. (2021) [3]	0.90 (vs no use)	Unclear	The exposure was defined as exenatide use versus no use in 2007-2008. Because exenatide was a new drug at that time, exenatide might be given to certain patients that differed from those treated with other drugs. Non-users as a comparator for this question could introduce serious confounding by indication, especially channelling bias, and had a limited ability to inform clinical decision making.	No.
Nørgaard et al. (2022) [4]	0.89 (with yearly increased use)	Unclear	The cumulative exposure definition was defined based on data 5 year before the date of events or censoring. The follow-up started before exposure ascertainment. The cohort baseline violated a target trial emulation framework, which could introduce serious selection bias [5].	No.
Battini et al. (2024) [6]	0.63 (vs DPP4 inhibitors)	ITT	The outcome definition was only based on inpatient diagnoses. Because GLP1 receptor agonist users were less likely to be hospitalized [7], the findings with major cognitive impairment (e.g. dementia) might have been substantially overestimated by detection bias. Also, early symptoms or undiagnosed dementia might influence the decision to initiate one drug versus another [8], and therefore reverse causality could have been introduced.	Obesity indicator based on diagnostic codes
De Giorgi et al. (2024) [9]	0.52 (vs sitagliptin), 0.63 (vs glipizide)	ITT	The follow-up time was 1-year long. This first year of follow-up from treatment initiation was prone to reverse causality, because early symptoms or undiagnosed dementia might influence the decision to initiate one drug versus another [8].	Obesity indicator based on diagnostic codes
Wang et al. (2024) [10]	0.40 (vs DPP4 inhibitors)	ITT	Early symptoms or undiagnosed dementia might influence the decision to initiate one drug versus another [8], and therefore reverse causality could have been introduced. Also, the cohort selection approach might be susceptible to confounding by indication resulting from disease severity or related factors, but the characteristic tables for all comparisons were not reported to address the concern.	Restriction to those with obesity based on diagnostic codes
Tang et al. (2024) [11]	0.77 (vs DPP4 inhibitors), 0.69 (vs sulfonylureas)	ITT	**Potential sources of important biases were adequately addressed.** The results were robust to a 1-year lag time or longer, which was an analytical approach to mitigate reverse causality [12].	Obesity indicator based on diagnostic codes

Tang et al. (2025) [13]	0.67 (vs other second-line glucose-lowering drugs)	ITT	**Potential sources of important biases were adequately addressed.** A 0.5-year lag time still showed an estimate favouring GLP1 receptor agonists vs other second-line glucose-lowering medications. However, it was unclear whether the estimates were consistent in the sensitivity analyses with lag times longer than 0.5 years.	BMI from electronic health records.
Desai et al. (2025) [14]	0.74 (vs DPP4 inhibitors)	As Treated	**Potential sources of important biases were adequately addressed**. However, the number of events were too small to generate precise estimates.	Obesity indicator based on diagnostic codes
Xie et al. (2025) [15]	0.92 (vs usual care)	ITT	This study examined a series of outcomes. However, reverse causality could have been introduced to the analysis with dementia as an outcome, because early symptoms or undiagnosed dementia might influence the decision to initiate one drug versus another [8].	BMI from electronic health records.
Cohen et al. (2025) [16]	0.61 (vs older classes)	Unclear	The exposure was defined as use of GLP1 receptor agonists, SGLT2 inhibitors, DPP4 inhibitors, or older classes as a time-varying variable. The follow-up started from 1998, but GLP1 receptor agonists and the other two newer classes were not launched after 1998. First, serious confounding by indication, especially channeling bias, was a major concern about this study, because drugs newly on the market tended to be given to certain patients that differed from those treated with other drugs. Second, this comparison was highly confounded by calendar year, because users of older drugs were more likely to be those from an earlier cohort entry period. Third, a time-varying exposure definition had a limited ability to inform clinical decision making, because the baseline was unclear.	BMI from electronic health records.
Inoue et al. (2025) [17]	0.83 (vs DPP4 inhibitors)	ITT	**Potential sources of important biases were adequately addressed.** A 30-month risk ratio extrapolated from Kaplan-Meier curves was estimated. This method might slightly overestimate the effect size [18].	Obesity indicator based on diagnostic codes
Lin et al. (2025) [19]	0.63 (vs other glucose-lowering drugs)	Unclear (but likely ITT)	Early symptoms or undiagnosed dementia might influence the decision to initiate one drug versus another [8], and therefore reverse causality was a concern. Also, selection bias might be introduced by exclusion of GLP1 receptor agonist users within 3 months only in the control.	BMI from electronic health records.

Sun et al. (2025) [20]	0.90 (vs first-line metformin)	ITT	Time-varying covariates were directly adjusted in the model, which could block mediators on the causal pathway.	BMI from electronic health records.
Cheng et al. (2025) [21]	0.90 (vs other glucose-lowering drugs)	Unclear	Selection bias could be introduced by an exposure definition based on any GLP1 receptor agonist use during follow-up.	No.

Studies or analyses focusing on GLP1 receptor agonists versus SGLT2 inhibitors are not summarized in this table.

Supplementary Figure 1. Cohort Entry Flowchart.

GLP1 receptor agonists vs DPP4 inhibitors

Unique individuals prescribed GLP1 receptor agonists (expect weight loss formulations and oral semaglutide) or DPP4 inhibitors at the age of 60 years or older, from January 1st, 2007 to December 31st, 2022 **(N = 359,932)**

Cohort entry date = date of first prescription of either drug at the age of 60 years or older

Exclusion (N = 167,373)

1. 80,095: Continuous registration < 1 year in the past year (or unknown duration)
2. 41,800: Prescription of GLP1 receptor agonists or DPP4 inhibitors in the past year
3. 53: Prescription of GLP1 receptor agonists or DPP4 inhibitors at cohort entry
4. 242: Prescription of multiple GLP1 receptor agonists, weight loss formulations of semagluide or liraglutide, oral semaglutide, or multiple DPP4 inhibitors at cohort entry
5. 840: No record of type 2 diabetes or Hb1Ac ≥ 6.5% (48 mmol/mol) at any time prior to cohort entry
6. 12,322: Type 1 diabetes at any time prior
7. 357: A history of thyroid cancer or multiple endocrine neoplasia at any time prior
8. 2,212: Latest eGFR < 20 mL/min/1.73m² or dialysis in the past year, or end stage kidney disease at any time
9. 14,243: Dementia, cognitive deficits, or prescriptions for dementia at any time prior
10. 15,209: Dementia, cognitive deficits, or prescriptions for dementia within 1 year after cohort entry, or follow-up < 1 year

192,559 Eligible
- **22,042** GLP1 receptor agonist initiators
- **170,517** DPP4 inhibitor initiators

SGLT2 inhibitors vs DPP4 inhibitors

Unique individuals prescribed SGLT2 inhibitors or DPP4 inhibitors at the age of 60 years or older, from January 1st, 2013 to December 31st, 2022 **(N = 396,947)**

Cohort entry date = date of first prescription of either drug at the age of 60 years or older

Exclusion (N = 215,202)

1. 80,698: Continuous registration < 1 year in the past year (or unknown duration)
2. 74,604: Prescription of SGLT2 inhibitors or DPP4 inhibitors in the past year
3. 658: Prescription of SGLT2 inhibitors or DPP4 inhibitors at cohort entry
4. 266: Prescription of multiple SGLT2 inhibitors or multiple DPP4 inhibitors at cohort entry
5. 15,049: No record of type 2 diabetes or Hb1Ac ≥ 6.5% (48 mmol/mol) at any time prior to cohort entry
6. 10,992: Type 1 diabetes at any time prior
7. 389: A history of thyroid cancer or multiple endocrine neoplasia at any time prior
8. 2,123: Latest eGFR < 20 mL/min/1.73m² or dialysis in the past year, or end stage kidney disease at any time
9. 15,340: Dementia, cognitive deficits, or prescriptions for dementia at any time prior
10. 15,083: Dementia, cognitive deficits, or prescriptions for dementia within 1 year after cohort entry, or follow-up < 1 year

181,745 Eligible
- **52,274** SGLT2 inhibitor initiators
- **129,471** DPP4 inhibitor initiators

GLP1 receptor agonists vs SGLT2 inhibitors

Unique individuals prescribed GLP1 receptor agonists (expect weight loss formulations and oral semaglutide) or SGLT2 inhibitors at the age of 60 years or older, from January 1st, 2013 to December 31st, 2022 **(N = 217,395)**

Cohort entry date = date of first prescription of either drug at the age of 60 years or older

Exclusion (N = 101,715)

1. 31,037: Continuous registration < 1 year in the past year (or unknown duration)
2. 34,552: Prescription of GLP1 receptor agonists or SGLT2 inhibitors in the past year
3. 345: Prescription of GLP1 receptor agonists or SGLT2 inhibitors at cohort entry
4. 145: Prescription of multiple GLP1 receptor agonists, weight loss formulations of semagluide or liraglutide, oral semaglutide, or multiple DPP4 inhibitors at cohort entry
5. 15,020: No record of type 2 diabetes or Hb1Ac ≥ 6.5% (48 mmol/mol) at any time prior to cohort entry
6. 6,806: Type 1 diabetes at any time prior
7. 218: A history of thyroid cancer or multiple endocrine neoplasia at any time prior
8. 337: Latest eGFR < 20 mL/min/1.73m² or dialysis in the past year, or end stage kidney disease at any time
9. 6,401: Dementia, cognitive deficits, or prescriptions for dementia at any time prior
10. 6,854: Dementia, cognitive deficits, or prescriptions for dementia within 1 year after cohort entry, or follow-up < 1 year

115,680 Eligible
- **92,280** SGLT2 inhibitor initiators
- **23,400** GLP1 receptor agonist initiators

Supplementary Figure 2. Propensity Score Distribution of GLP1 Receptor Agonists versus DPP4 Inhibitors.

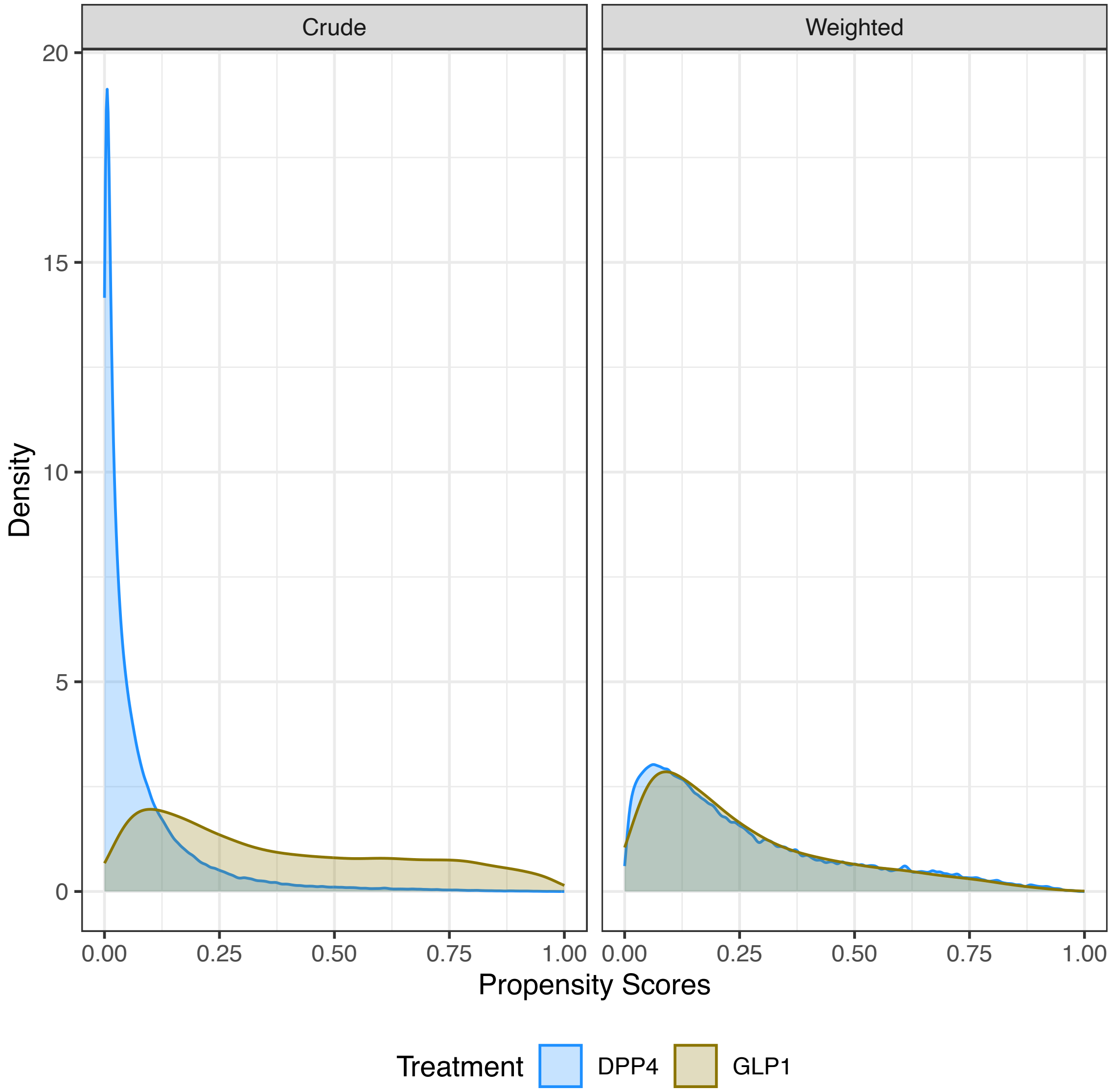

Supplementary Figure 3. Propensity Score Distribution of SGLT2 Inhibitors versus DPP4 Inhibitors.

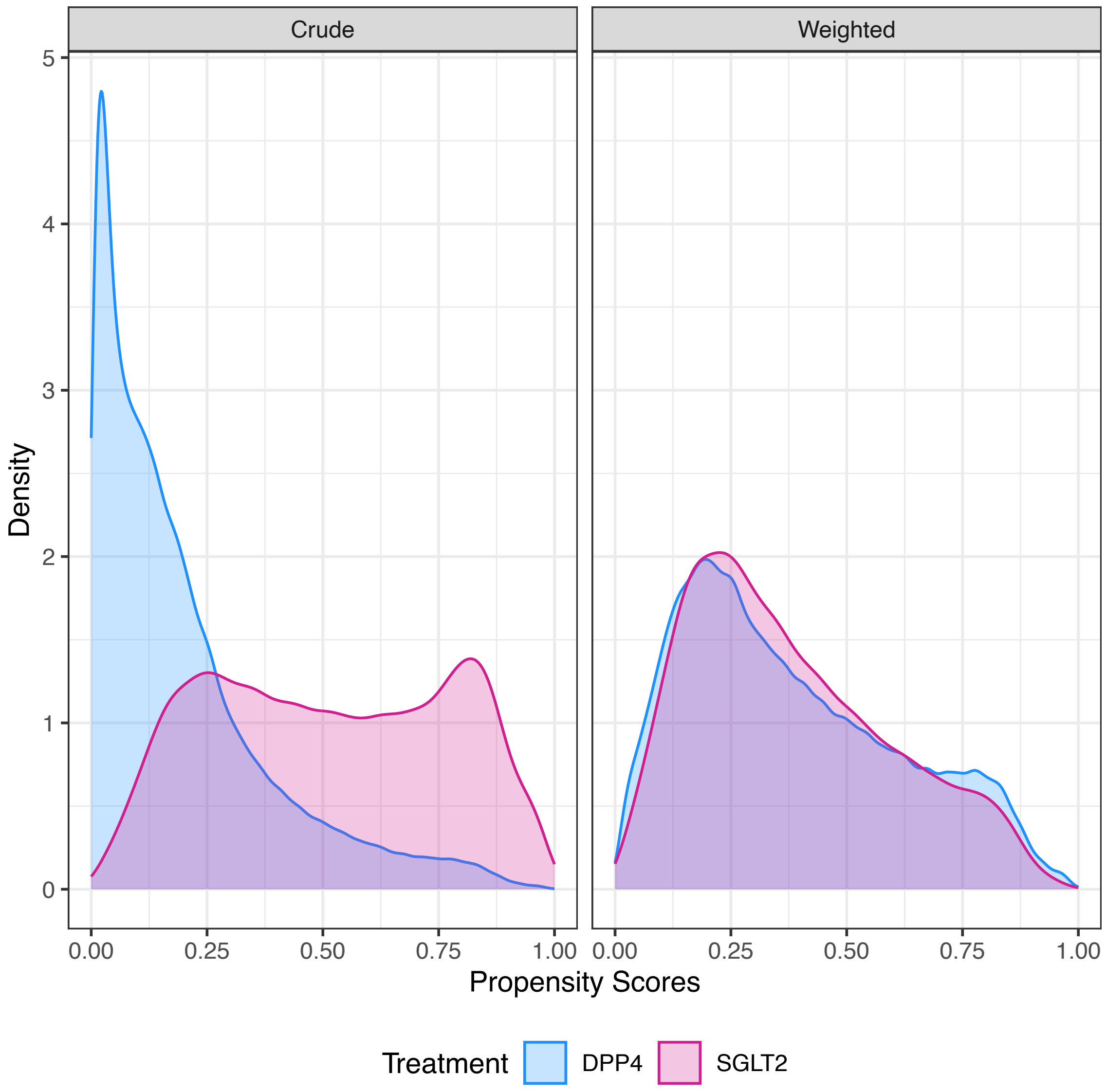

Supplementary Figure 4. Propensity Score Distribution of GLP1 Receptor Agonists versus SGLT2 Inhibitors.

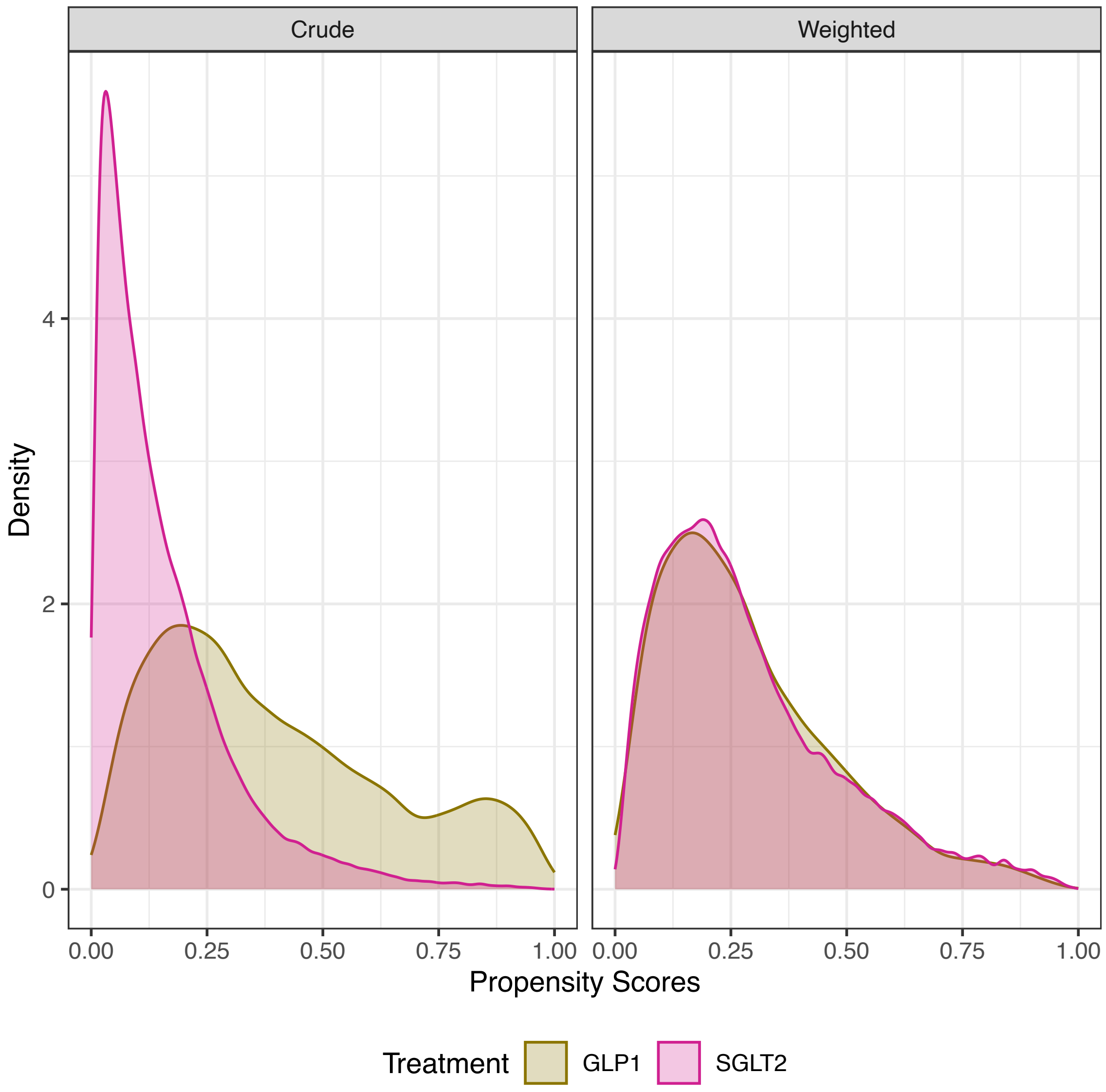

Supplementary Figure 5. Sensitivity Analyses of GLP1 Receptor Agonists versus DPP4 Inhibitors.

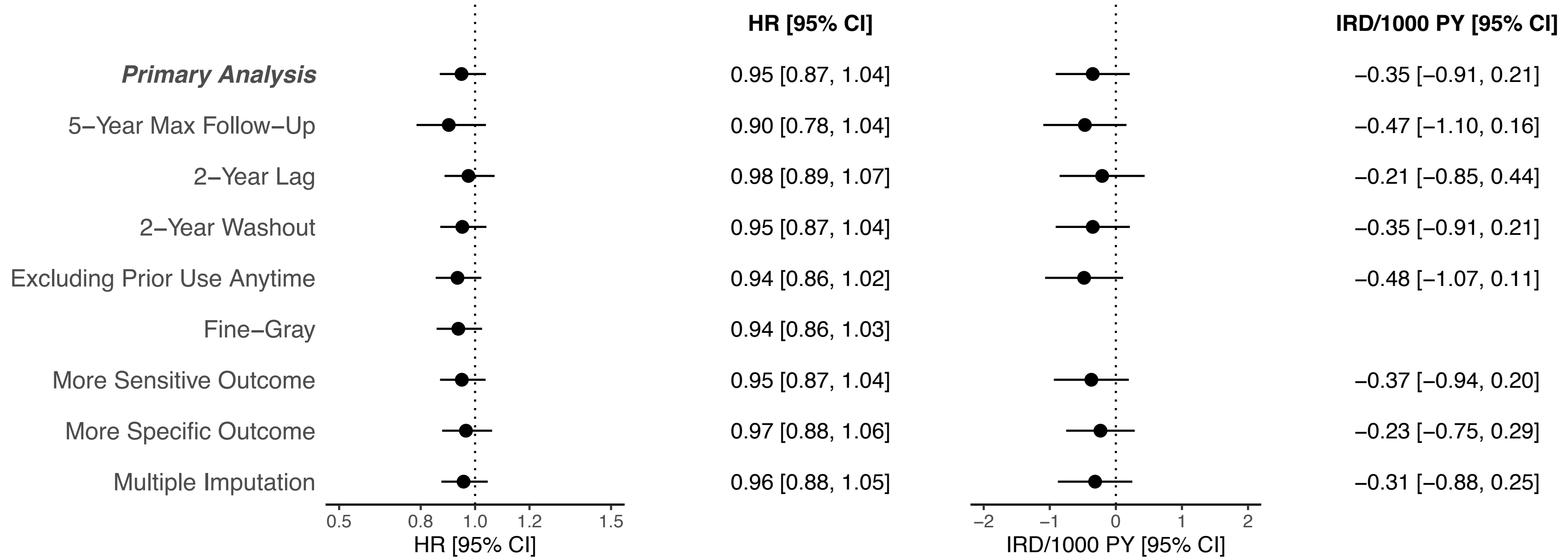

HR = hazard ratio; IRD = incidence rate difference; PY = person-years; CI = confidence interval.

Supplementary Figure 6. Sensitivity Analyses of SGLT2 Inhibitors versus DPP4 Inhibitors.

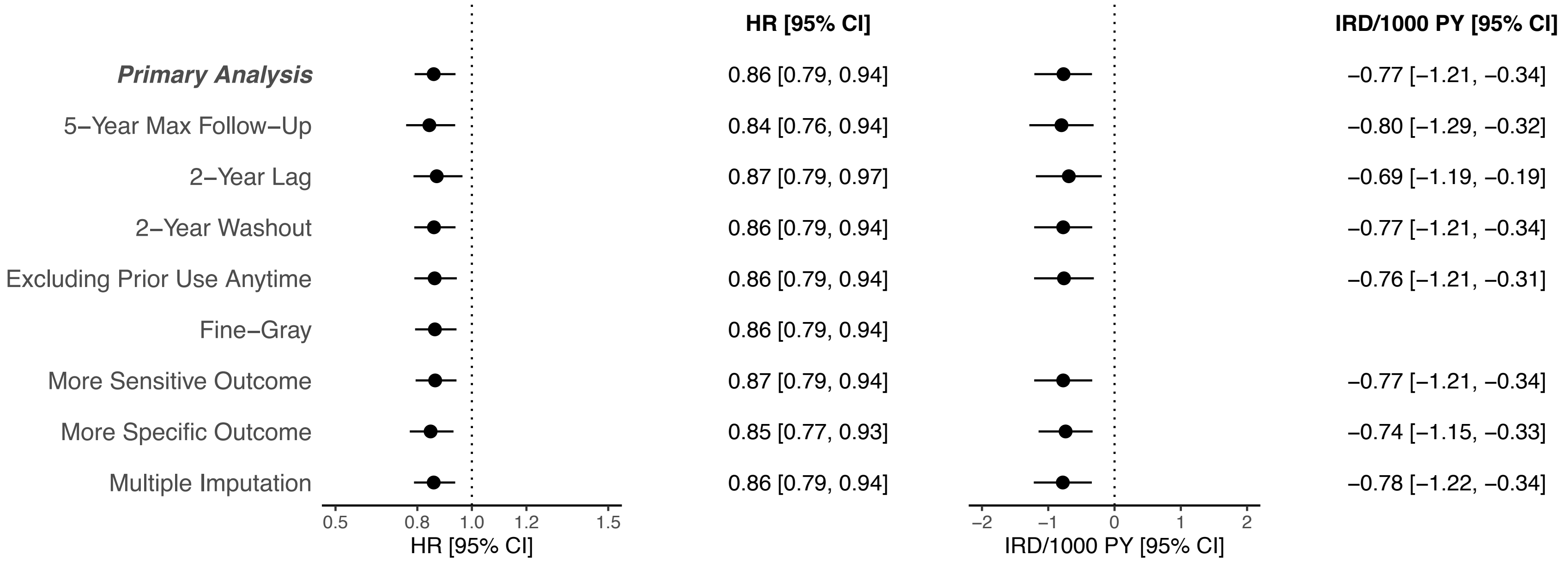

HR = hazard ratio; IRD = incidence rate difference; PY = person-years; CI = confidence interval.

Supplementary Figure 7. Sensitivity Analyses of GLP1 Receptor Agonists versus SGLT2 Inhibitors.

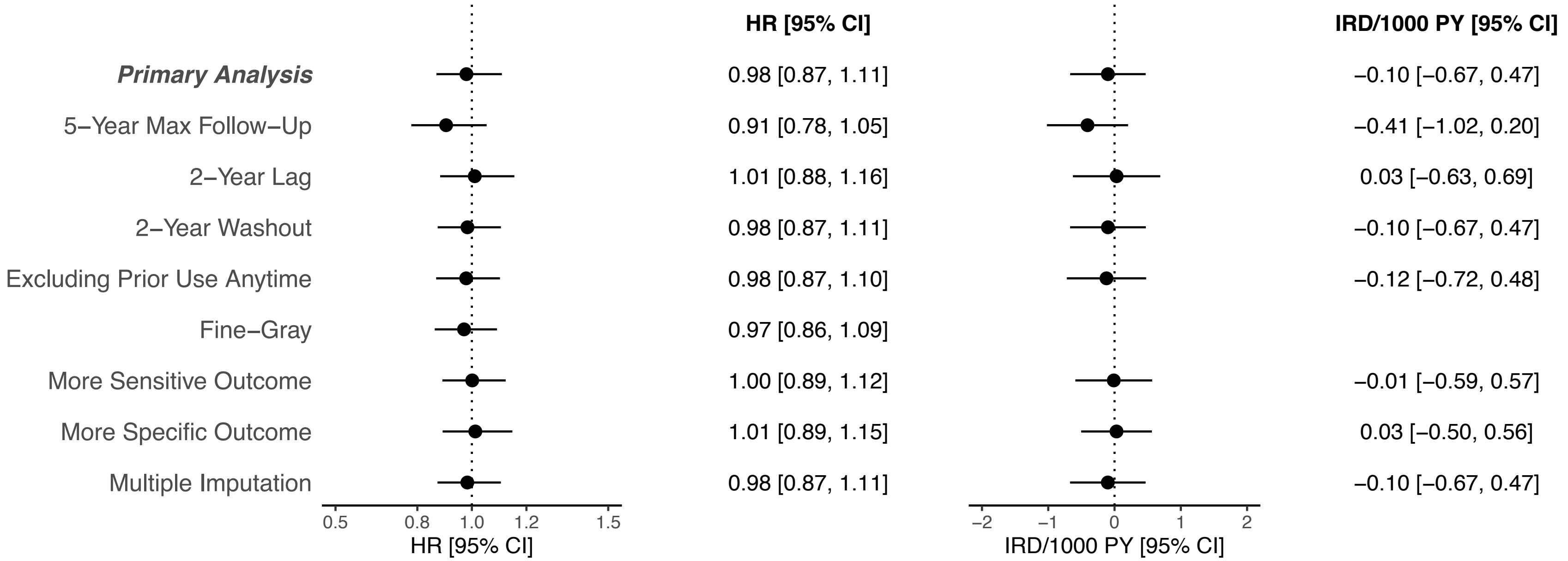

HR = hazard ratio; IRD = incidence rate difference; PY = person-years; CI = confidence interval.

Supplementary Figure 8. Quantitative Bias Analysis for GLP1 Receptor Agonists versus DPP4 Inhibitors.

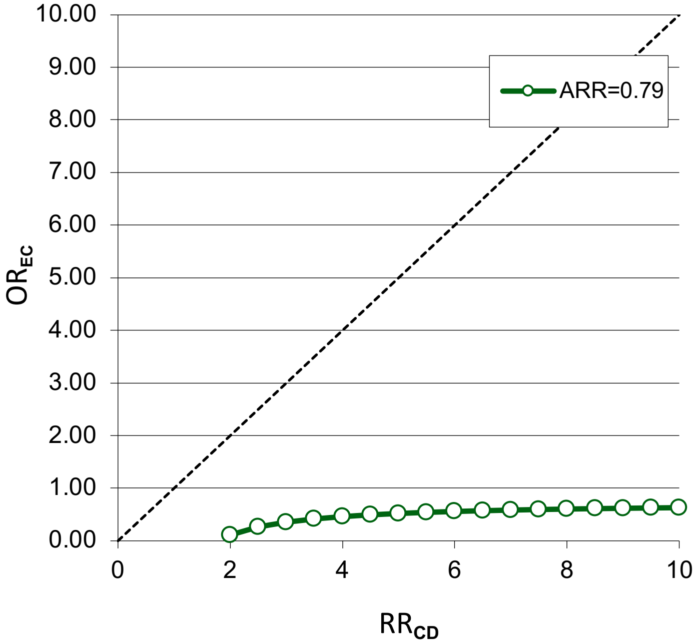

Abbreviations: RR_{CD} = Relative risk for the confounder-outcome relationship; OR_{EC} = Odds ratio for the exposure-confounder relationship. ARR = Apparent relative risk.

This analysis assumed a confounder prevalence of 25%, and the prevalence of exposure 50% due to the use of overlap weights. The line shows the minimum strength of confounder-outcome and exposure-confounder associations that an unmeasured confounder needed to yield an apparent relative risk of 0.79 (i.e., the observed hazard ratio) from a true null effect. The analysis suggested that a strong risk factor with a moderate between-group prevalence difference or a moderate risk factor with a strong between-group prevalence difference was needed to produce the association from a true null effect.

Supplementary Figure 9. Quantitative Bias Analysis for SGLT2 Inhibitors versus DPP4 Inhibitors.

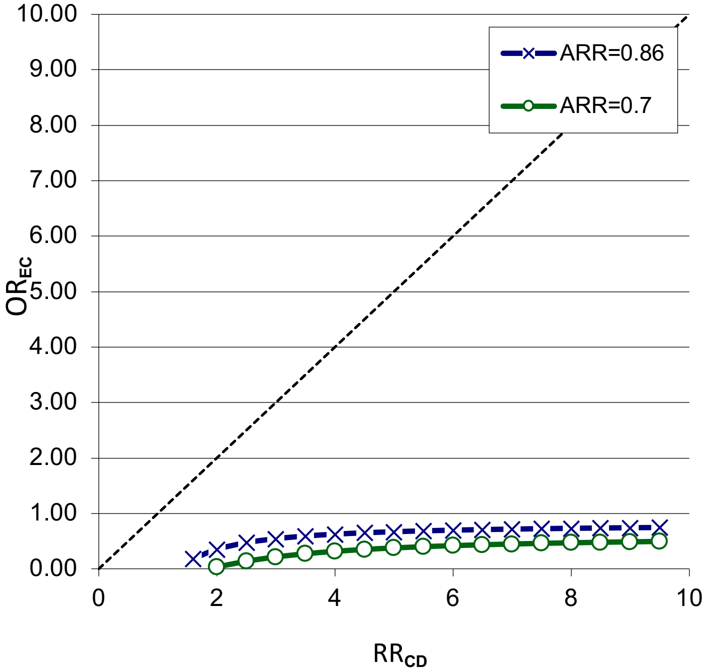

Abbreviations: RR_{CD} = Relative risk for the confounder-outcome relationship; OR_{EC} = Odds ratio for the exposure-confounder relationship. ARR = Apparent relative risk.

This analysis assumed a confounder prevalence of 25%, and the prevalence of exposure 50% due to the use of overlap weights. The line shows the minimum strength of confounder-outcome and exposure-confounder associations that an unmeasured confounder needed to yield an apparent relative risk of 0.70 or 0.86 (i.e., the observed hazard ratios for as-treated and intention-to-treat analyses, respectively) from a true null effect. For a relative risk of 0.70, the analysis suggested that a strong risk factor with a moderate between-group prevalence difference or a moderate risk factor with a strong between-group prevalence difference was needed to produce the association from a true null effect. However, for a relative risk of 0.86, a moderate unmeasured confounder with a moderate between-group prevalence difference, a strong confounder with a small between-group prevalence difference, or a weak confounder with a substantial between-group prevalence difference.

Supplementary References

1. Inker LA, Eneanya ND, Coresh J, et al. New Creatinine- and Cystatin C-Based Equations to Estimate GFR without Race. *N Engl J Med*. 2021;385(19):1737-1749. doi:10.1056/NEJMoa2102953

2. Austin PC, Xin Yu AY, Vyas M V, Kapral MK. Applying Propensity Score Methods in Clinical Research in Neurology. *Neurology*. 2021;97(18):856-863. doi:10.1212/WNL.0000000000012777

3. Zhou B, Zissimopoulos J, Nadeem H, Crane MA, Goldman D, Romley JA. Association between exenatide use and incidence of Alzheimer's disease. *Alzheimers Dement N Y N*. 2021;7(1):e12139. doi:10.1002/trc2.12139

4. Nørgaard CH, Friedrich S, Hansen CT, et al. Treatment with glucagon-like peptide-1 receptor agonists and incidence of dementia: Data from pooled double-blind randomized controlled trials and nationwide disease and prescription registers. *Alzheimers Dement N Y N*. 2022;8(1):e12268. doi:10.1002/trc2.12268

5. Hernán MA, Sauer BC, Hernández-Díaz S, Platt R, Shrier I. Specifying a target trial prevents immortal time bias and other self-inflicted injuries in observational analyses. *J Clin Epidemiol*. 2016;79:70-75. doi:10.1016/j.jclinepi.2016.04.014

6. Battini V, Barbieri MA, Carnovale C, Spina E, Clementi E, Sessa M. Comparing major and mild cognitive impairment risks in older type-2 diabetic patients: a Danish register-based study on dipeptidyl peptidase-4 inhibitors vs. glucagon-like peptide-1 analogues. *J Neurol*. 2024;271(6):3417-3425. doi:10.1007/s00415-024-12300-9

7. Alkabbani W, Gamble JM, Eurich DT, et al. Risk of hospitalization and death associated with sodium glucose cotransporter-2 inhibitors: A comparison with five other classes of antidiabetic drugs. *Diabetes Metab*. 2022;48(2):101305. doi:10.1016/j.diabet.2021.101305

8. Bradford A, Kunik ME, Schulz P, Williams SP, Singh H. Missed and delayed diagnosis of dementia in primary care: prevalence and contributing factors. *Alzheimer Dis Assoc Disord*. 2009;23(4):306-314. doi:10.1097/WAD.0b013e3181a6bebc

9. De Giorgi R, Koychev I, Adler AI, et al. 12-month neurological and psychiatric outcomes of semaglutide use for type 2 diabetes: a propensity-score matched cohort study. *EClinicalMedicine*. 2024;74:102726. doi:10.1016/j.eclinm.2024.102726

10. Wang W, Wang Q, Qi X, et al. Associations of semaglutide with first-time diagnosis of Alzheimer's disease in patients with type 2 diabetes: Target trial emulation using nationwide real-world data in the US. *Alzheimers Dement J Alzheimers Assoc*. 2024;20(12):8661-8672. doi:10.1002/alz.14313

11. Tang B, Sjölander A, Wastesson J, et al. Comparative effectiveness of glucagon-like peptide-1 agonists, dipeptidyl peptidase-4 inhibitors, and sulfonylureas on the risk of dementia in

older individuals with type 2 diabetes in Sweden: an emulated trial study. *eClinicalMedicine*. 2024;73:102689. doi:10.1016/j.eclinm.2024.102689

12. Tamim H, Monfared AAT, LeLorier J. Application of lag-time into exposure definitions to control for protopathic bias. *Pharmacoepidemiol Drug Saf*. 2007;16(3):250-258. doi:10.1002/pds.1360

13. Tang H, Donahoo WT, DeKosky ST, et al. GLP-1RA and SGLT2i Medications for Type 2 Diabetes and Alzheimer Disease and Related Dementias. *JAMA Neurol*. Published online April 2025. doi:10.1001/jamaneurol.2025.0353

14. Desai RJ, Varma VR, Mahesri M, et al. Population-Based Validation Results From the Drug Repurposing for Effective Alzheimer's Medicines (DREAM) Study. *Clin Pharmacol Ther*. 2025;117(4):1039-1050. doi:10.1002/cpt.3583

15. Xie Y, Choi T, Al-Aly Z. Mapping the effectiveness and risks of GLP-1 receptor agonists. *Nat Med*. 2025;31(3):951-962. doi:10.1038/s41591-024-03412-w

16. Cohen A, Levine SZ, Vainstein G, Beeri MS, Weinstein G. New-generation antidiabetic medications and dementia risk in older adults with type 2 diabetes: A retrospective cohort study. *J Prev Alzheimers Dis*. Published online 2025:100199. doi:https://doi.org/10.1016/j.tjpad.2025.100199

17. Inoue K, Saliba D, Gotanda H, et al. Glucagon-Like Peptide-1 Receptor Agonists and Incidence of Dementia Among Older Adults With Type 2 Diabetes : A Target Trial Emulation. *Ann Intern Med*. Published online July 2025. doi:10.7326/ANNALS-24-02648

18. Austin PC, Lee DS, Fine JP. Introduction to the Analysis of Survival Data in the Presence of Competing Risks. *Circulation*. 2016;133(6):601-609. doi:10.1161/CIRCULATIONAHA.115.017719

19. Lin HT, Tsai YF, Liao PL, Wei JCC. Neurodegeneration and Stroke After Semaglutide and Tirzepatide in Patients With Diabetes and Obesity. *JAMA Netw Open*. 2025;8(7):e2521016. doi:10.1001/jamanetworkopen.2025.21016

20. Sun M, Wang X, Lu Z, et al. Evaluating GLP-1 receptor agonists versus metformin as first-line therapy for reducing dementia risk in type 2 diabetes. *BMJ Open Diabetes Res Care*. 2025;13(4):e004902. doi:10.1136/bmjdrc-2025-004902

21. Cheng HW, Yang SF, Liao PL, Lee CH, Jong GP. Impact of Glucagon-Like Peptide-1 Receptor Agonists on the Dementia Incidence in Patients With Type 2 Diabetes Mellitus: A Population-Based Longitudinal Cohort Study. *Diabetes Metab Res Rev*. 2025;41(5):e70058. doi:10.1002/dmrr.70058
